# Network Pharmacology and *In Vitro* Experimental Verification Reveal the Mechanism of the Hirudin in Suppressing Myocardial Hypertrophy

**DOI:** 10.3389/fphar.2022.914518

**Published:** 2022-06-15

**Authors:** Mengnan Liu, Gang Luo, Li Dong, Maryam Mazhar, Li Wang, Wenlu He, Yan Liu, Qibiao Wu, Hua Zhou, Sijin Yang

**Affiliations:** ^1^ National Traditional Chinese Medicine Clinical Research Base and Department of Cardiovascular Medicine, the Affiliated Traditional Chinese Medicine Hospital of Southwest Medical University, Luzhou, China; ^2^ Faculty of Chinese Medicine and State Key Laboratory of Quality Research in Chinese Medicine, Macau University of Science and Technology, Macau, China; ^3^ National Traditional Chinese Medicine Clinical Research Base and Drug Research Center of the Affiliated Traditional Chinese Medicine Hospital of Southwest Medical University, Luzhou, China; ^4^ Research Center for Integrated Chinese and Western Medicine, the Affiliated Traditional Chinese Medicine Hospital of Southwest Medical University, Luzhou, China; ^5^ Sino-Portugal TCM International Cooperation Center, the Affiliated Traditional Chinese Medicine Hospital of Southwest Medical University, Luzhou, China; ^6^ Guangdong Provincial Hospital of Chinese Medicine, Guangdong Provincial Academy of Chinese Medical Sciences, State Key Laboratory of Dampness Syndrome of Chinese Medicine, Second Affiliated Hospital of Guangzhou University of Chinese Medicine, Guangdong-Hong Kong-Macau Joint Lab on Chinese Medicine and Immune Disease Research, Guangzhou, China

**Keywords:** network pharmacology, leech, hirudin, myocardial hypertrophy, molecular docking

## Abstract

**Background:** Myocardial hypertrophy is a complex pathological process, which is a common manifestation during the development of various cardiovascular diseases. Hirudin has been shown to have therapeutic effects on a variety of cardiovascular diseases, however, its therapeutic effect on myocardial hypertrophy is still unknown, and its chemical and pharmacological characteristics remain to be elucidated.

**Methods:** In this study, the network pharmacology method was used to characterize the mechanism of hirudin on myocardial hypertrophy. The potential protein targets of hirudin and myocardial hypertrophy were both obtained from the Genecards database, and potential pathways associated with genes were identified by Gene Ontology and pathway enrichment analysis, and the data were displayed in a visual manner. Subsequently, the potential mechanism of action of hirudin on myocardial hypertrophy predicted by network pharmacology analysis was verified by molecular docking, and finally, the main findings were further verified by *in vitro* experiments by molecular biology techniques. Based on the results obtained from the study of H9c2 cell line, the inhibitory effect of hirudin on myocardial hypertrophy was further proved in the primary rat cardiomyocytes.

**Results:** A total of 250 targets of hirudin, and 5,376 targets related to myocardial hypertrophy after deduplication were collected. The drug-disease network showed the relationship between hirudin, myocardial hypertrophy, and the targets. Further, systematic analysis from the PPI network indicated that blood coagulation, vesicle lumen, and signaling receptor activator activity may be the potential mechanisms of hirudin in the treatment of myocardial hypertrophy, and the PI3K/AKT signaling pathway may be the most relevant to the therapeutic effect of hirudin. Then, three therapeutic targets that were highly related to myocardial hypertrophy were extracted. Hirudin can be highly bound to STAT3, IL-6, and MAPK1 and found by molecular docking, which may be the basis for its inhibitory effect on myocardial hypertrophy. In addition, *in vitro* experiments showed that hirudin could inhibit AngII-induced hypertrophy and death of H9c2 cells, and significantly reduce the mRNA and protein expression levels of STAT3, MAPK1, and IL-6. The above conclusions were verified in primary rat cardiomyocytes.

**Conclusion:** Hirudin can be used to treat myocardial hypertrophy through a complex mechanism. The application of network pharmacology and experimental validation can promote the application of hirudin in cardiovascular diseases and the interpretation and understanding of molecular biological mechanisms.

## Highlights

1) Network pharmacological analysis showed that hirudin could play a therapeutic role in myocardial hypertrophy from multiple targets.
2) STAT3, IL-6, and MAPK1 can be used as the core targets of hirudin in the treatment of myocardial hypertrophy, showing good binding activity in molecular docking verification.
3) *In vitro* experiments showed that hirudin had a significant effect on inhibiting cardiac hypertrophy, which was consistent with the results predicted by network pharmacology analysis.

## Introduction

Cardiovascular diseases stand first out of the three most common causes of death in the world, which bring a huge psychological and economic burden to people (Mensah et al., 2019). Myocardial hypertrophy is a powerful form of compensation, however, with a certain limit. If the primary disease continues to grow and cannot be eliminated, the function of the hypertrophic myocardium cannot be maintained for a long time and eventually turns to heart failure (Gallo et al., 2019). Angiotensin II (AngII) is a major multifunctional active peptide in the renin-angiotensin-aldosterone system (RAAS), and it is also an important stimulator of myocardial hypertrophy (Zhou et al., 2016). It can directly lead to myocardial hypertrophy by binding to specific AngII receptors, which has a significant impact on the structure and function of the myocardium (Zhang et al., 2017). Chronic heart failure generally develops gradually on the basis of compensatory myocardial hypertrophy. Inhibiting or even reversing myocardial hypertrophy is a hot topic in the treatment of cardiovascular diseases (Schirone et al., 2017; Bacmeister et al., 2019).

Hirudin is mainly a polypeptide substance, isolated and purified from the saliva of leech, and it is the strongest natural anticoagulant among the known substances found in the world so far (Dong et al., 2016; Junren et al., 2021). Hirudin is a promising anticoagulant drug, a large number of studies have indicated that hirudin has the functions of inhibiting platelet aggregation, reducing blood lipids, regressing atherosclerosis, promoting the absorption of cerebral hematoma, and relieving intracranial pressure (Azzopardi et al., 2011; Bian et al., 2020; Junren et al., 2021; Nilius et al., 2021). In the treatment of cardiovascular diseases, the roles of hirudin in reducing inflammatory infiltration of vascular tissue, inhibiting the proliferation of vascular smooth muscle cells, improving endothelial dysfunction, regulating autophagy levels, resisting oxidative damage, and accelerating lipid metabolism have been gradually revealed (Dong et al., 2016; Yu et al., 2018; Wang et al., 2019; Zhang H. et al., 2020; Chen et al., 2021). However, the mechanism by which hirudin improves myocardial hypertrophy remains unclear. Therefore, there is a great interest in identifying the molecular targets of hirudin in myocardial hypertrophy. The purpose of this study was to clarify whether hirudin has therapeutic value for myocardial hypertrophy and to preliminarily explore its possible mechanism.

**FIGURE 1:**
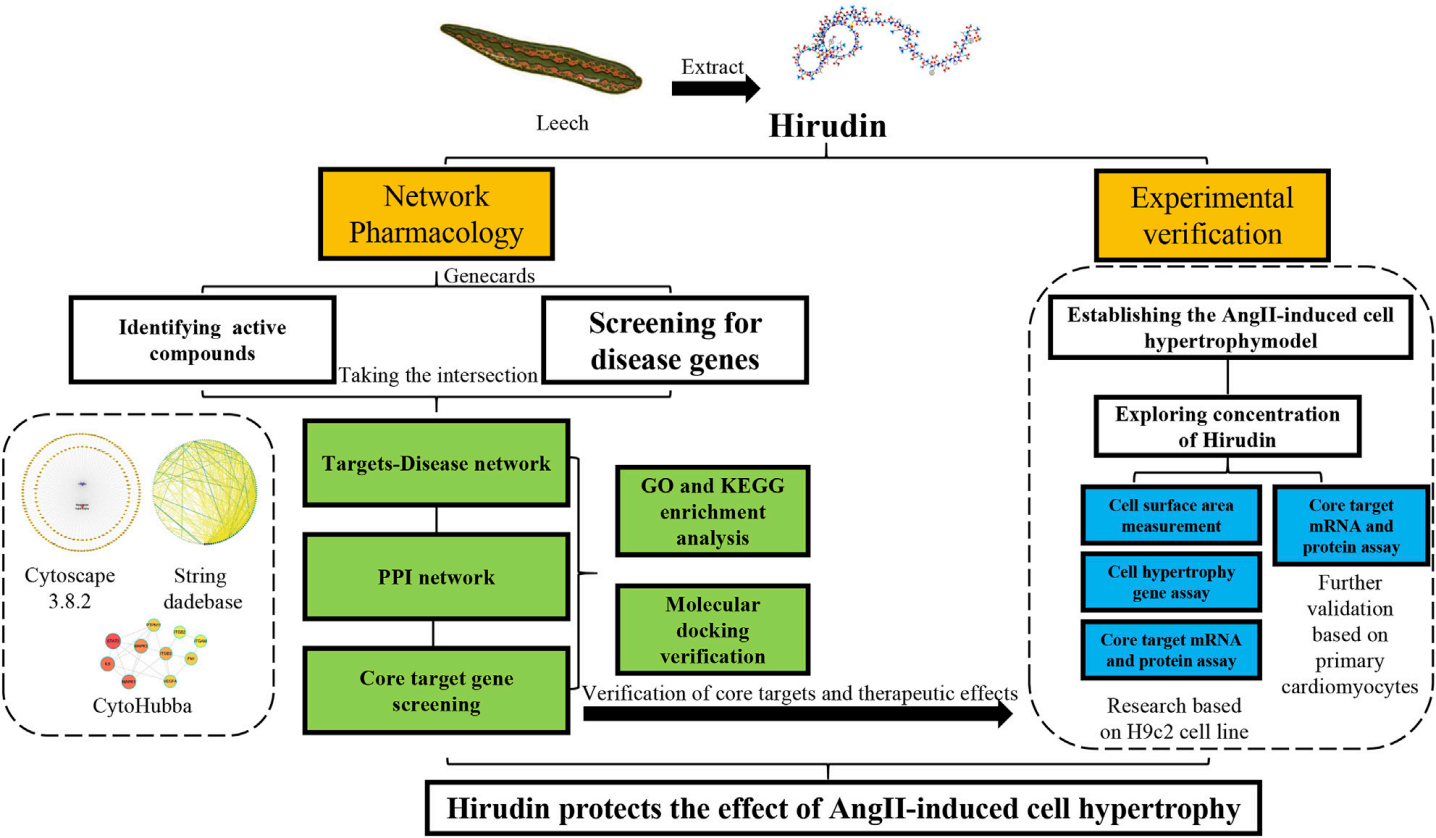
Workflow for dissecting the network pharmacology and *in vitro* experimental verification reveals the mechanism of the hirudin in suppressing myocardial hypertrophy.

The structure of hirudin is very complex, which means that it may have more potential roles in the treatment of diseases (Azzopardi et al., 2011; Dong et al., 2016), so it is suitable for network pharmacology to study. Network pharmacology aims to elucidate the relationship between compound-target-disease networks at the molecular level, which helps to elucidate the interactions between compounds, genes, proteins, and diseases (Li and Zhang, 2013; Zhang et al., 2019). Therefore, network pharmacology was used to find possible targets of hirudin for the treatment of myocardial hypertrophy, and molecular docking was used to verify the binding between hirudin and the core target. Finally, *in vitro* experiments using AngII induced H9c2 hypertrophy model was established to further reveal the potential mechanism of hirudin in the treatment of myocardial hypertrophy. This research provides a reference and theoretical basis for further experiments and clinical applications. The detailed workflow of this research was shown in Figure 1.

## Methods and Materials

### Network Pharmacology Analysis

#### Collection of Components and Targets

Hirudin targets were collected through the Genecards database (https://www.genecards.org/). Using the official name of the Uniprot database (https://www.uniprot.org/), the full name of the target gene was converted to the abbreviation, and the target without the corresponding gene name was deleted for use.

#### Disease Gene Collection

The disease-related genes were searched in the Genecards database, and the search terms included “myocardial hypertrophy” and “cardiac hypertrophy”, and then the above-predicted target genes were merged and deduplicated as disease-related gene data. Venny 2.1 was used to take intersections to obtain common targets and draw Venn diagrams.

#### Building a PPI Network

The common target dataset was imported into the STRING database (https://string-db.org/) to obtain the PPI network, and Cytoscape 3.8.2 was used for network visualization (Tang et al., 2015). The CytoNCA plug-in was used for network topology analysis, and the CytoHubba plug-in was used to screen key genes and construct key gene networks based on degree values (Chin et al., 2014).

#### Network Construction of Drug-Target

Cytoscape was used to construct a drug-target network. Each node in the network represents the active ingredient and the key target gene, respectively. The edge in the network was used to connect the active ingredient and the key target gene, and the nodes connected to the network were expressed in degrees.

#### GO and KEGG Enrichment Analysis

The gene IDs of the common drug-disease target genes were queried through the Bioconductor database (https://www.bioconductor.org/). R language was used to install the Bioconductor platform-related installation package, *p*-value = 0.05 and *q*-value = 0.05 were set for GO enrichment analysis and KEGG enrichment analysis. The top 5 GO processes and the top 20 KEGG pathways were drawn (Chen et al., 2017). Finally, Cytoscape was used to map to show the relationship between targets and pathways.

### Molecular Docking Verification

#### Molecular Docking Verification

The top 3 core targets with disease association degrees in the target-component network and the hirudin were used for molecular docking to verify the possibility between components and targets. The chemical and three-dimensional structures of the targets and components were retrieved from the Protein Data Bank (https://www.rcsb.org/) and PubChem database (https://pubchem.ncbi.nlm.nih.gov/), respectively. The Pymol software was used to dehydrate and dephosphorylate the protein molecules. Auto Dock 1.5.6 software was used to convert compounds and core proteins from pdb format to pdbqt format and search for active pockets. Finally, Auto Dock Tools was used to perform molecular docking (Ferreira et al., 2015).

### *In Vitro* Experimental Verification

#### Compound and UPLC-MS Analysis

Hirudin was purchased from Hefei Bomei Biotechnology Co., Ltd., and was evaluated for its purity which was found to be higher than 95%, using UPLC-MS analysis.

#### Cell Culture and Handling

H9c2 cells were purchased from Procell Life Science & Technology Co., Ltd. Cells were cultured in complete medium (1% streptomycin and penicillin, 10% fetal bovine serum, 89% Dulbecco’s Modified Eagle’s Medium) and maintained at 37°C and 95% air/5% CO_2_. The cells were passaged every 2–3 days according to the cell status.

#### Primary Cardiomyocyte Extraction

The extraction of neonatal rat cardiomyocytes was performed using previously established methods. Neonatal Sprague Dawley rats aged 0–3 days were sterilized and their chest cavity was opened, the heart was placed in a pre-cooled PBS and washed 3–4 times, the ventricle was cut into 1 mm^3^ tissue blocks, and then transferred into T25 cell culture flasks and incubated at 4°C with 2 ml of cell-digesting enzymes overnight. After 12 h, a solution of 10 mg type II collagenase (Roche, 5401119001), 100 mg BSA and 10 ml DMEM basal medium was used to digest the cells. After digestion, the supernatant was transferred to a 50 ml centrifuge tube, and an equal volume of complete medium was added to terminate the digestion, and all cell suspensions were filtered by a 200 mesh filter and centrifuged at 800 rpm for 3 min. After resuspension, the cells were inoculated into the cell culture dish for differential adhesion. After 2 h, the cells that did not adhere to the wall were identified as cardiomyocytes, and the cell density was adjusted to 1 × 10^5^/ml were inoculated into the dish and the medium was changed every 3 days, 0.1 mM 5-BrdU (Solarbio, B8010) was added to the medium to inhibit the proliferation of possible myocardial fibroblasts and the cells grown to 70% confluency were used for follow-up experiments. Immunofluorescence was used for cell identification.

#### Cell Viability Assessment

In order to find the optimal drug concentration, and determine whether hirudin has a toxic effect on H9c2 cells, 1 × 10^5^/ml cell suspension was prepared in a complete medium, and then inoculated into a 96-well cell culture plate after digestion, with a volume of 100 μl per well, and aspirate the original medium after culturing for 24 h. Pretreated with 0.01, 0.1, 1, or 10 μM AngII for 24 h, and screened out the optimal concentration of AngII, H9c2 cells were then incubated with hirudin at concentrations of 0.1, 0.6, and 1.2 mM to assess whether hirudin had a toxic effect on cells. Finally, the two drugs were co-cultured for 24 h in H9c2 cells. In the above experiments, 10 μl of CCK-8 solution was added to each well after the incubation for 4 h, and the absorbance of each well was measured at 450 nm.

Based on the results of the above experiments, we performed subsequent experiments by pretreating H9c2 cells with 1 μM AngII and 0.6, 1.2 mM hirudin for 24 h, respectively. Losartan was used as a positive control drug in the study at a concentration of 10 μM.

#### Phalloidin Staining

1 × 10^5^/ml cell suspension was prepared in advance and seeded in a 24-well plate, and 500 μl per well was grouped according to the results obtained in the above experiments. After 24 h of treatment, the medium was aspirated and washed 3 times, and the cells were fixed with 4% paraformaldehyde. After washing, the cells were treated with 0.1% Triton X-100 for 15 min and washed 3 times. SF488-labeled phalloidin was added and kept at room temperature for 40 min. Finally, DAPI was added, the images were acquired by an inverted fluorescence microscope, and the average area of a single cell was obtained by calculating the ratio of the green fluorescent area in the image and dividing by the number of nuclei in the image. The phalloidin reagent was purchased from Beijing Solarbio Science & Technology Co., Ltd., the product number is CA1640.

#### Immunofluorescence Staining

The procedure was similar to phalloidin staining. The treated cells were fixed, punched, and incubated overnight with primary antibody (cTnT, Cat. No. TD6261S. dilution 1:100). Subsequently, the cells were incubated with fluorescent secondary antibody AF594-conjugated Goat Anti-Rabbit IgG (dilution 1:100) for 1 h at room temperature in the dark. Finally, DAPI was added. Inverted fluorescence microscope was used to observe the cells and to acquired images. The above-mentioned antibodies were purchased from Abmart Co., Ltd.

#### RNA Isolation and Real-Time Quantitative Polymerase Chain Reaction

Total RNA was extracted from the samples by Trizol reagent according to the manufacturer’s instructions. To quantify the expression of the mRNA of interest, samples were run by SYBR Green assay (TOYOBO, Japan) according to the manufacturer’s protocol. GAPDH was used as a control, and the relative expression level of target mRNA was quantified by the 2^−ΔΔCt^ method. The primer sequences were shown in Table 1, and these primers were synthesized by Sangon Biotech (Shanghai) Co., Ltd.

**TABLE 1:** The primer sequence for qRT-PCR.

Primer’s name	Forward sequence 5’-3’	Reverse sequence 5’-3’
ANP	TAC​AGT​GCG​GTG​TCC​AAC​ACA​G	TGC​TTC​CTC​AGT​CTG​CTC​ACT​C
BNP	TCC​TAG​CCA​GTC​TCC​AGA​GCA​A	GGT​CCT​TCA​AGA​GCT​GTC​TCT​G
β-MHC	GCT​GGA​AGA​TGA​GTG​CTC​AGA​G	TCC​AAA​CCA​GCC​ATC​TCC​TCT​G
STAT3	AGG​AGT​CTA​ACA​ACG​GCA​GCC​T	GTG​GTA​CAC​CTC​AGT​CTC​GAA​G
IL-6	TAC​CAC​TTC​ACA​AGT​CGG​AGG​C	CTG​CAA​GTG​CAT​CAT​CGT​TGT​TC
MAPK1	TCA​AGC​CTT​CCA​ACC​TCC​TGC​T	AGC​TCT​GTA​CCA​ACG​TGT​GGC​T
GAPDH	GGA​CCT​CAT​GGC​CTA​CAT​GG	TAG​GGC​CTC​TCT​TGC​TCA​GT

#### Enzyme-Linked Immunosorbent Assay

The concentrations of cytokines IL-6 in cell supernatant from all groups were measured using a commercially available ELISA kit. Briefly, ELISA assay was performed according to the manufacturer’s instructions. The ELISA kit were purchased from Abmart Co., Ltd. with Cat. No. AB-1731A.

#### Western Blotting Analysis

RIPA (Beyotime, China) buffer was used to lyse samples. SDS-PAGE was used to separate lysates and transfer proteins to NC membranes. Membranes were then blocked with 5% BSA for 1 h at room temperature, followed by overnight incubation at 4°C with primary antibodies, all at a 1:1000 dilution. Antibodies included STAT3, p-STAT3 (Tyr705), IL-6, MAPK1, p-MAPK1 (Thr202/Tyr204) and β-tubulin (Cat. Nos. AF6294, AF3293, DF6087, AF0155, AF1015, DF7967, respectively). After overnight incubation with the primary antibody, the secondary antibody (1:5,000) was added and incubated at room temperature for 1 h. After washing again, the Amersham Typhoon Multispectral Laser Imager was used to acquire images and analyze the gray values of the bands. The above-mentioned antibodies were purchased from Affinity Biosciences Co., Ltd.

#### Statistical Analysis

GraphPad Prism 9.1.2 software was used for statistical analysis. Data were presented as mean ± standard deviation (SD). Among them, in order to compare the differences between multiple groups, the one-way ANOVA was used when the data conformed to the normal distribution, and the rank-sum test was used when the data did not conform to the normal distribution. Statistical significance was defined as *p < 0.05, **p < 0.01.

## Result

### Targets of Hirudin and Myocardial Hypertrophy-Related Targets

A total of 250 hirudin targets were collected by searching the Genecards database. For myocardial hypertrophy, after searching “myocardial hypertrophy” and “cardiac hypertrophy”, 2,909 and 5,352 targets were collected, respectively. After combining these targets, 5,376 targets were obtained. The resulting target information is presented in Supplementary Table S1.

### Drug-Target Interaction Network Analysis

**FIGURE 2:**
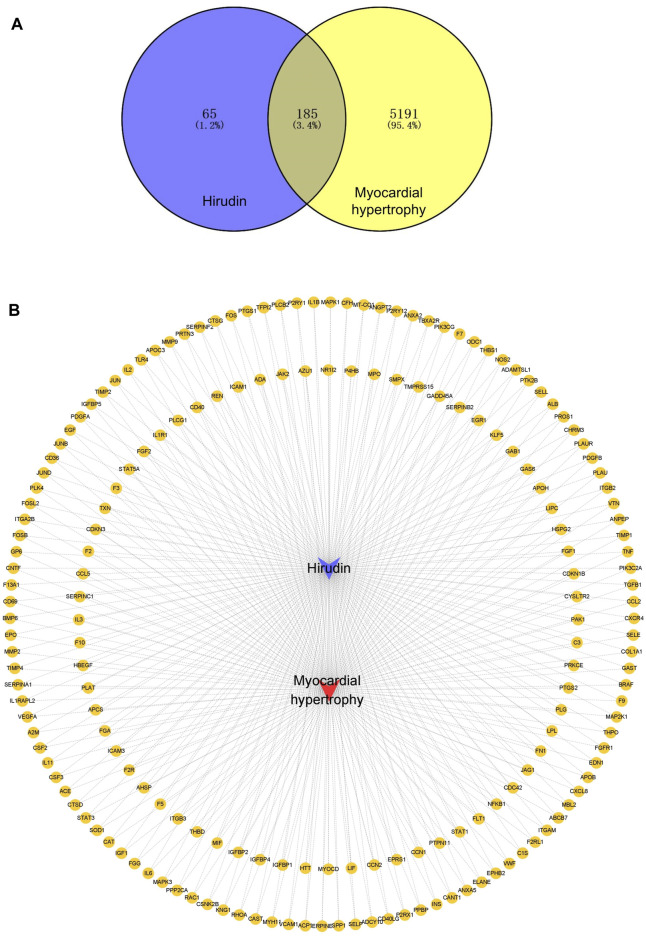
Venn diagram of myocardial hypertrophy targets and hirudin targets and their network relationships. **(A)** Venn diagram of targets. **(B)** Network diagram of compound-target-disease, the yellow circles represent genes, the purple V shape represents hirudin and the red V shape represents myocardial hypertrophy.

**FIGURE 3:**
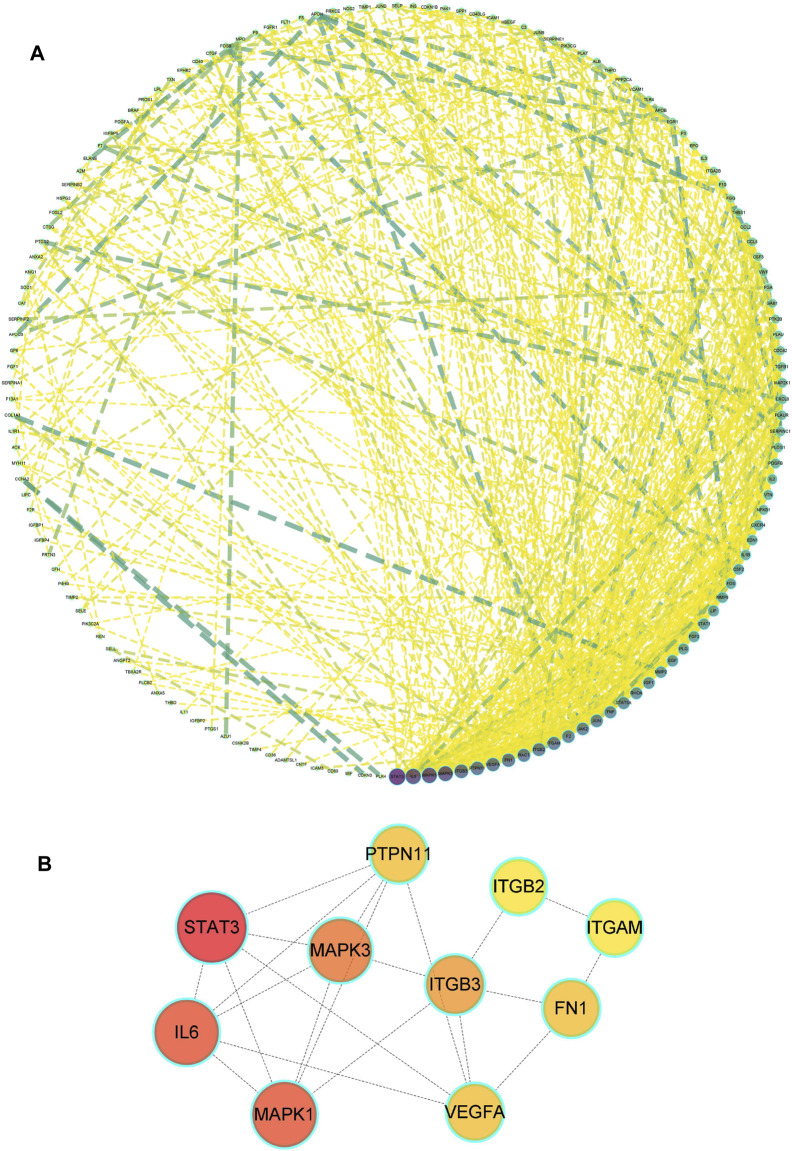
Drug-disease PPI network. **(A)** The network contains 185 gene nodes and 3,656 edges. Yellow circles represent gene targets. Purple nodes have a higher degree. The node size of the gene target is proportional to the degree. **(B)** The hub network was obtained by screening.

A total of 185 overlapping genes were obtained by finding the intersections of the above composite targets and disease targets (Figure 2A and Supplementary Table S2), and Cytoscape was used to construct a drug-target network (Figure 2B). By feeding common target data into STRING, we obtained a drug-target PPI network with connectivity (interaction score ≥ 0.400), containing 185 nodes and 3,656 edges (Supplementary Figure S1). Based on the 3 network parameters of “degree”, “betweenness” and “proximity”, the exported analysis file was visualized in Cytoscape (Figure 3A). The CytoNCA plugin was used for network topology analysis, and the CytoHubba plugin was used to screen key genes based on degree values (Figure 3B and Supplementary Table S3). In building key gene networks in the hub network, nodes interact with many other edges (32 in STAT3, 29 in IL-6 and MAPK1 each, 28 in MAPK3, 25 in ITGB3, 24 each in FN1, VEGFA, and PTPN11, and 23 each in EITGB2 and ITGAM). These results suggest that these highly pivotal targets may explain the fundamental therapeutic role of hirudin in myocardial hypertrophy.

### GO Biological Process and KEGG Pathway Enrichment Analysis

**FIGURE 4:**
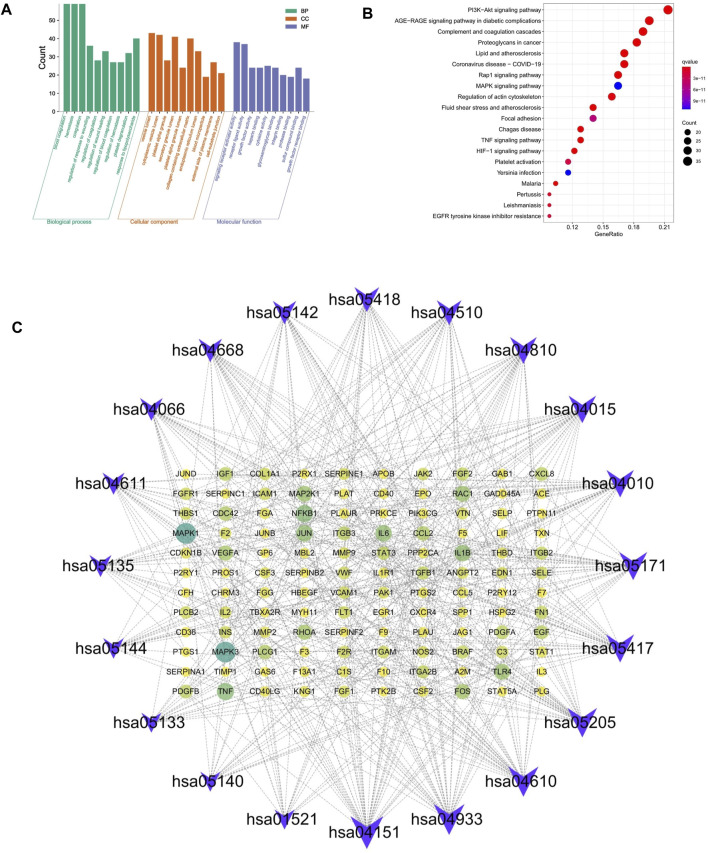
**(A)** The GO and KEGG pathway enrichment. **(B)** Analysis of 20 therapeutic signaling pathways of hirudin on myocardial hypertrophy. **(C)** The purple V shape represents the signaling pathway, the yellow circle represents the target, and the size of the shape and the depth of the color represent more intersections.

To explore the therapeutic mechanism of hirudin on putative targets of myocardial hypertrophy, GO and KEGG pathway enrichment analysis was performed using DAVID 6.8. With a total of 2,149 biological process (BP), 53 cellular components (CC), and 145 molecular function (MF) terms (Supplementary Table S4), the top 5 significantly enriched GO terms in BP, CC, and MF were plotted in Figure 4A, suggesting that hirudin may exert its cardioprotective effect by regulating myocardial hypertrophy by interacting with blood coagulation, vesicle lumen, and signaling receptor activator activity. To further reveal the underlying mechanism of hirudin-related pathways in the treatment of myocardial hypertrophy, KEGG pathway enrichment analysis was performed on related targets (Supplementary Table S5). The 20 most significantly enriched pathways in myocardial hypertrophy associated with hirudin therapeutic targets were presented in Figure 4B. Then, the targets and corresponding signaling pathways were imported into Cytoscape to draw a target-pathway network diagram. The relationship between the number of targets and pathway enrichment was shown in Figure 4C. The results also showed that the PI3K/AKT signaling pathway was the most abundant pathway, involving 35 targets.

### Molecular Docking

**FIGURE 5:**
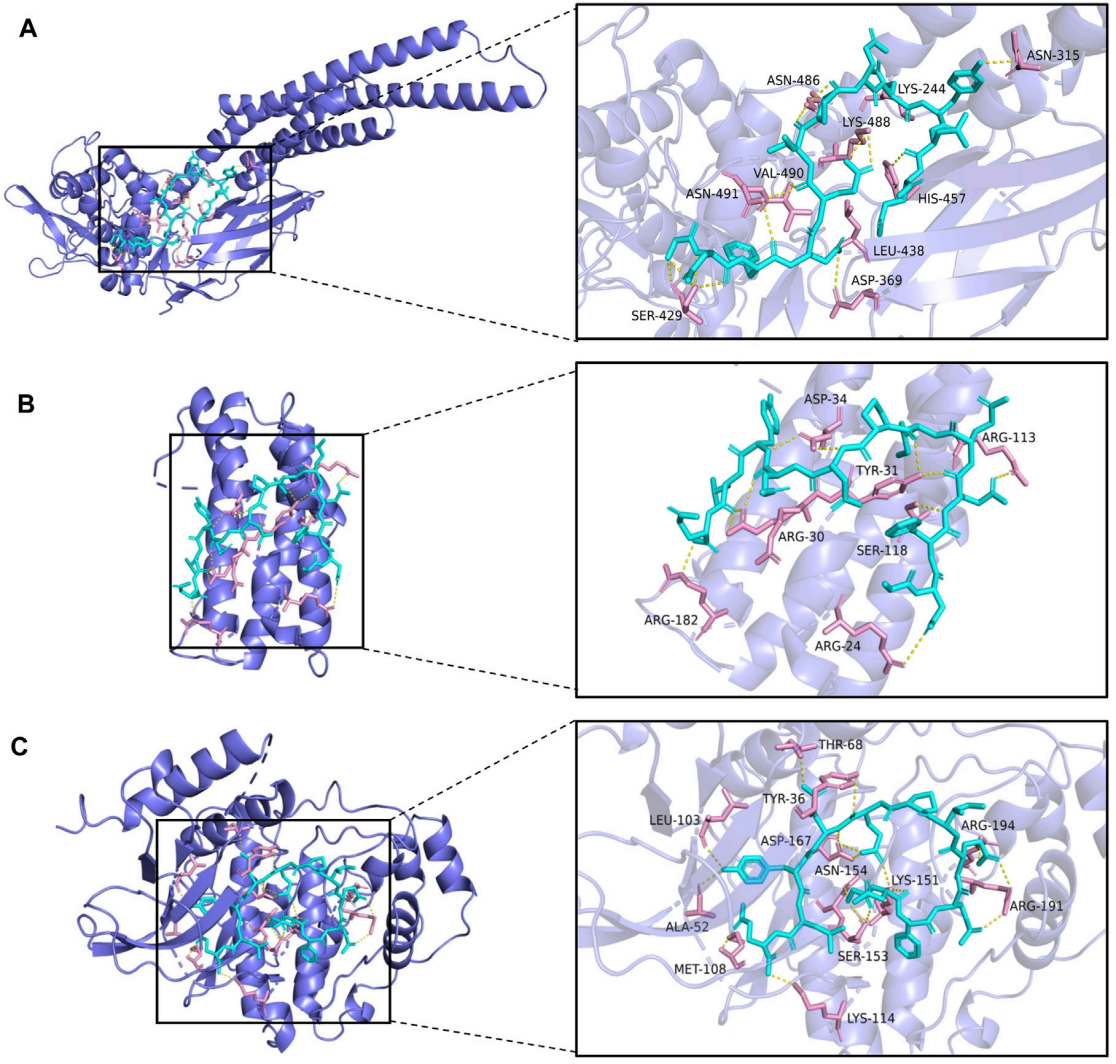
The top 3 binding energy molecular docking models. **(A)** Hirudin and STAT3. **(B)** Hirudin and IL-6. **(C)** Hirudin and MAPK1.

From the drug-target network, the gene targets were sorted by degree value, and the first 3 key targets (STAT3, IL-6, MAPK1) were selected for molecular docking with hirudin, respectively. The chemical structure of hirudin was downloaded from the PubChem database (PubChem CID. 72941487). It is generally believed that the binding energy of less than -7.0 kcal/mol indicates that the ligand has a strong binding activity to the receptor. Molecular docking results were shown in Table 2 and Supplementary Table S6. The results showed that the binding energy between hirudin and the key target was less than −7.0 kcal/mol, indicating that the binding activity between the two was good. Next, Pymol 2.3.2 software was used for the visual analysis of molecular docking. The images showed that hirudin formed hydrogen bonds with ASN-315, ASN-486, LYS-244, LYS-488, VAL-490, ASN-491, HIS-457, LEU-438, ASP-369, and SER-429 amino acids near the active site to bind to STAT3. It forms hydrogen bonds with asp-34, ARG-113, TYR-31, ARG-30, SER-118, ARG-182, and ARG-24 to bind to IL-6. It forms hydrogen bonds with 12 amino acids including THR-68, TYR-36, LEU-103, ARG-194, ASP-167, ASN-154, LYS-151V, ARG-191, ALA-52, MeT-108, SER-153, and LYS-114 to bind to MAPK1. Therefore, hirudin showed good activity in binding to the target and may be an inhibitor of these three targets. The corresponding binding sites were shown in Figure 5.

**TABLE 2:** Molecular docking affinity analyzed by vina.

Receptor	Ligand	Affinity		
		Minimum	Average of the top 5 minimums	Average of all minimum values
STAT3	Hirudin	−9.4	−9	−8.84444
IL-6		−9.7	−9.08	−8.86667
MAPK1		−11.6	−11.28	−11.03333

### Hirudin Inhibited AngII-Induced Death of H9c2 Cells

**FIGURE 6:**
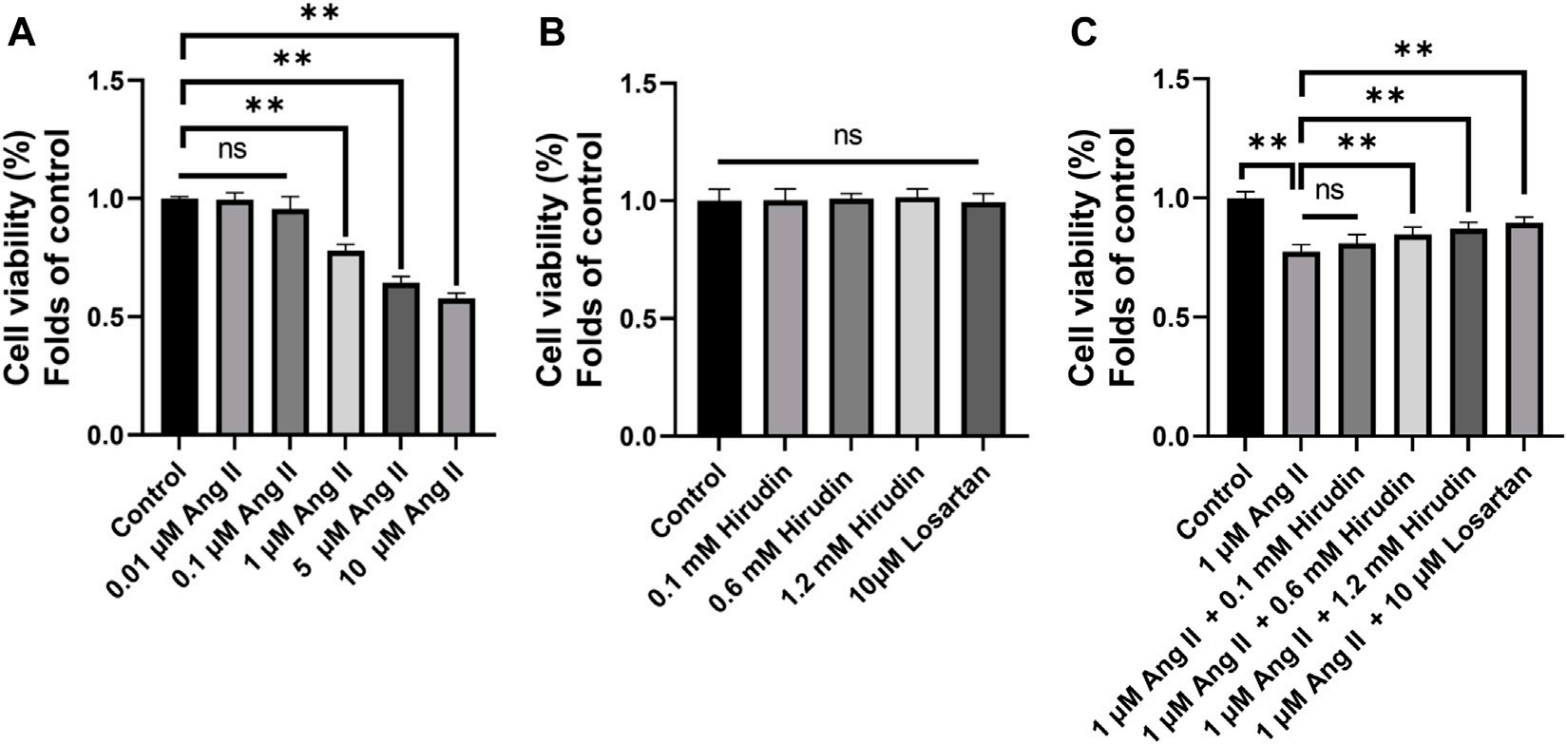
**(A)** H9c2 cells were treated with (0.01, 0.1, 1, and 10 μM) AngII for 24 h, respectively. **(B)** H9c2 cells were treated with (0.1, 0.6 and 1.2 mM) hirudin and 10 μM losartan for 24 h **(C)** H9c2 cells were treated with 1 μM AngII and (0.1, 0.6 and 1.2 mM) hirudin and 10 μM losartan for 24 h. Bars represent the mean ± SD, *n* = 5 **p < 0.01.

CCK-8 method was used to screen the optimal concentrations of AngII and hirudin. As shown in Figure 6A, H9c2 cells pretreated with 0.01 and 0.1 μM AngII for 24 h had no significant effect on cell viability, while concentrations of 1 and 10 μM AngII significantly decreased cell viability. Therefore, the concentration of 1 μM AngII was selected for subsequent experiments. Then, as shown in Figure 6B, H9c2 cells were incubated with hirudin at concentrations of 0.1, 0.6 and 1.2 mM, and 10 μM losartan for 24 h. As expected, neither hirudin nor losartan had a significant effect on cell viability, indicating that hirudin and losartan at these concentrations were not significantly toxic to H9c2 cells. Finally, in order to determine the optimal dose of drug therapy, H9c2 cells were cultured with 1 μM AngII, 0.1, 0.6, and 1.2 mM hirudin and 10 μM losartan for 24 h. As shown in Figures 6C 1 μM AngII significantly reduced cell viability, while 0.6 and 1.2 mM hirudin and 10 μM losartan increased cell viability.

### Hirudin Inhibits AngII-Induced Hypertrophy of H9c2 Cells

**FIGURE 7:**
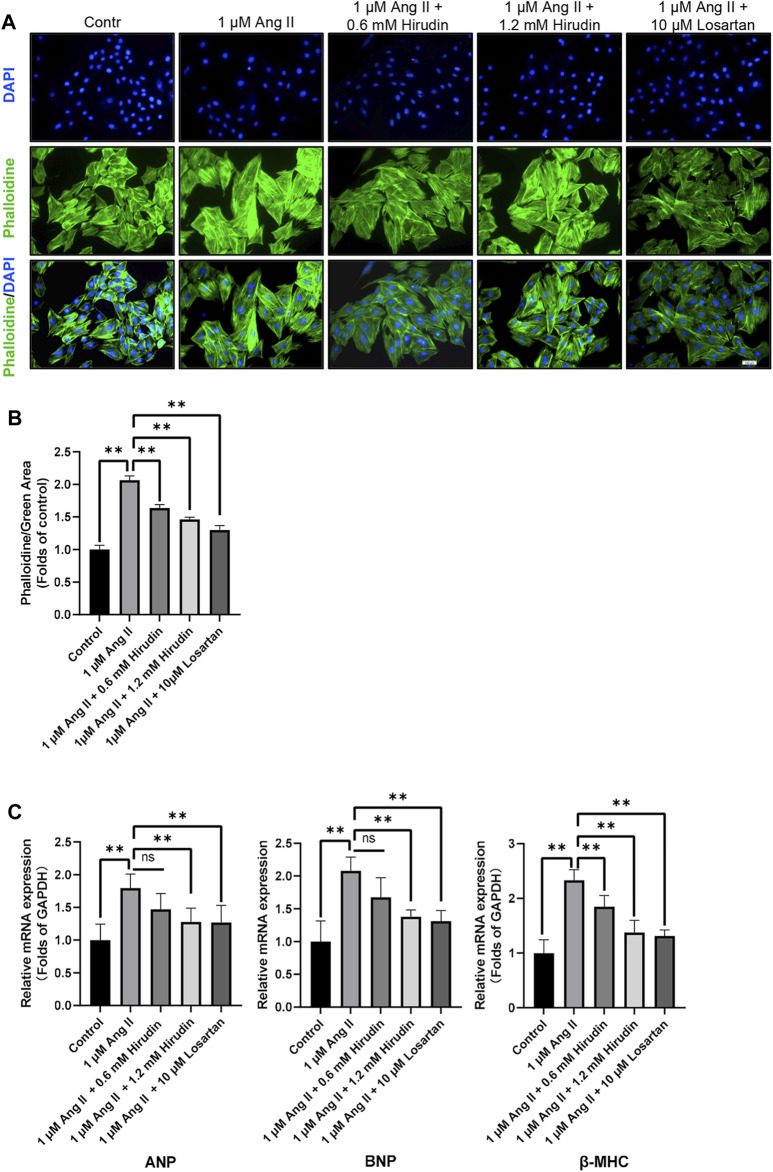
**(A)** H9c2 cells were treated with 1 μM AngII, (0.6, 1.2 mM) hirudin and 10 μM losartan for 24 h, respectively, and then labeled with phalloidin and DAPI, and observed by fluorescence microscope, magnification: × 200. **(B)** Green area for analysis of phalloidin. Bars represent the mean ± SD, *n* = 3. **(C)** The expression of ANP, BNP, and β-MHC was analyzed by qRT-PCR, *n* = 6 **p < 0.01.

Phalloidin staining and detection of cell hypertrophy genes were used to observe the effect of hirudin on AngII-induced hypertrophy of H9c2 cells. Cells were processed as described above and then labeled with phalloidin and DAPI. The data indicated that 1 μM AngII expanded the green area, however, both hirudin and losartan significantly reduced the green area at different concentrations, which also showed some dose-dependence (Figure 7). Then, the mRNA expressions of ANP, BNP and β-MHC as target genes of cell hypertrophy were significantly increased after 1 μM AngII treated, and it should be noted that hirudin and losartan at different concentrations significantly reduced their expression (Figure 7C), suggesting that hirudin can inhibit hypertrophy of H9c2 cells by inhibiting the expression of these related genes.

### Hirudin Inhibits AngII-Induced Hypertrophy of H9c2 Cells By Down-Regulating Key Target Genes and Proteins

**FIGURE 8:**
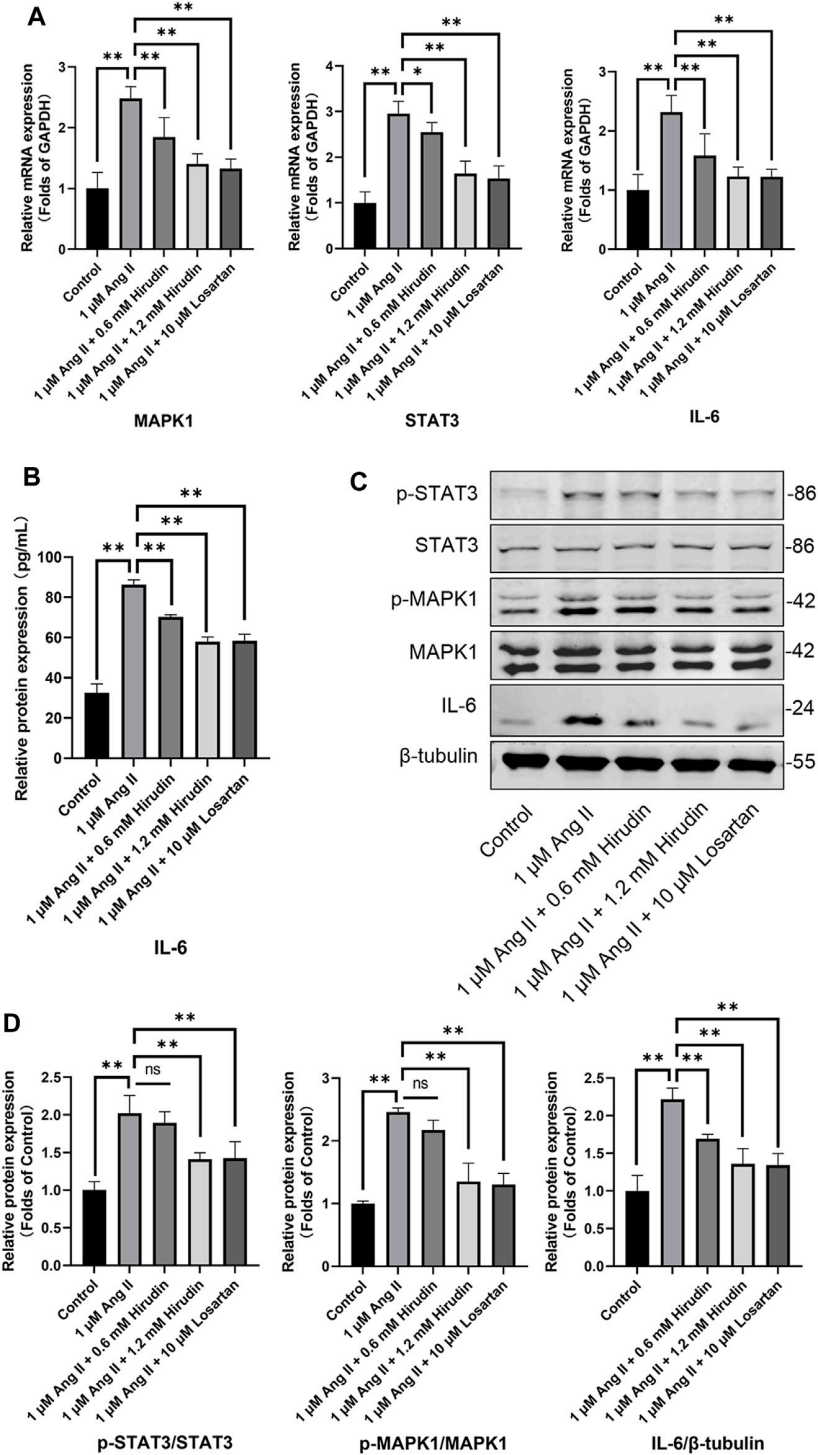
H9c2 cells were treated with 1 μM AngII, (0.6, 1.2 mM) hirudin and 10 μM losartan for 24 h, respectively. **(A)** The expression of STAT3, MAPK1 and IL-6 were analyzed by qRT-PCR, *n* = 6. **(B)** The IL-6 in cell supernatant was assayed by ELISA, *n* = 5. **(C)** Western blot for the expression of proteins including STAT3, p-STAT3, MAPK1, p-MAPK1 and IL-6. **(D)** Quantified by western blot analysis and normalized to control, *n* = 3. Bars represent the mean ± SD, *p < 0.05, **p < 0.01.

**FIGURE 9:**
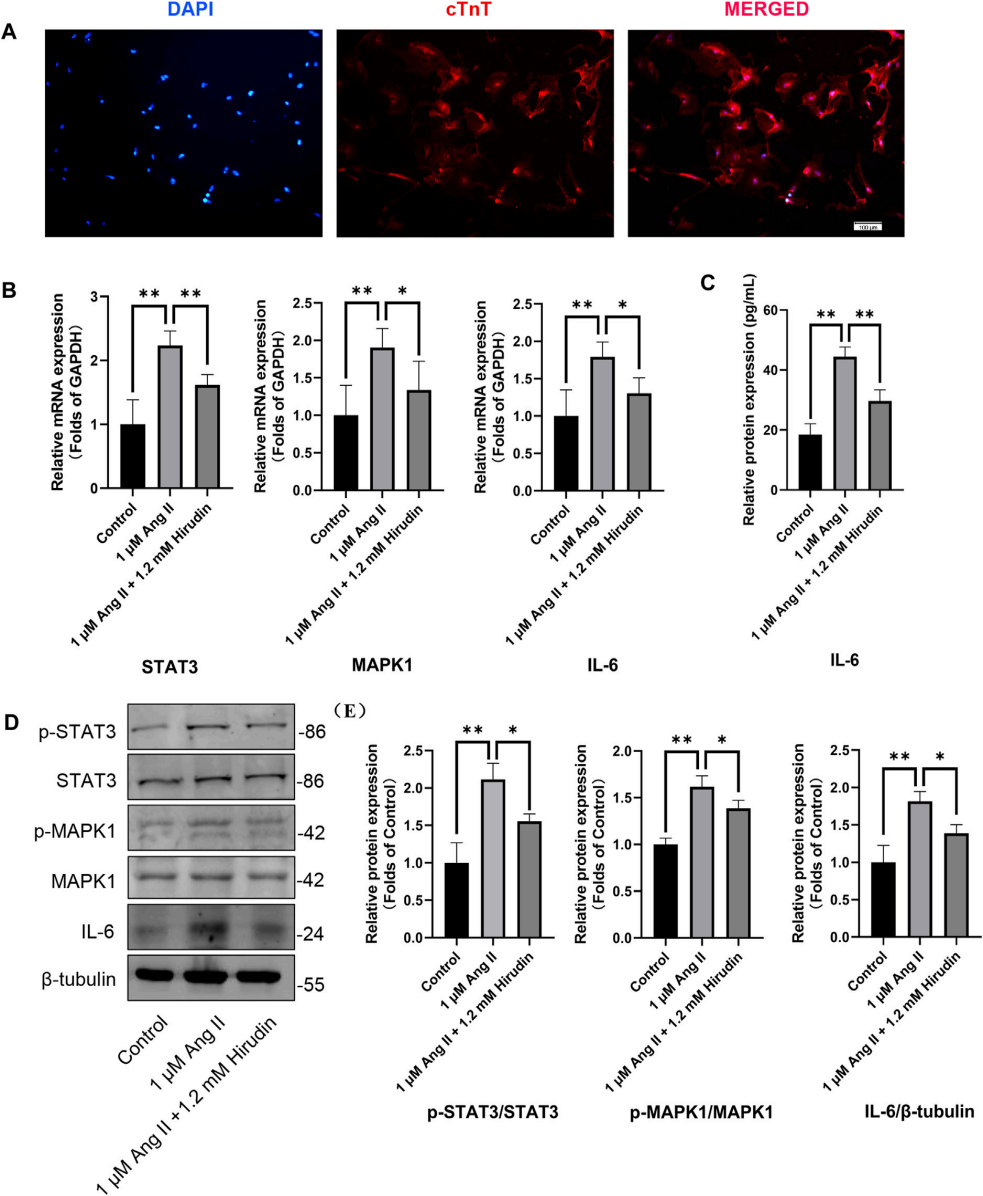
Neonatal rat cardiomyocytes were treated with 1 μM AngII and 1.2 mM hirudin for 24 h. **(A)** Immunofluorescence staining (cTnT) identified the obtained cells were cardiomyocytes, and the red staining area was troponin specifically expressed by cardiomyocytes. **(B)** The expression of STAT3, MAPK1 and IL-6 were analyzed by qRT-PCR, *n* = 6. **(C)** The IL-6 in cell supernatant was assayed by ELISA, *n* = 5. **(D)** Western blot for the expression of proteins including STAT3, p-STAT3, MAPK1, p-MAPK1 and IL-6. **(E)** Quantified by western blot analysis and normalized to control, *n* = 3. Bars represent the mean ± SD, *p < 0.05, **p < 0.01.

Our network pharmacology analysis revealed that STAT3, IL-6, and MAPK1 were the key target genes for hirudin therapy of cell hypertrophy. Therefore, we further assessed the expression levels of STAT3, IL-6, and MAPK1 and their phosphorylated counterparts by qRT-PCR and western blot, and the results were shown in Figure 8. Stimulation of H9c2 cells with 1 μM AngII significantly increased the mRNA expressions of STAT3, MAPK1, and IL-6, as well as the phosphorylation levels of STAT3 and MAPK1 and the expression of the inflammatory factor IL-6 protein. However, the above target mRNA expression, protein phosphorylation, and inflammatory factor expression were significantly inhibited by 0.6 and 1.2 mM hirudin and 10 μM losartan. More importantly, experiments based on neonatal rat cardiomyocytes were used for further validation (Figure 9). After primary cardiomyocytes were extracted as we previously described, red fluorescence (cTnT)-labeled cardiomyocytes were identified by immunofluorescence, and after 7 days of culture, the cardiomyocytes can observe obvious spontaneous beating under the microscope. After stimulation with 1 μM AngII for 24 h, the mRNA expressions of STAT3, MAPK1, and IL-6 in cardiomyocytes were significantly up-regulated, while 1.2 mM hirudin inhibited the expression of the above mRNAs. The determination of protein expressio for IL6 in cell supernatant by ELISA was also increased in the model of cardiac hypertrophy, and was significantly inhibited by hirudin treatment, while the phosphorylation of STAT3 and MAPK1 was also significantly inhibited.

## Discussion

At present, hirudin has been extensively used in clinic, including a variety of formulations prepared by natural hirudin and recombinant hirudin. As a traditional Chinese medicine, leech has been widely used in ancient China, but we still know little about its pharmacological mechanism, especially for cardiovascular diseases that have not been systematically studied.

Myocardial hypertrophy is a complex disease with a step-by-step sequence of events ranging from cellular hypertrophy, fibrosis to necrosis, which are associated with multiple proteins or pathways during development and progression (Oka et al., 2014). It is worth noting that studies have shown that hirudin improves myocardial infarction by inhibiting oxidation (Zhang H. et al., 2020), and can also protect AngII-induced myocardial fibroblast fibrosis by inhibiting extracellular signal-regulated kinase (Yu et al., 2018). Cell hypertrophy and fibrosis are an important pathophysiological process in myocardial remodeling, and it is not difficult to speculate that hirudin also plays an important role in the treatment of cardiac hypertrophy, which may be beneficial for the treatment of cardiac hypertrophy due to its extensive pharmacological activities (Junren et al., 2021), but on the other hand, the property of polypeptides may make it difficult to study the underlying mechanisms in depth. The network pharmacology approach integrating systems biology and computer technology can provide directions for the mechanistic study of complex botanicals or compounds (Nowak, 2002). Therefore, we used this method to elucidate the pharmacological mechanism of hirudin in the treatment of myocardial hypertrophy.

Tracing the medical value of hirudin, the classic Chinese medicine book *Shen Nong’s Herbal Classic* (25–220 AD) first recorded that leech was used to treat female amenorrhea, and then gradually developed to be used for various diseases with congestion (Dong et al., 2016). In the development of modern medicine, Haycraft first discovered the presence of an anticoagulant substance in the extract of medical leech in 1884, which was later named hirudin in 1904 by Jacoby. Markwardt in 1955, successfully isolated pure hirudin from the peripharyngeal glands of the medical leech. Harvey isolated the pure line cDNA encoding hirudin in 1986, and recently the use of genetic engineering technology to prepare recombinant hirudin has become a reality (Fields, 1991; Junren et al., 2021). Hirudin is a highly effective, specific, and strongest thrombin inhibitor currently known, with a complex structure and diverse pharmacological values. Therefore, it may have a wide range of effects in the treatment of cardiovascular diseases and has a great development value for clinical application. We searched current literature on hirudin and cardiovascular diseases, and found that there was no research on the use of hirudin to treat myocardial hypertrophy. Therefore, the value of this study is of pioneering significance.

ACEI and ARB drugs are often used as the first choice for the treatment of cell hypertrophy and cell fibrosis models established by AngII, as well as the animal models of heart failure caused by hypertension and overpressure load. In clinic, ACEI and ARB drugs are often used as first-line drugs for the treatment of hypertension and heart failure. ACEI can inhibit the transformation from relatively inactive AngI to AngII, and at the same time, tissue bradykinin is degraded, while AngII generated by bypass can not be inhibited. However, ARB can produce pharmacological effects similar to ACEI by selectively blocking AngII receptor, inhibiting its vasoconstriction, raising blood pressure and promoting aldosterone secretion. In the selection of positive control drugs in this study, losartan, as a representative ARB drug, directly reflects the degree of its AngII antagonism, and forms a more objective comparison with the therapeutic effect of hirudin.

From the perspective of therapeutic target prediction and pathway analysis, hirudin may play its role in inhibiting myocardial hypertrophy by regulating the PI3K/AKT signaling pathway and key targets such as STAT3, IL-6, and MAPK1. Studies have shown that AngII can cause damage to the PI3K/AKT/eNOS signaling pathway, resulting in myocardial hypertrophy (Gu et al., 2014; Zhan et al., 2021). The activation of PI3K promotes the phosphorylation of its downstream Akt and participates in signal transduction. In the cardiovascular system, PI3K/AKT signaling pathway plays an important role in regulating angiogenesis, cardiomyocyte apoptosis, and metabolism, these physiological processes and functions were closely related to heart failure (Cheng et al., 2021; Qin et al., 2021). JAK/STAT signaling pathway plays an extremely important role in the occurrence and development of myocardial hypertrophy (Al-Rasheed et al., 2015; Aboulhoda, 2017; Zhang J. et al., 2020). When cardiac myocytes undergo hypertrophy and fibrosis due to external stimulation, activated JAK can activate STAT3 by tyrosine phosphorylation, enter the nucleus from the cytoplasm, up-regulate hypertrophy related genes including ANP and BNP, and induce hypertrophy response (Kurdi et al., 2018; Ye et al., 2020, 3; Baldini et al., 2021). The MAPK/ERK signaling pathway is an important signaling system that mediates cell proliferation, cell differentiation, cell cycle, and apoptosis (Gallo et al., 2019). Inhibiting the phosphorylation of MAPK1 can reduce cardiomyocyte apoptosis and slow down the progression of myocardial hypertrophy (Huang et al., 2018; Ruan et al., 2021). Inflammation and myocardial hypertrophy interact with each other. Studies have shown that under the stimulation of inflammation, stromal cells secrete a large number of innate immune signaling mediators such as IL-6, IL-1β, etc. (Carrillo-Salinas et al., 2019; Lafuse et al., 2020), and the release of inflammatory factors, in turn, promotes the phosphorylation of STAT3 and MAPK1 in myocardial tissue, which further aggravates the inflammatory response (Mir et al., 2012; Jin et al., 2018; Huo et al., 2021).

Although this study has been partially validated, there are still some limitations: First, *in vitro* study cannot confirm the therapeutic effect of hirudin on myocardial hypertrophy *in vivo*, although it has been reported that hirudin can improve cardiac function and myocardial fibrosis, which to a certain extent supports the innovative viewpoints proposed in this study. However, it still cannot fully make up for the incompleteness of the data caused by the lack of *in vivo* experiments. Second, the H9c2 cardiomyocyte cell line was used in the main experiment of this study, and the primary cardiomyocytes were used to replicate and validate part of the results, which explains the pharmacological effects of hirudin in treating cell hypertrophy and inhibiting the expression of key proteins, but this validation is incomplete. Third, all databases are dynamically updated, which may affect some of the conclusions we draw. In future research, we will continue to reveal the mechanism of action of hirudin in the treatment of cardiovascular diseases through *in vitro* and *in vivo* experiments.

In short, there is a certain research basis for the targeted therapy of the above pathways and targets. When we verified the results of network pharmacology, it was found that hirudin contains a complex polypeptide structure and exhibits a good binding ability to STAT3, IL-6, and MAPK1 at multiple sites. Through *in vitro* experiments, we found that AngII-induced cell hypertrophy and death were rescued by hirudin. Mechanistically, the effect of hirudin on inhibiting cell hypertrophy and protecting cell death may be related to the inhibition of the mRNA and protein expressions of STAT3, MAPK1, and IL-6. In future studies, we will further explore the detailed pharmacological mechanism of hirudin in improving myocardial hypertrophy.

## Conclusion

In summary, combined with network pharmacology prediction, molecular docking, and *in vitro* experimental verification, we studied the pharmacological mechanism of hirudin inhibiting myocardial hypertrophy. We have demonstrated that hirudin may inhibit cardiomyocyte hypertrophy mainly by regulating key targets such as STAT3, IL-6 and MAPK1. Our study further suggests that combining network pharmacological predictions and experimental validation studies may provide useful tools for detailing the mechanism of action of drugs. The potential therapeutic effects of hirudin on myocardial hypertrophy may be beneficial in patients with cardiovascular disease and warrant further investigation.

## Data Availability

The original contributions presented in the study are included in the article/Supplementary Materials, further inquiries can be directed to the corresponding authors.

## Abbreviations

ACEI, angiotensin-converting enzyme inhibitor; AngII, angiotensin II; ANP, atrial natriuretic peptide; ARB, angiotensin receptor blocker; BNP, brain natriuretic peptide; BP, biological process; BSA, bovine serum albumin; CC, cellular component; CCK-8, cell counting kit-8; GAPDH, glyceraldehyde-3-phosphate dehydrogenase; GO, gene ontology; IL-6, interleukin-6; KEGG, kyoto encyclopedia of genes and genomes; MAPK1, mitogen-activated protein kinase 1; MF, molecular function; NC membranes, nitrocellulose filter membrane; OB, oral bioavailability; RASS renin-angiotensin-aldosterone system; SDS-PAGE, sodium dodecyl sulfate-polyacrylamide gel electrophoresis; STAT3, signal transducer and activator of transcription 3; β-MHC, β-major histocompatibility complex.
