# Supplementary figures and images for: Network Pharmacology and In Vitro Experimental Verification Reveal the Mechanism of the Hirudin in Suppressing Myocardial Hypertrophy
Source: Front Pharmacol. 2022 Jun 15;13:914518. doi: 10.3389/fphar.2022.914518 (PMC9240481; doi:10.3389/fphar.2022.914518)

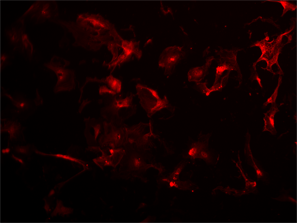

Supplement: Supplementary file 1 [file DataSheet1.zip › cTnT/CTNT.tif]

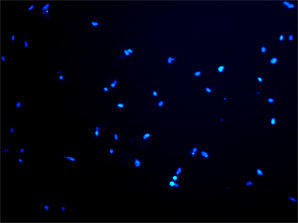

Supplement: Supplementary file 1 [file DataSheet1.zip › cTnT/DAPI.tif]

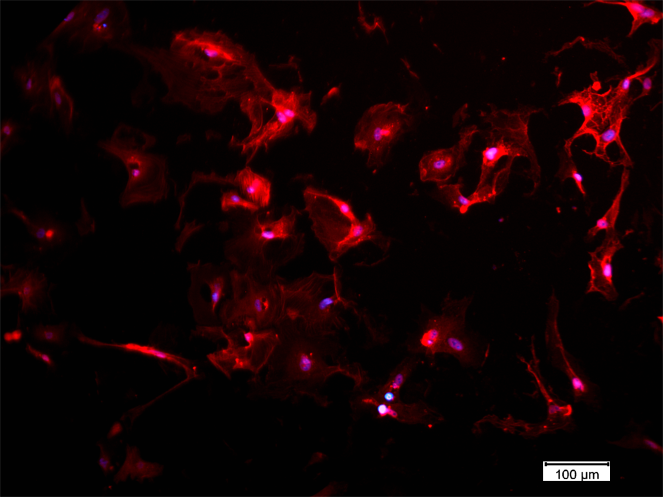

Supplement: Supplementary file 1 [file DataSheet1.zip › cTnT/MERGED1.tif]

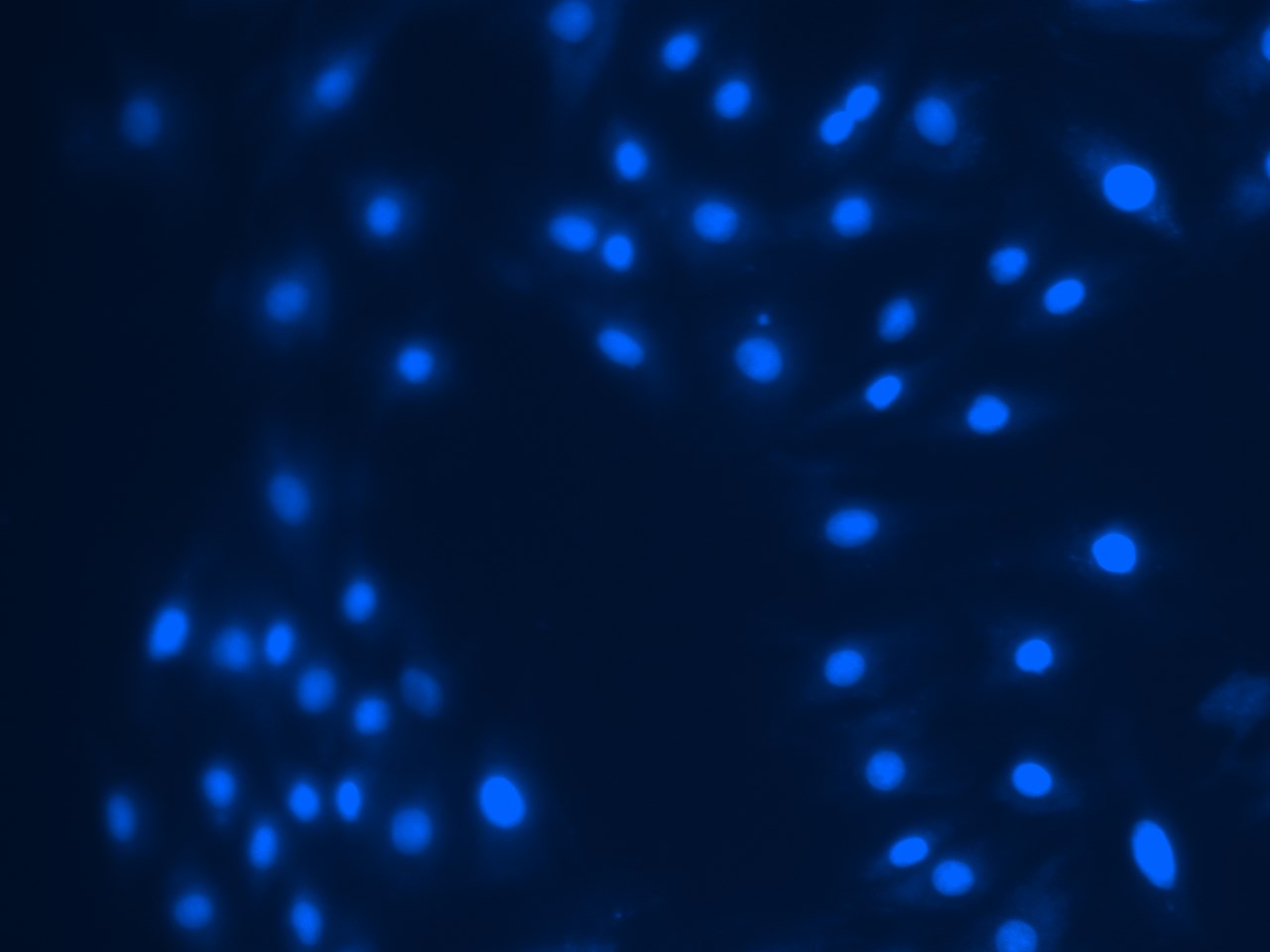

Supplement: Supplementary file 1 [file DataSheet1.zip › Pictures of Phalloidine/1 μM Ang II + 0.6 mM Hirudin/1.358/51.jpg]

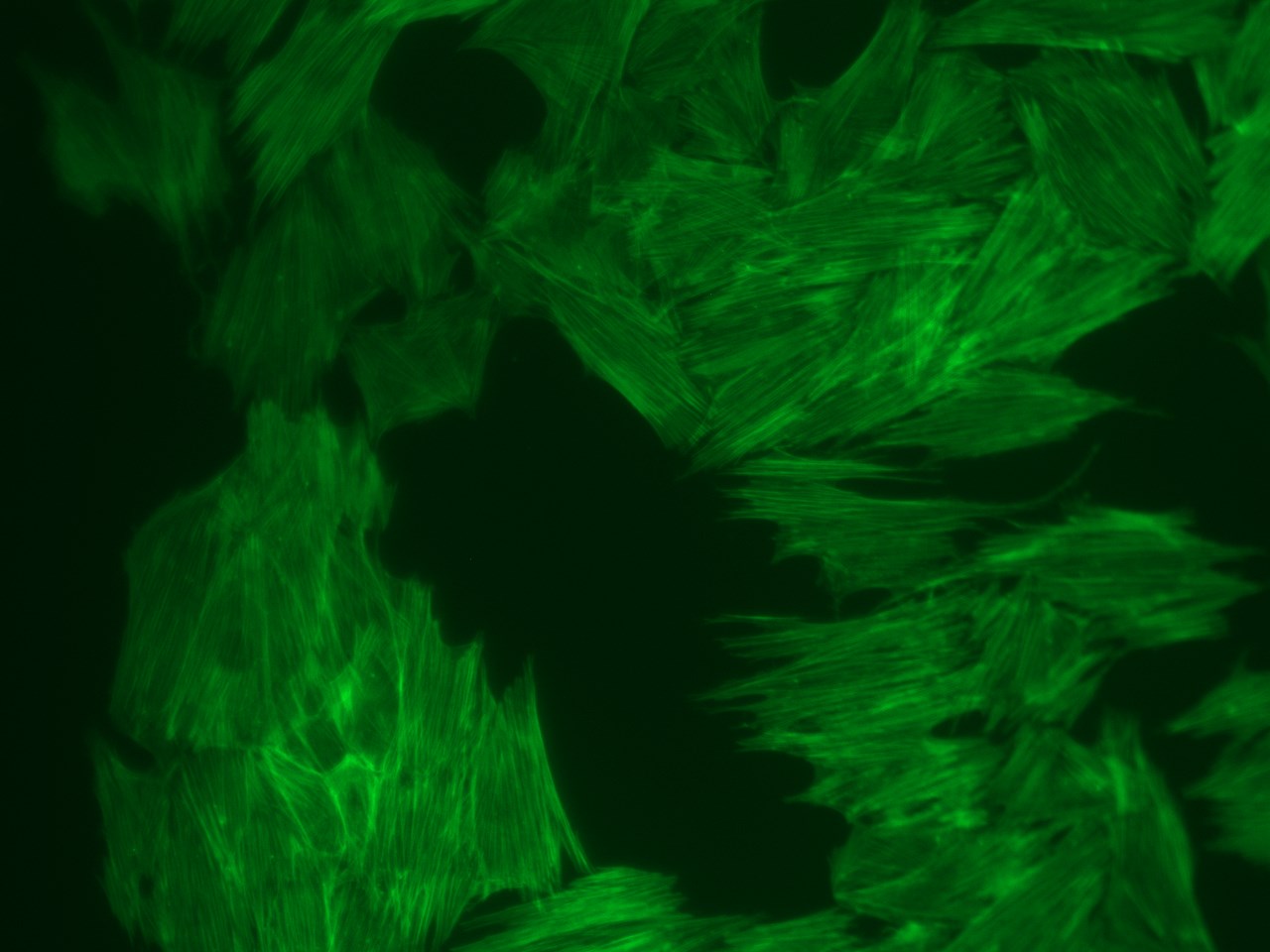

Supplement: Supplementary file 1 [file DataSheet1.zip › Pictures of Phalloidine/1 μM Ang II + 0.6 mM Hirudin/1.358/69.283.jpg]

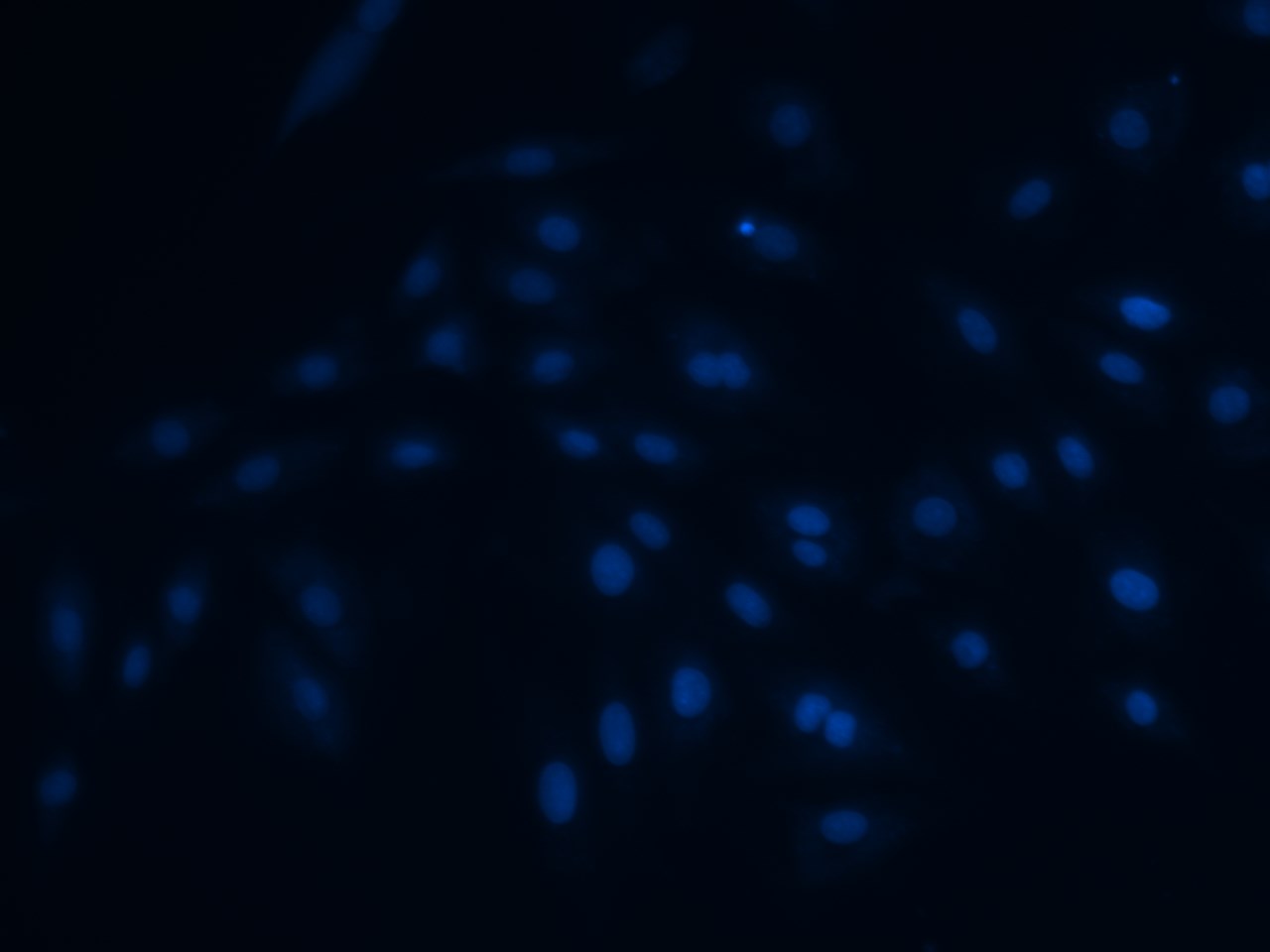

Supplement: Supplementary file 1 [file DataSheet1.zip › Pictures of Phalloidine/1 μM Ang II + 0.6 mM Hirudin/1.403/47.jpg]

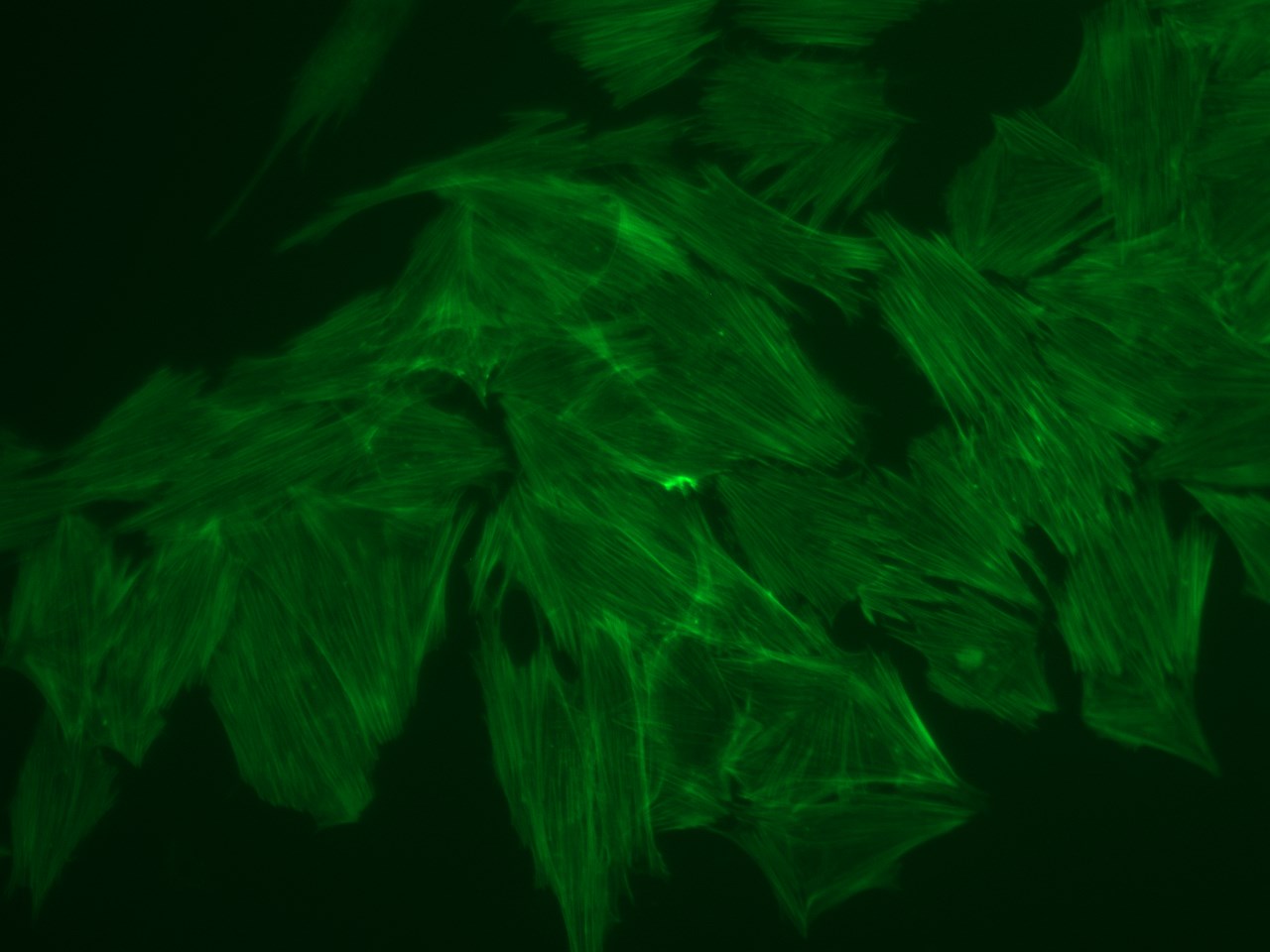

Supplement: Supplementary file 1 [file DataSheet1.zip › Pictures of Phalloidine/1 μM Ang II + 0.6 mM Hirudin/1.403/65.928.jpg]

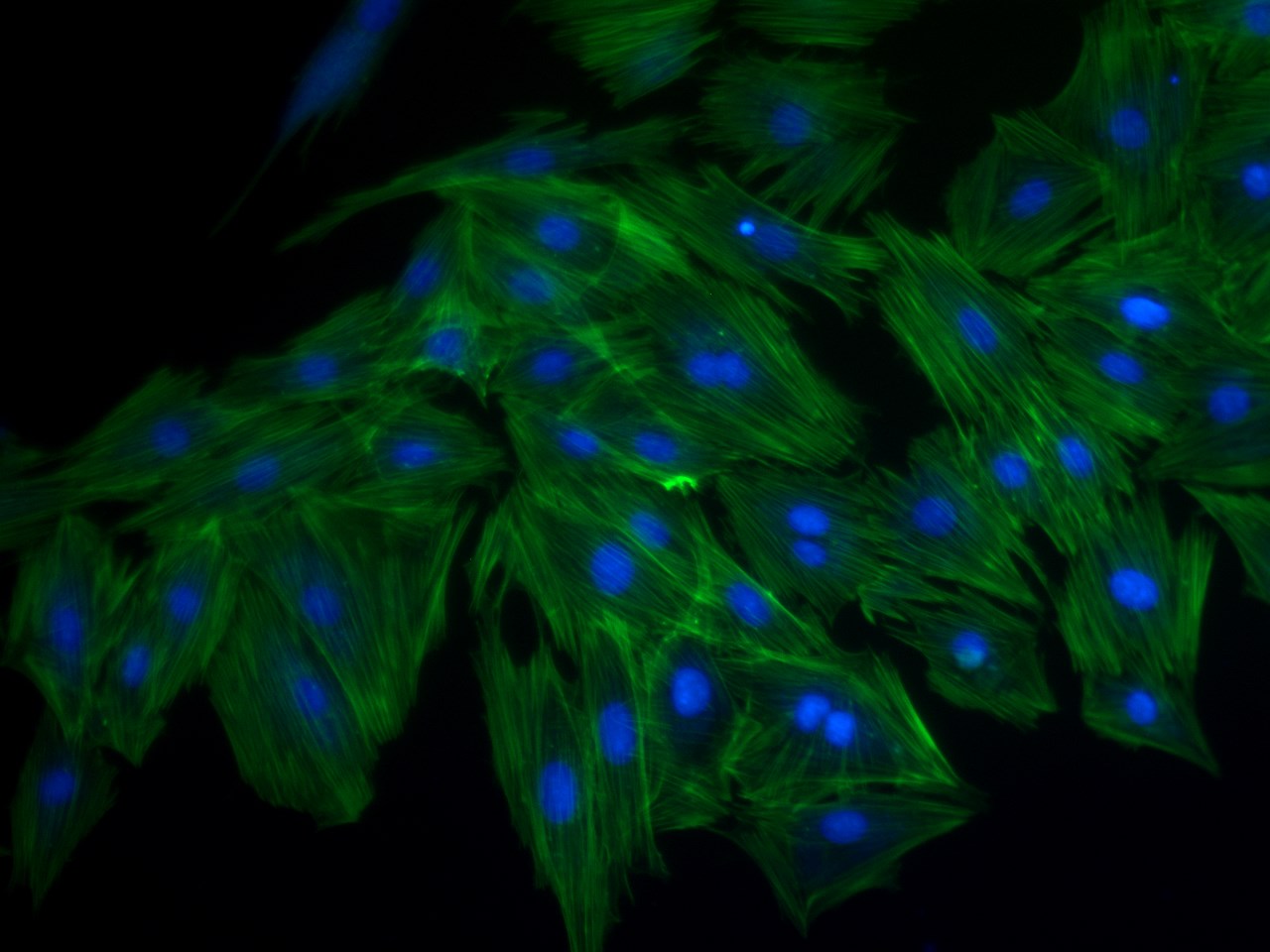

Supplement: Supplementary file 1 [file DataSheet1.zip › Pictures of Phalloidine/1 μM Ang II + 0.6 mM Hirudin/1.403/Merge.jpg]

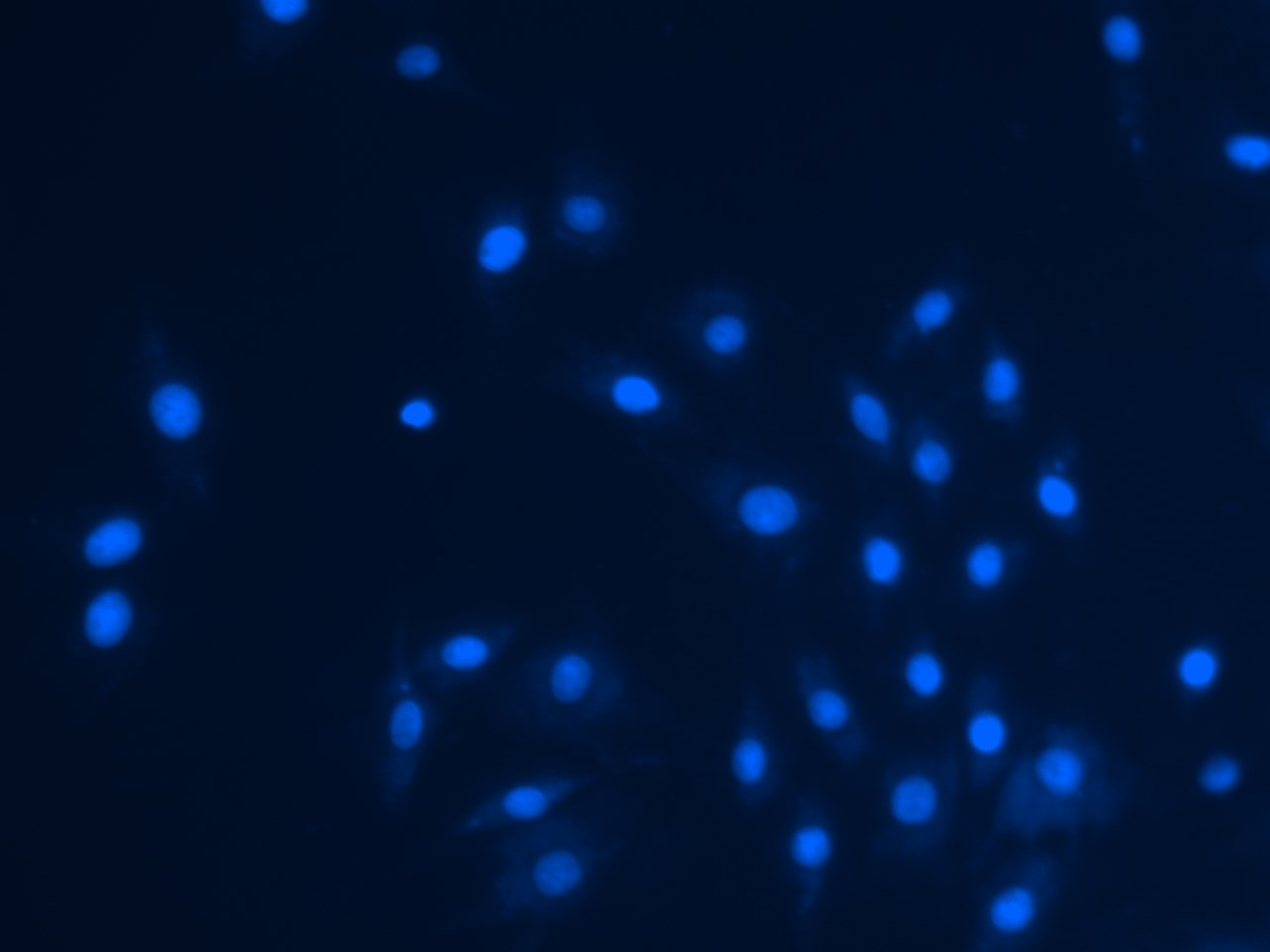

Supplement: Supplementary file 1 [file DataSheet1.zip › Pictures of Phalloidine/1 μM Ang II + 0.6 mM Hirudin/1.445/35.jpg]

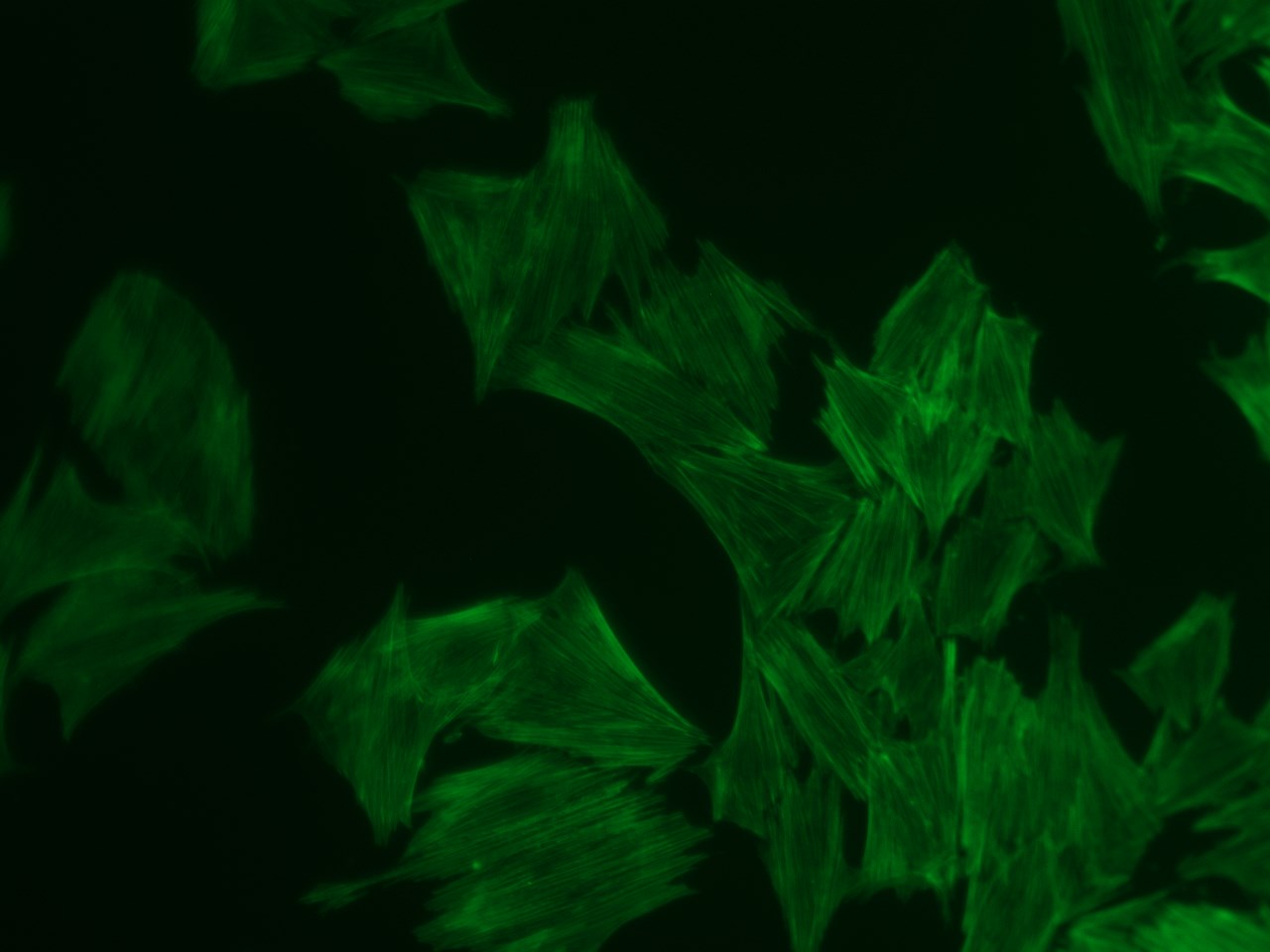

Supplement: Supplementary file 1 [file DataSheet1.zip › Pictures of Phalloidine/1 μM Ang II + 0.6 mM Hirudin/1.445/50.587.jpg]

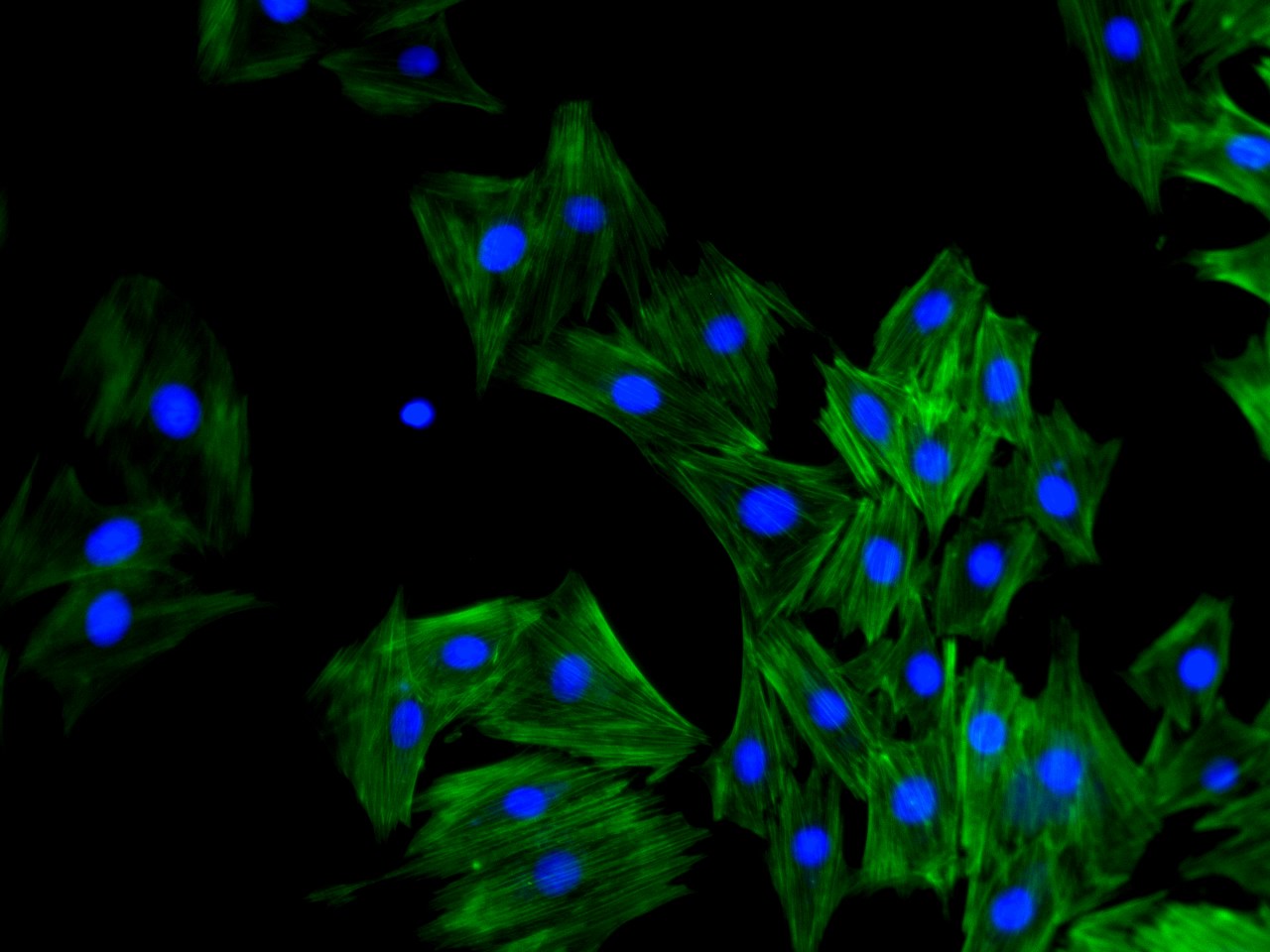

Supplement: Supplementary file 1 [file DataSheet1.zip › Pictures of Phalloidine/1 μM Ang II + 0.6 mM Hirudin/1.445/Merge.jpg]

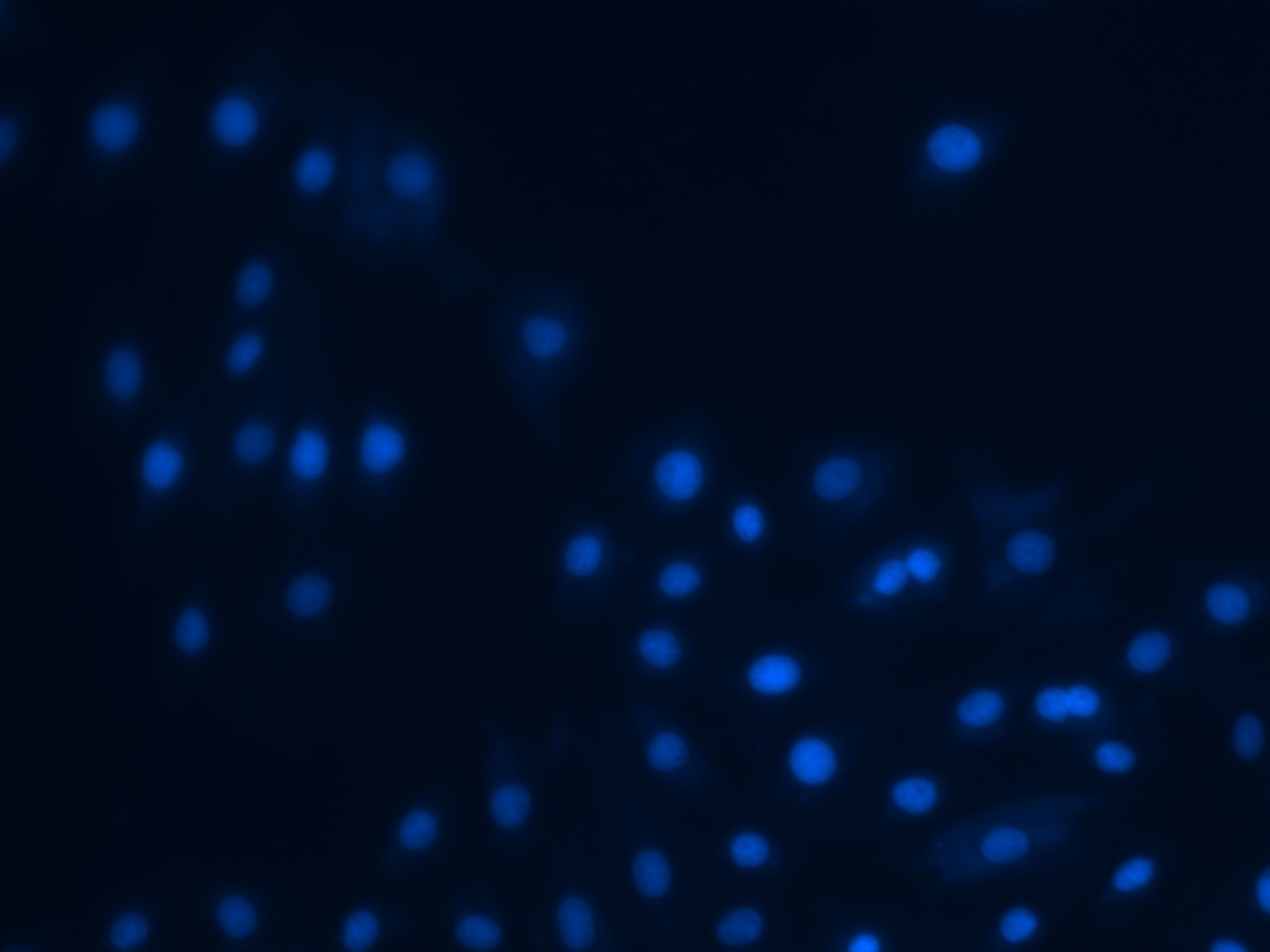

Supplement: Supplementary file 1 [file DataSheet1.zip › Pictures of Phalloidine/1 μM Ang II + 1.2 mM Hirudin/1.224/51.jpg]

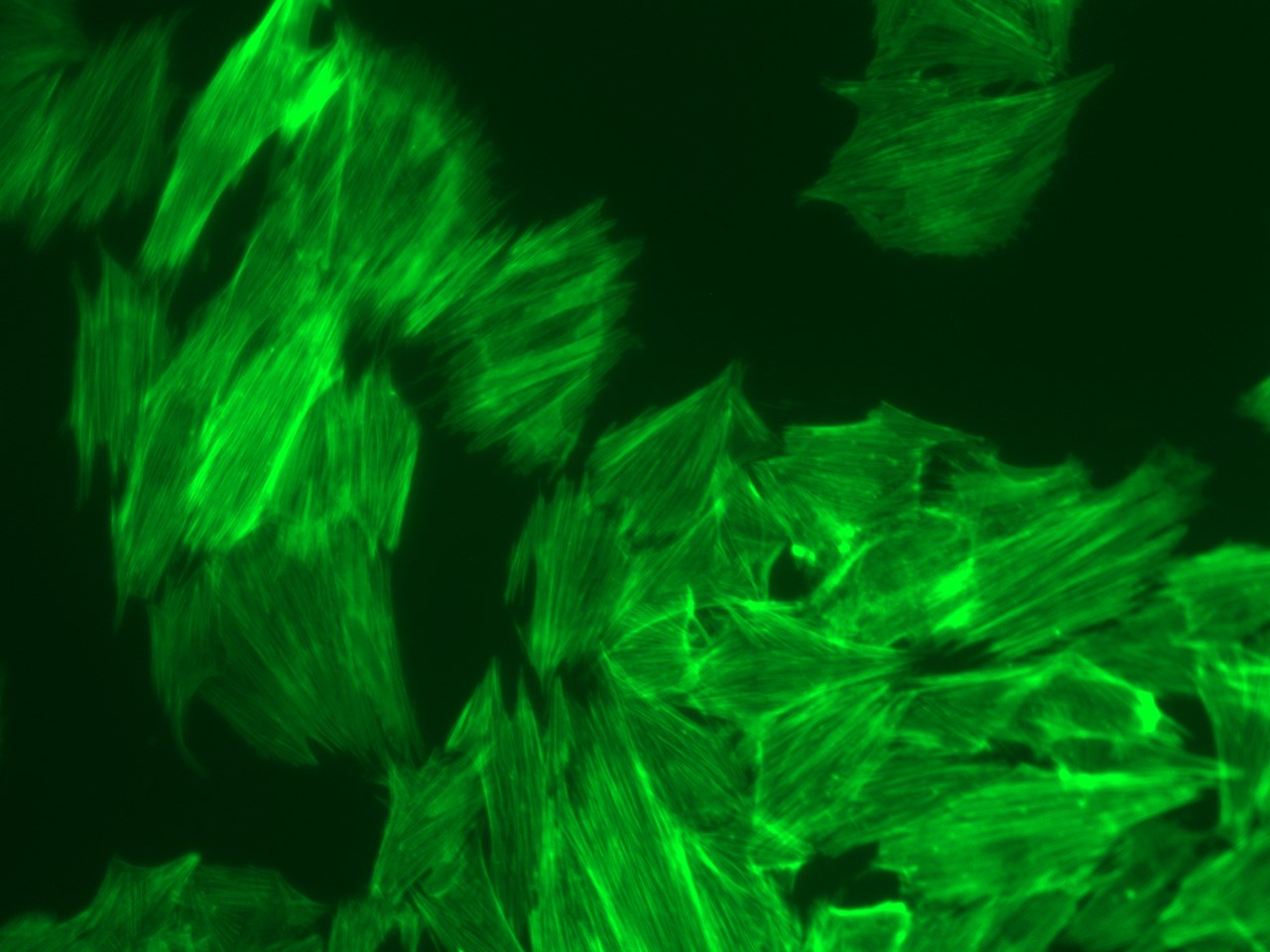

Supplement: Supplementary file 1 [file DataSheet1.zip › Pictures of Phalloidine/1 μM Ang II + 1.2 mM Hirudin/1.224/62.447.jpg]

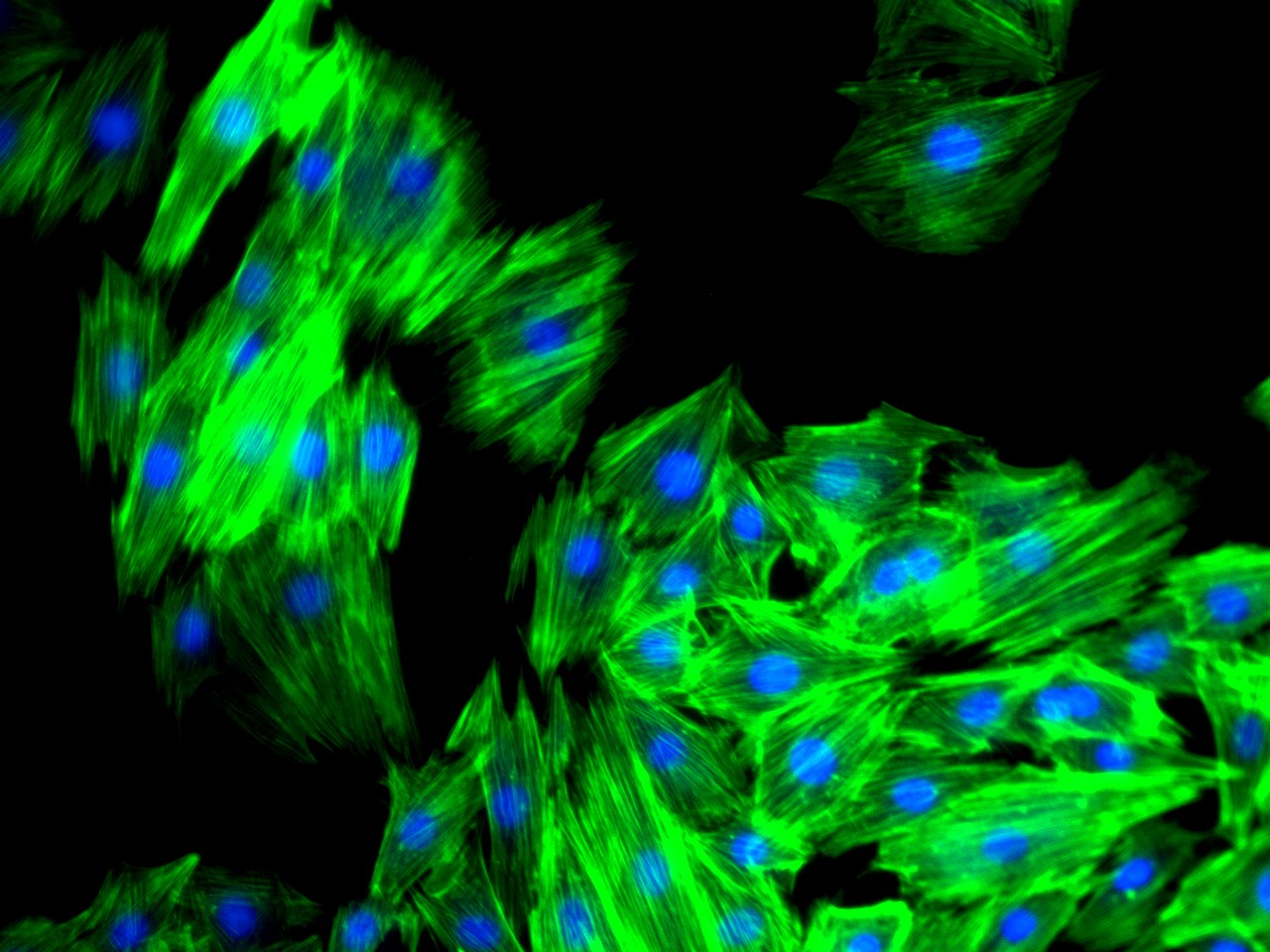

Supplement: Supplementary file 1 [file DataSheet1.zip › Pictures of Phalloidine/1 μM Ang II + 1.2 mM Hirudin/1.224/Merge.jpg]

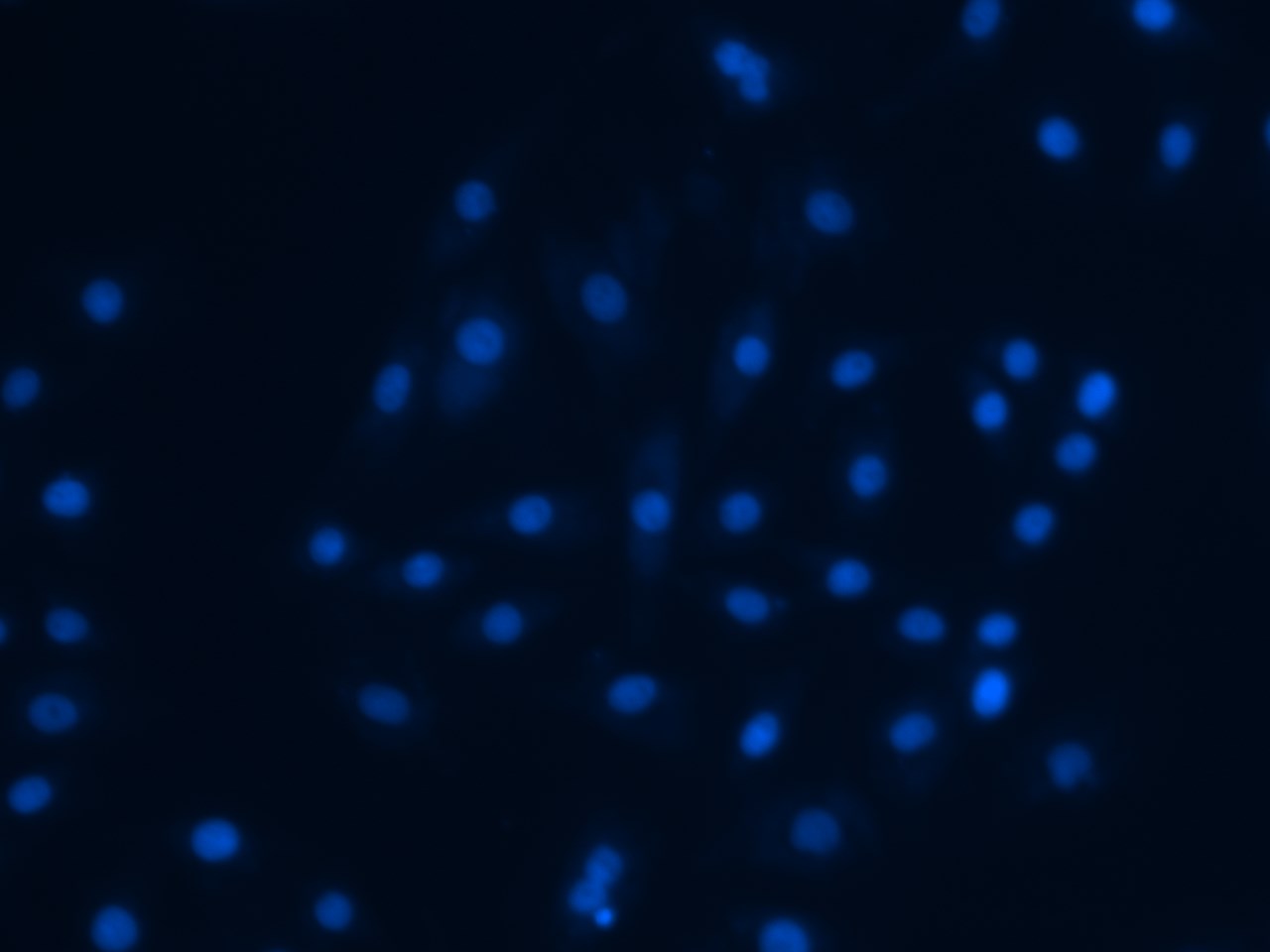

Supplement: Supplementary file 1 [file DataSheet1.zip › Pictures of Phalloidine/1 μM Ang II + 1.2 mM Hirudin/1.257/49.jpg]

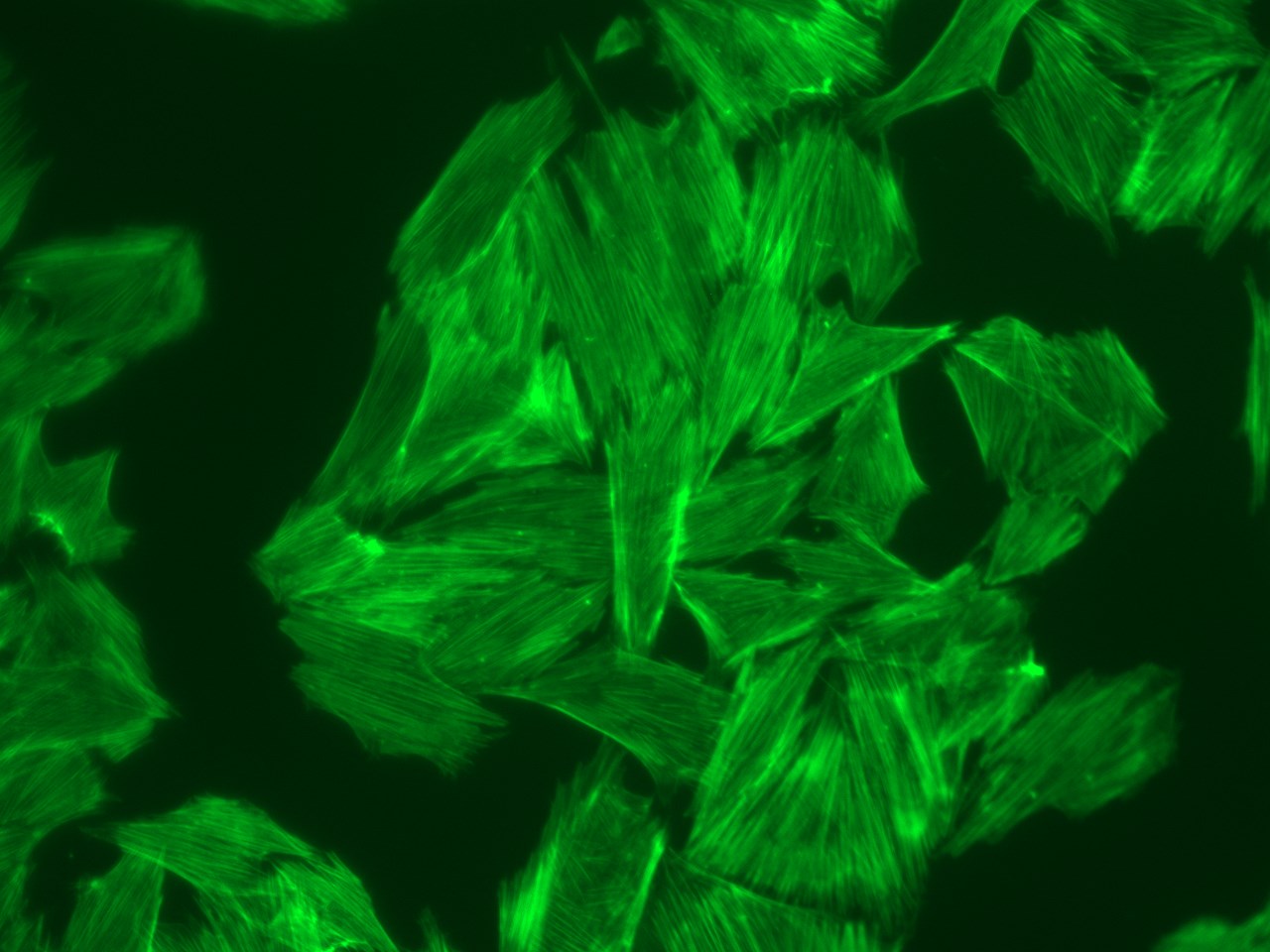

Supplement: Supplementary file 1 [file DataSheet1.zip › Pictures of Phalloidine/1 μM Ang II + 1.2 mM Hirudin/1.257/61.615.jpg]

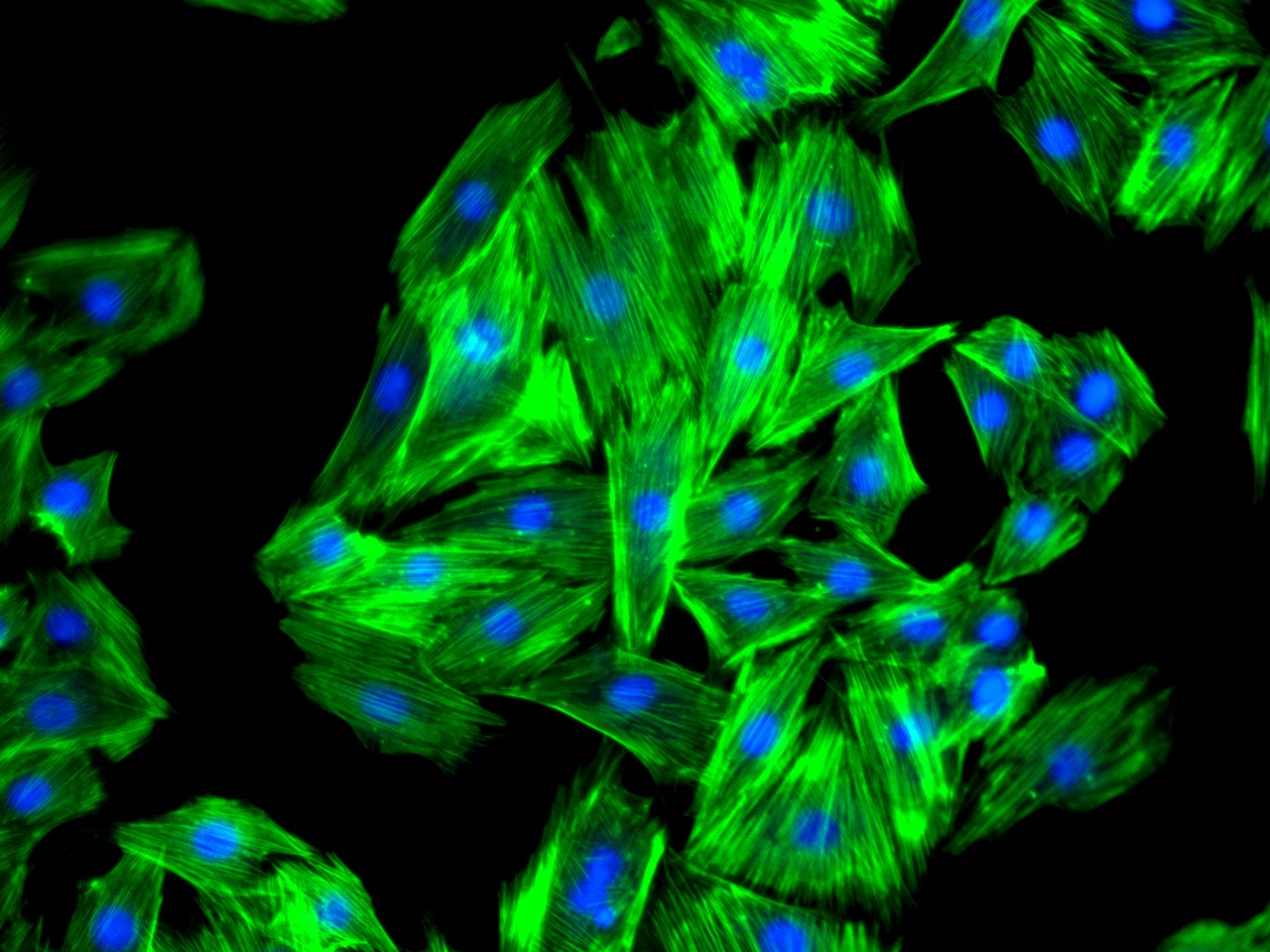

Supplement: Supplementary file 1 [file DataSheet1.zip › Pictures of Phalloidine/1 μM Ang II + 1.2 mM Hirudin/1.257/Merge.jpg]

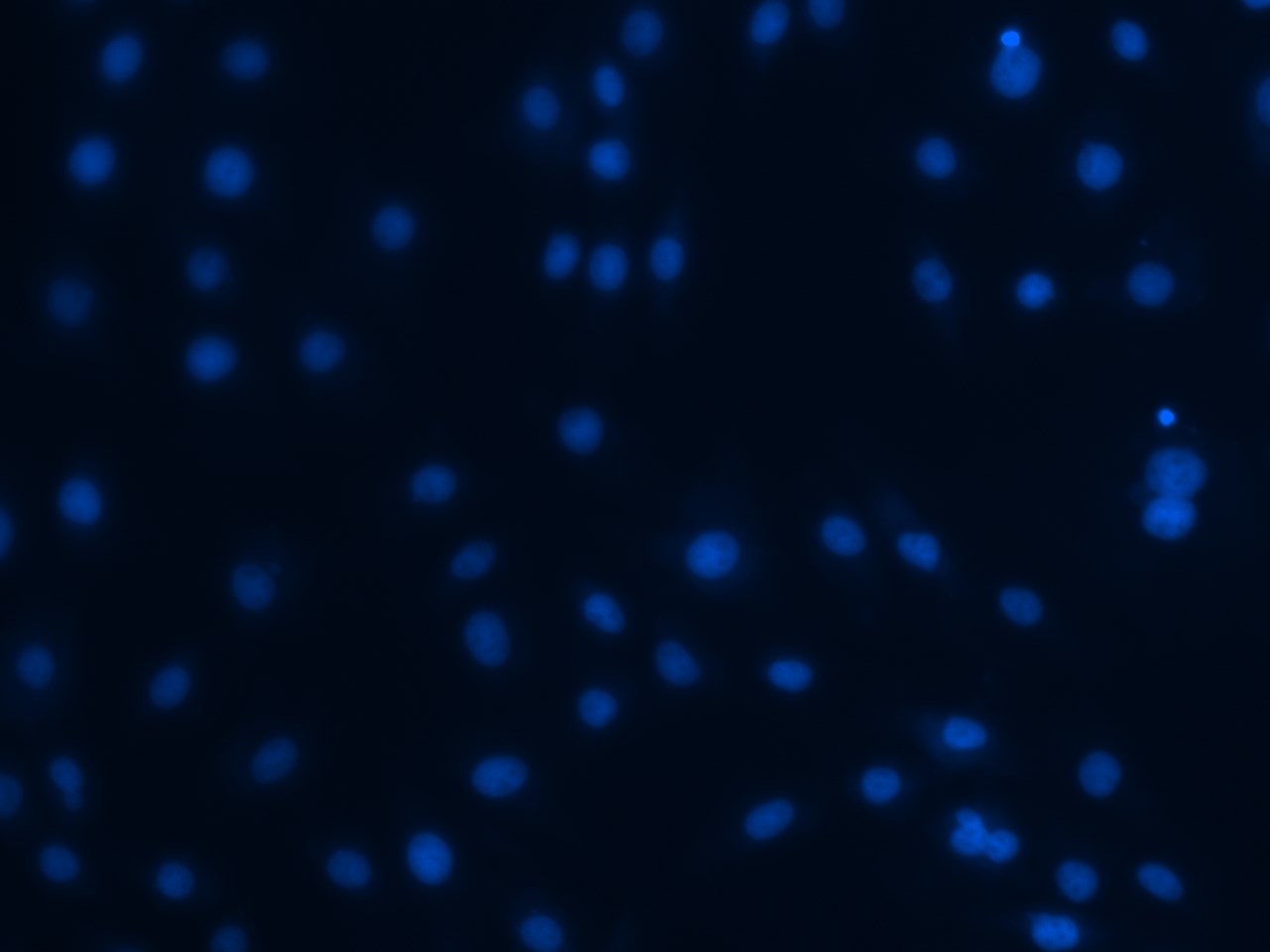

Supplement: Supplementary file 1 [file DataSheet1.zip › Pictures of Phalloidine/1 μM Ang II + 1.2 mM Hirudin/1.277/63.jpg]

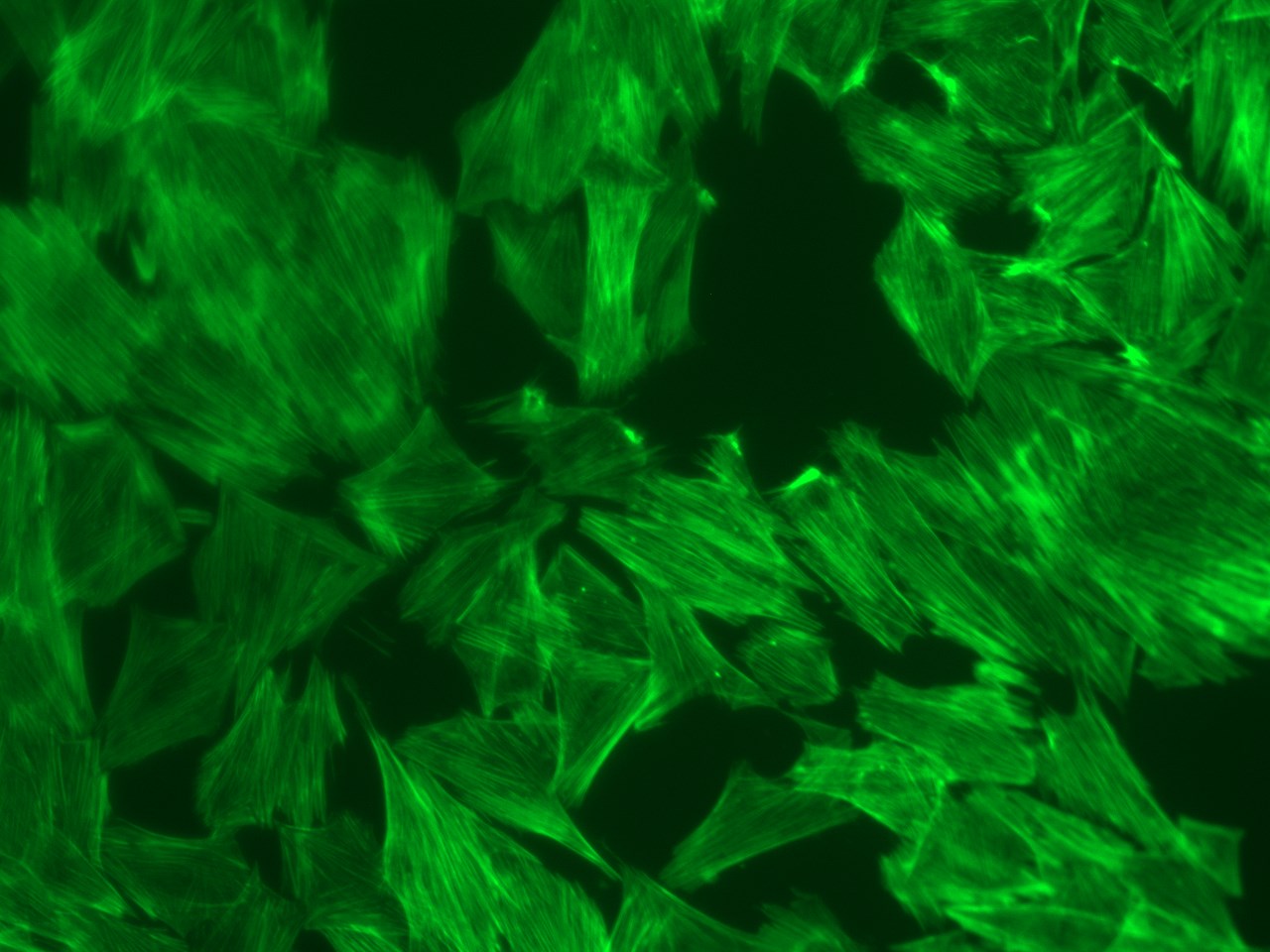

Supplement: Supplementary file 1 [file DataSheet1.zip › Pictures of Phalloidine/1 μM Ang II + 1.2 mM Hirudin/1.277/80.42.jpg]

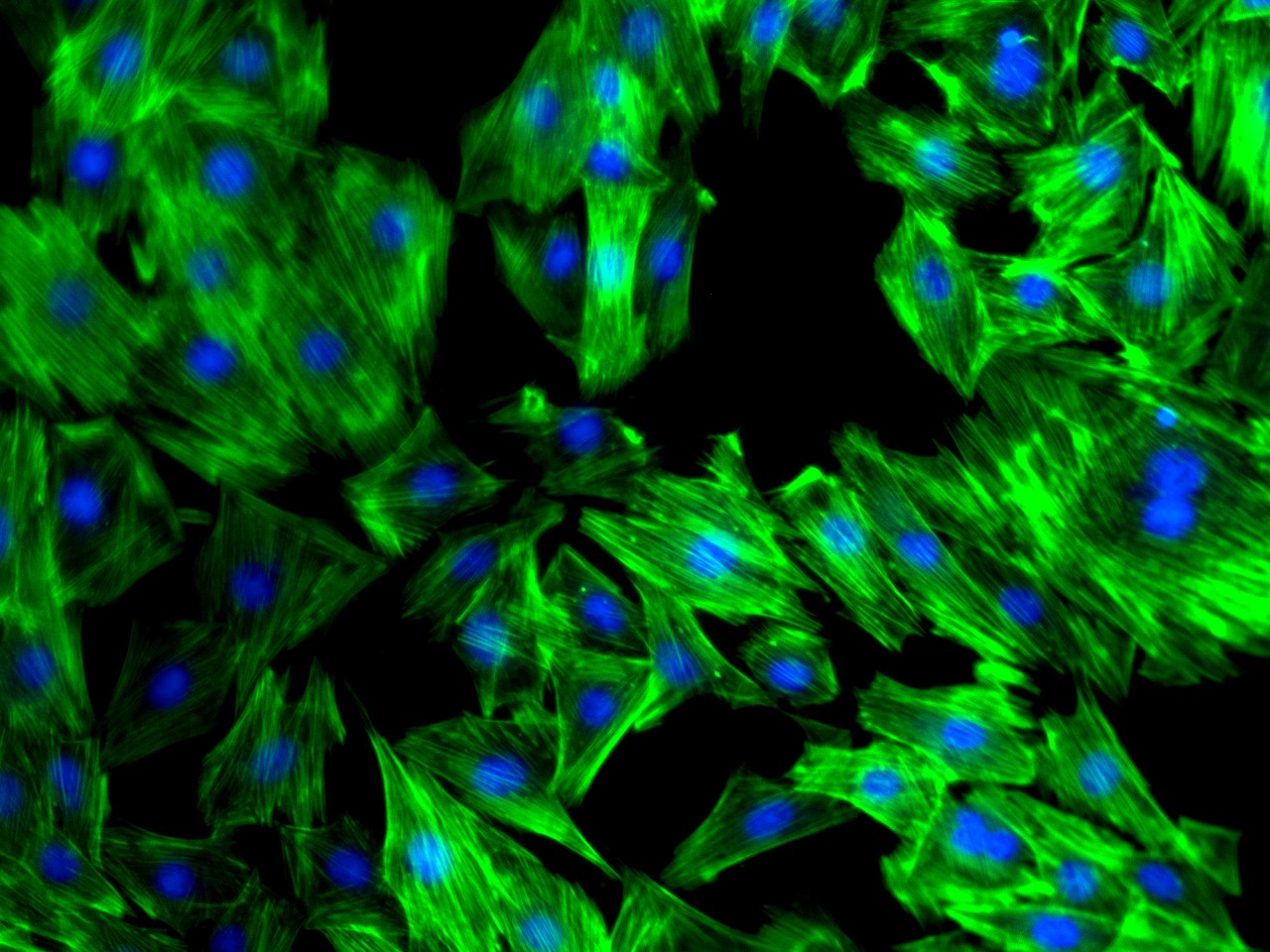

Supplement: Supplementary file 1 [file DataSheet1.zip › Pictures of Phalloidine/1 μM Ang II + 1.2 mM Hirudin/1.277/Merge.jpg]

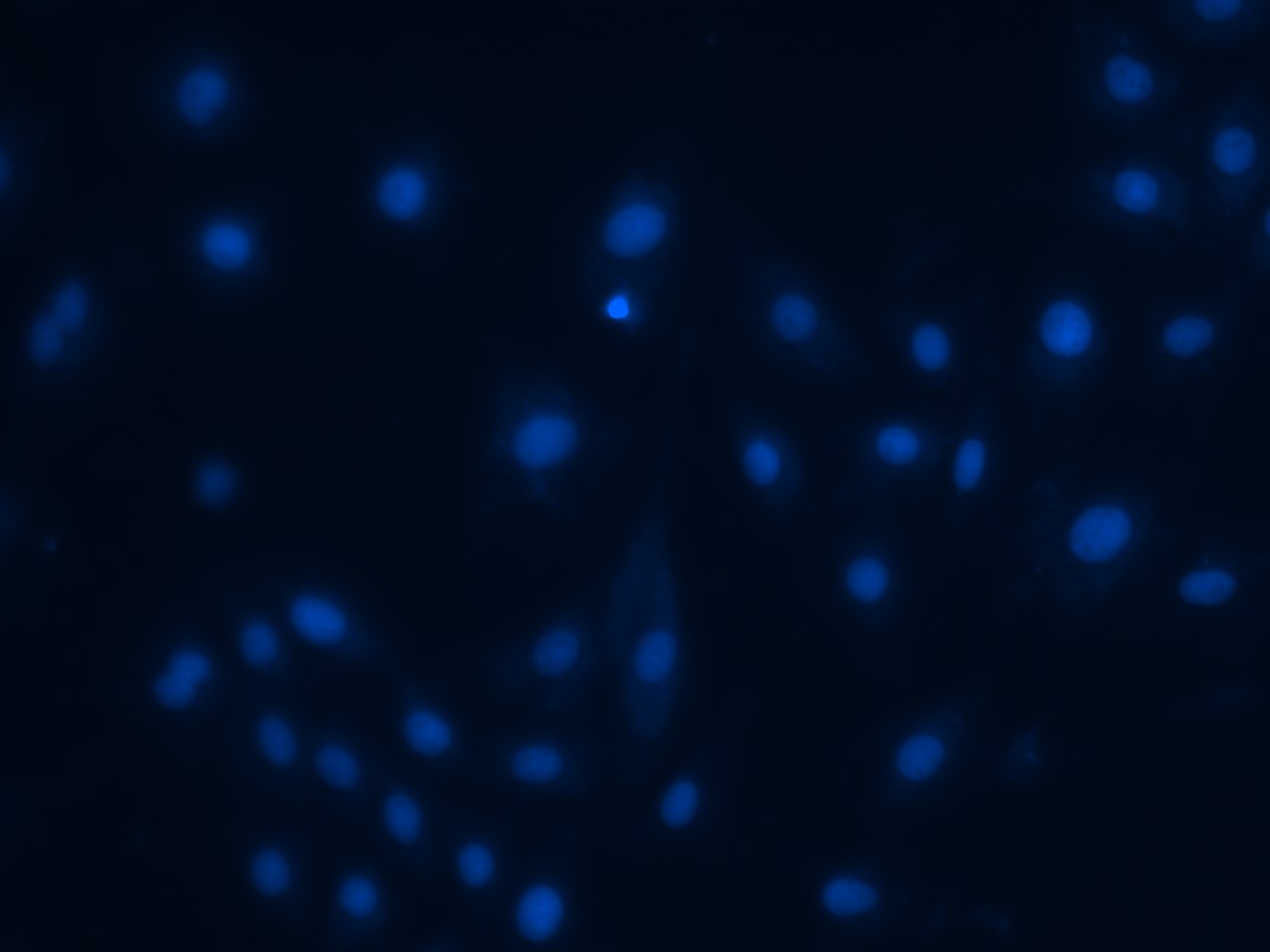

Supplement: Supplementary file 1 [file DataSheet1.zip › Pictures of Phalloidine/1 μM Ang II/1.716/38.jpg]

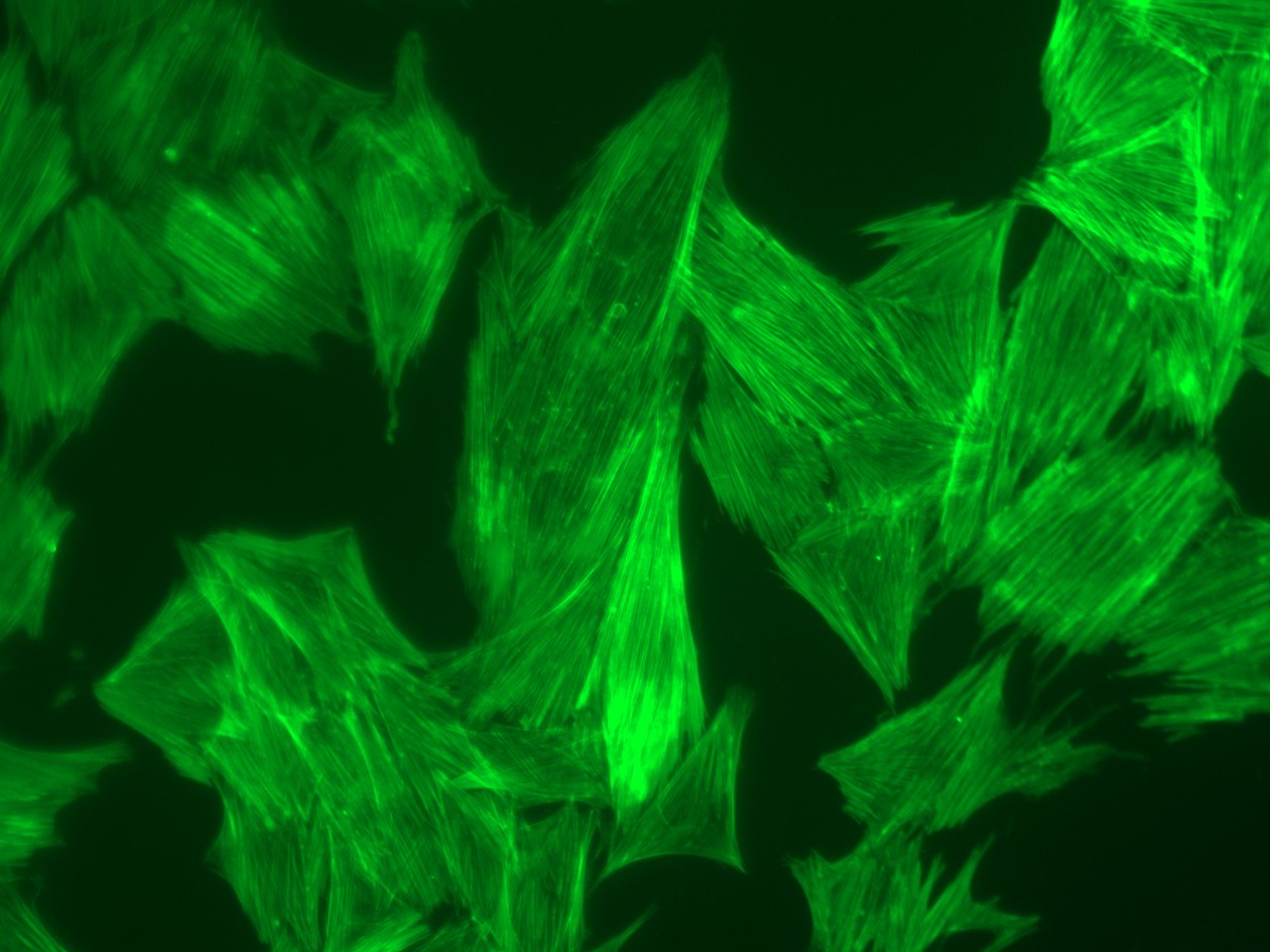

Supplement: Supplementary file 1 [file DataSheet1.zip › Pictures of Phalloidine/1 μM Ang II/1.716/65.213.jpg]

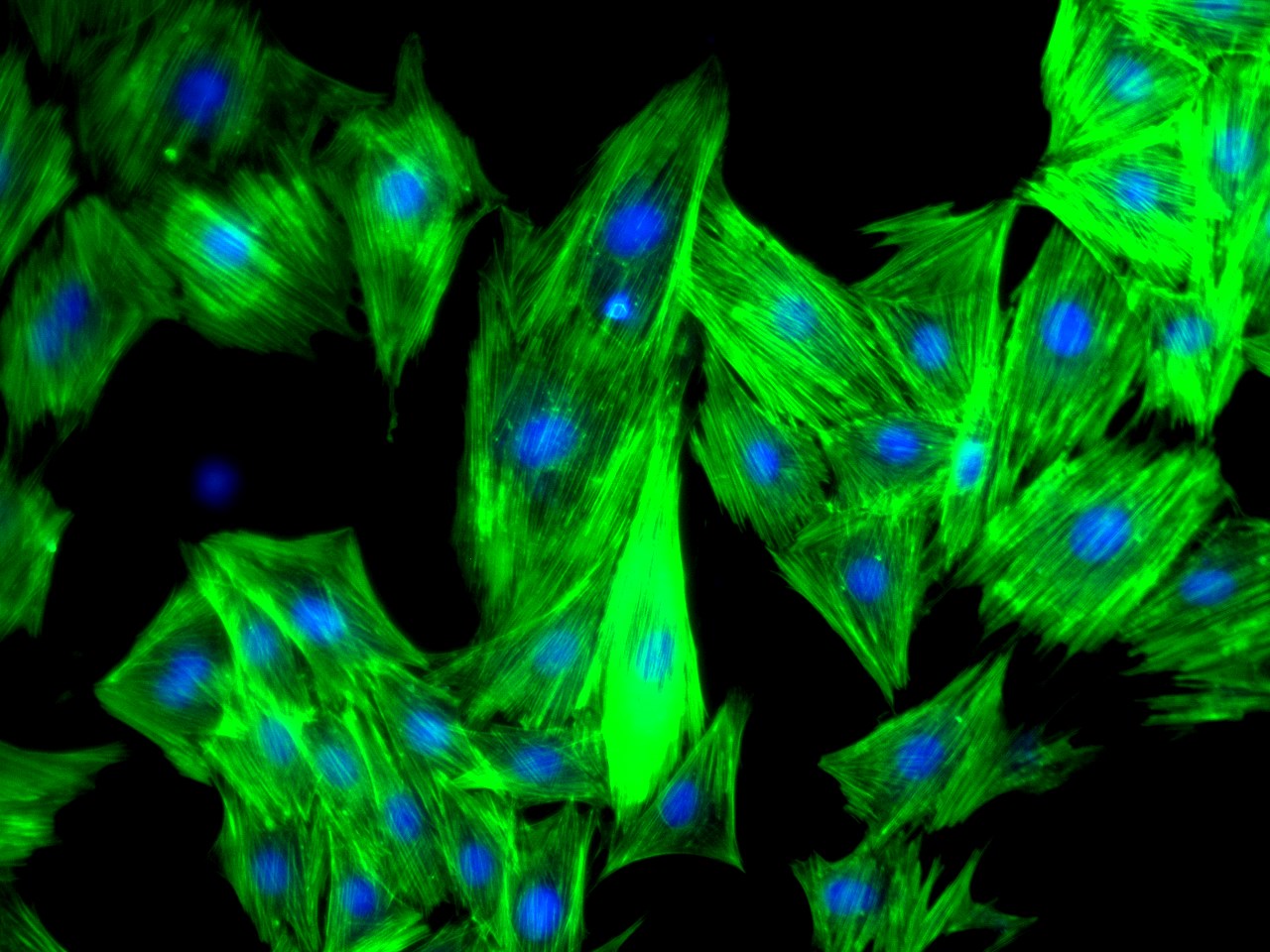

Supplement: Supplementary file 1 [file DataSheet1.zip › Pictures of Phalloidine/1 μM Ang II/1.716/Merge.jpg]

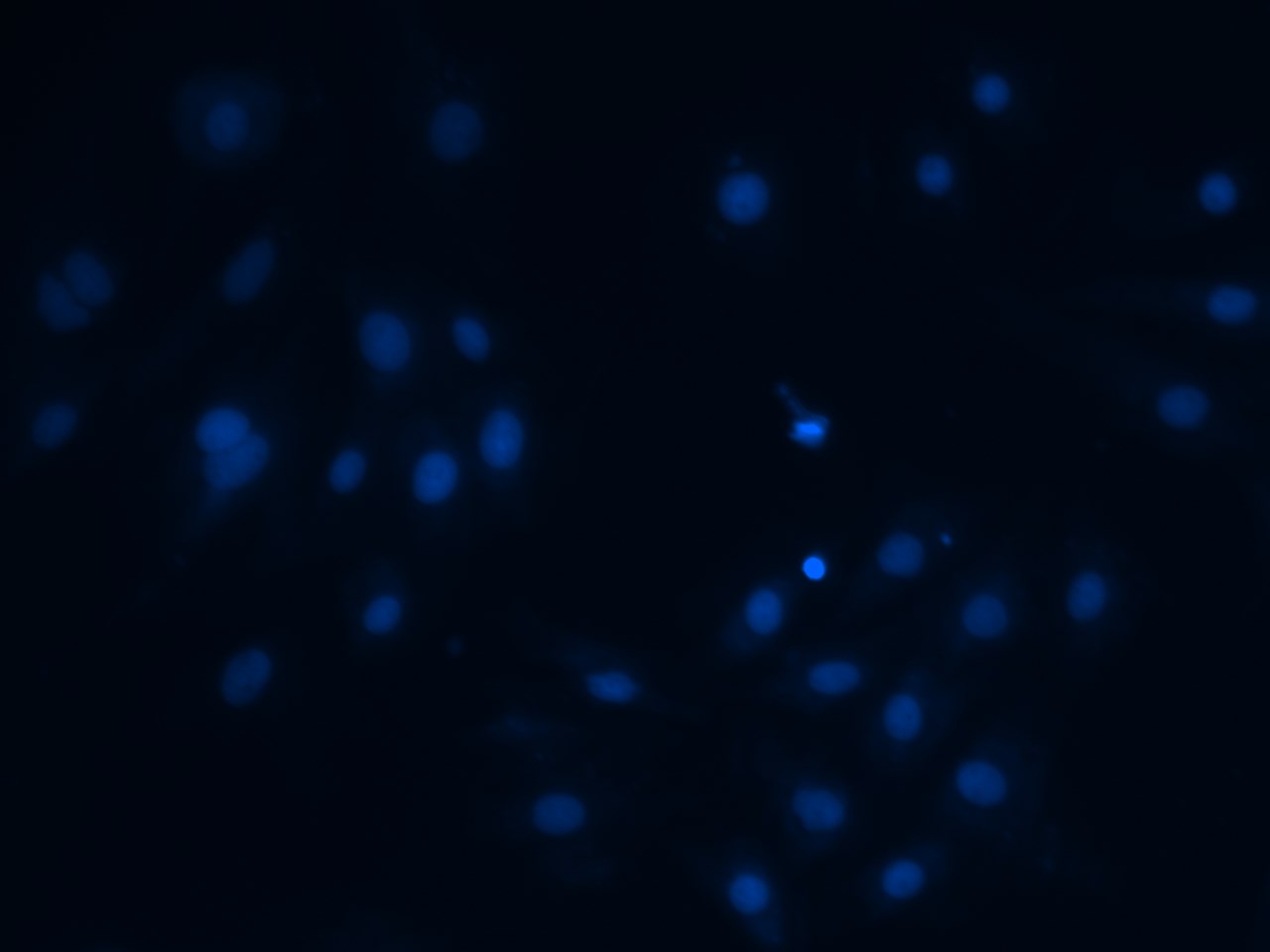

Supplement: Supplementary file 1 [file DataSheet1.zip › Pictures of Phalloidine/1 μM Ang II/1.763/33.jpg]

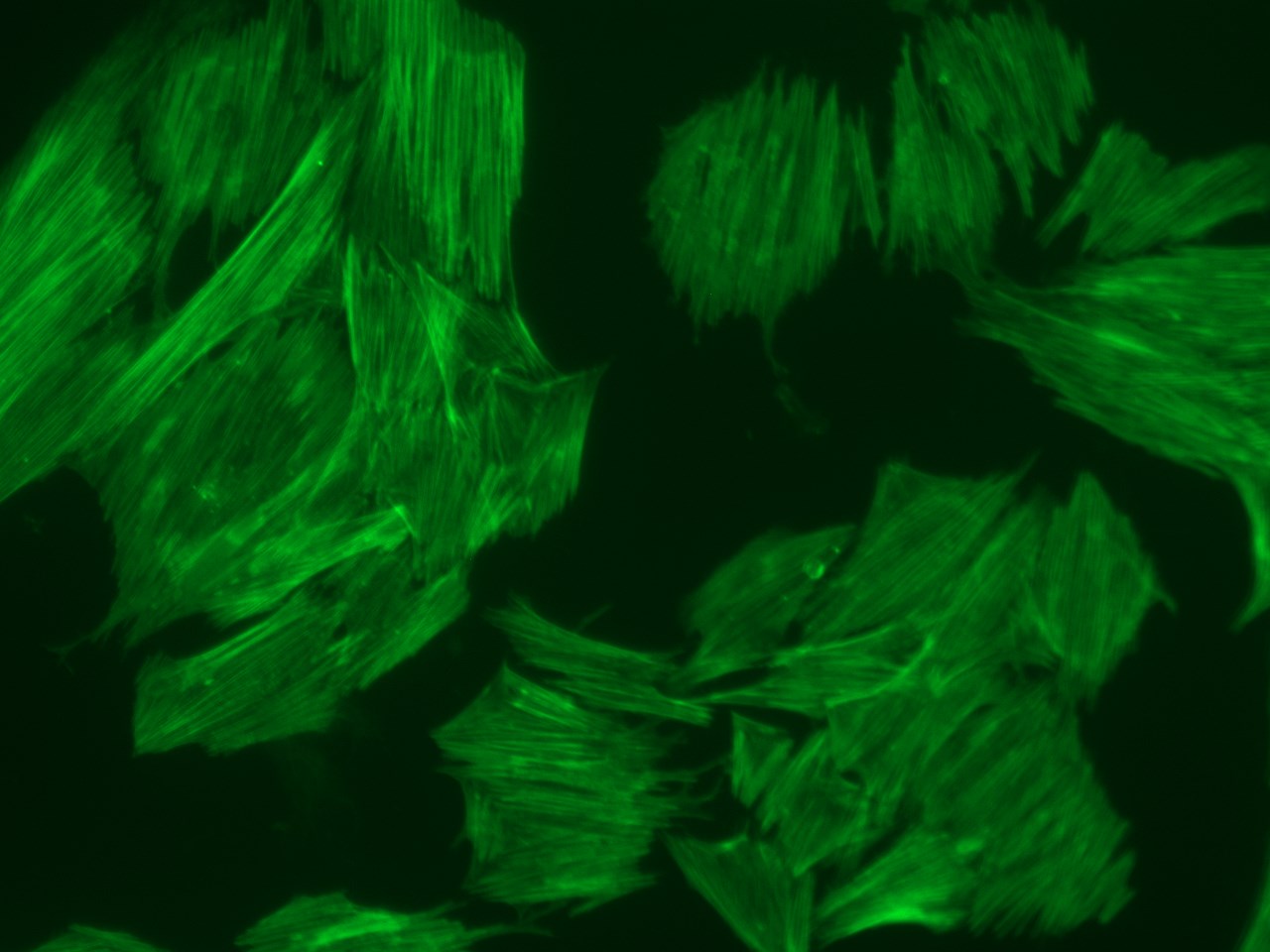

Supplement: Supplementary file 1 [file DataSheet1.zip › Pictures of Phalloidine/1 μM Ang II/1.763/58.191.jpg]

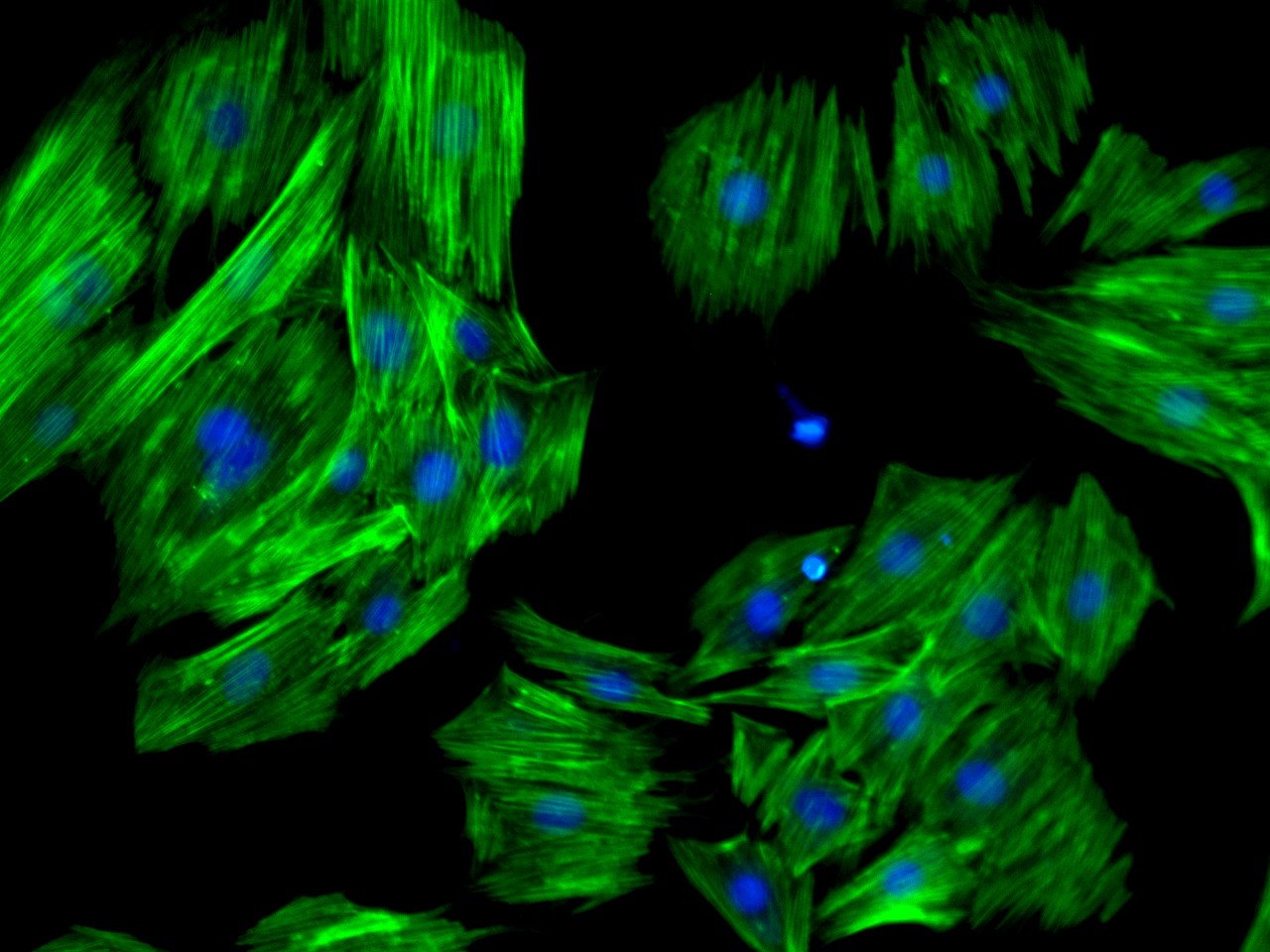

Supplement: Supplementary file 1 [file DataSheet1.zip › Pictures of Phalloidine/1 μM Ang II/1.763/Merge.jpg]

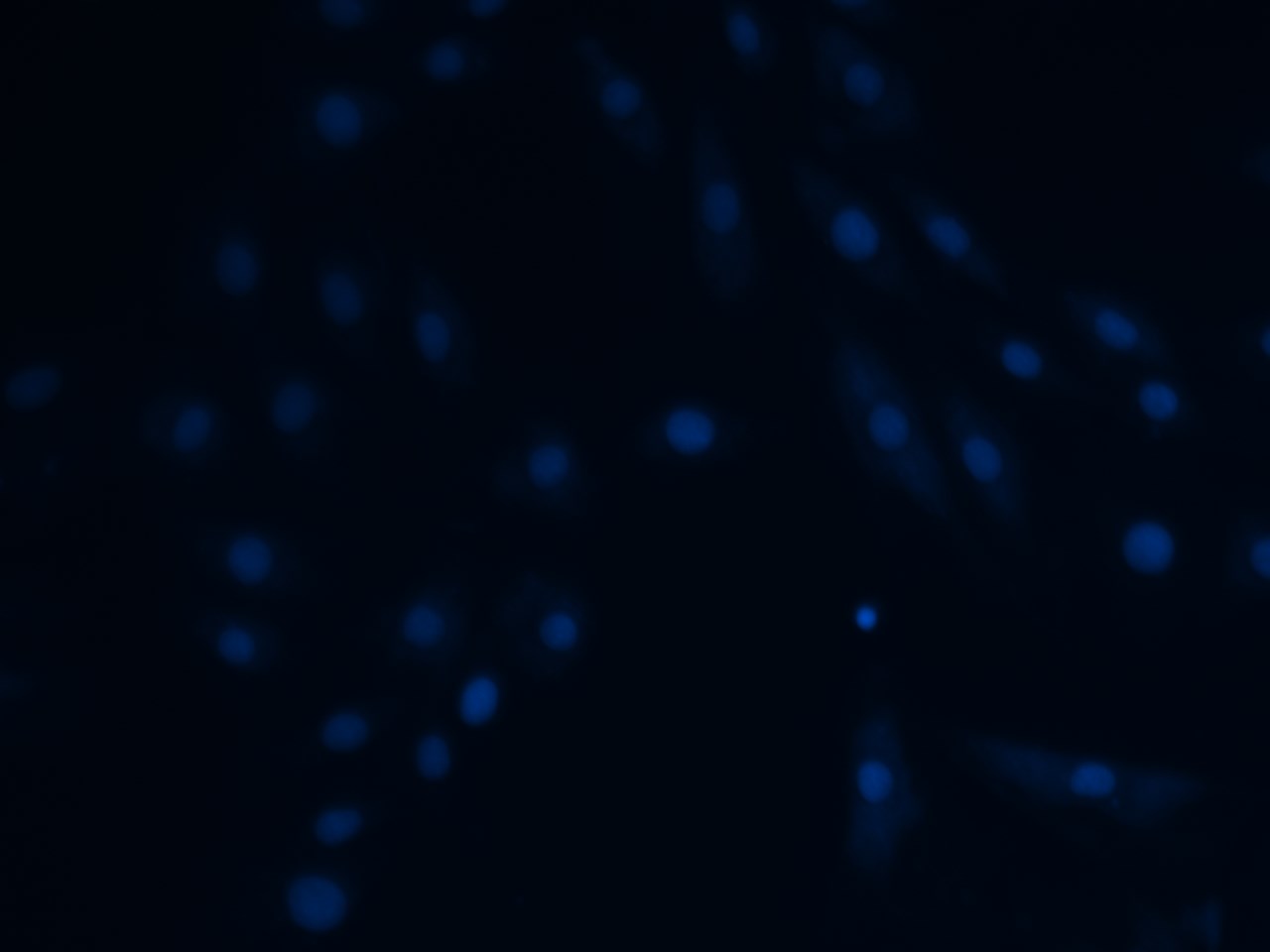

Supplement: Supplementary file 1 [file DataSheet1.zip › Pictures of Phalloidine/1 μM Ang II/1.826/33.jpg]

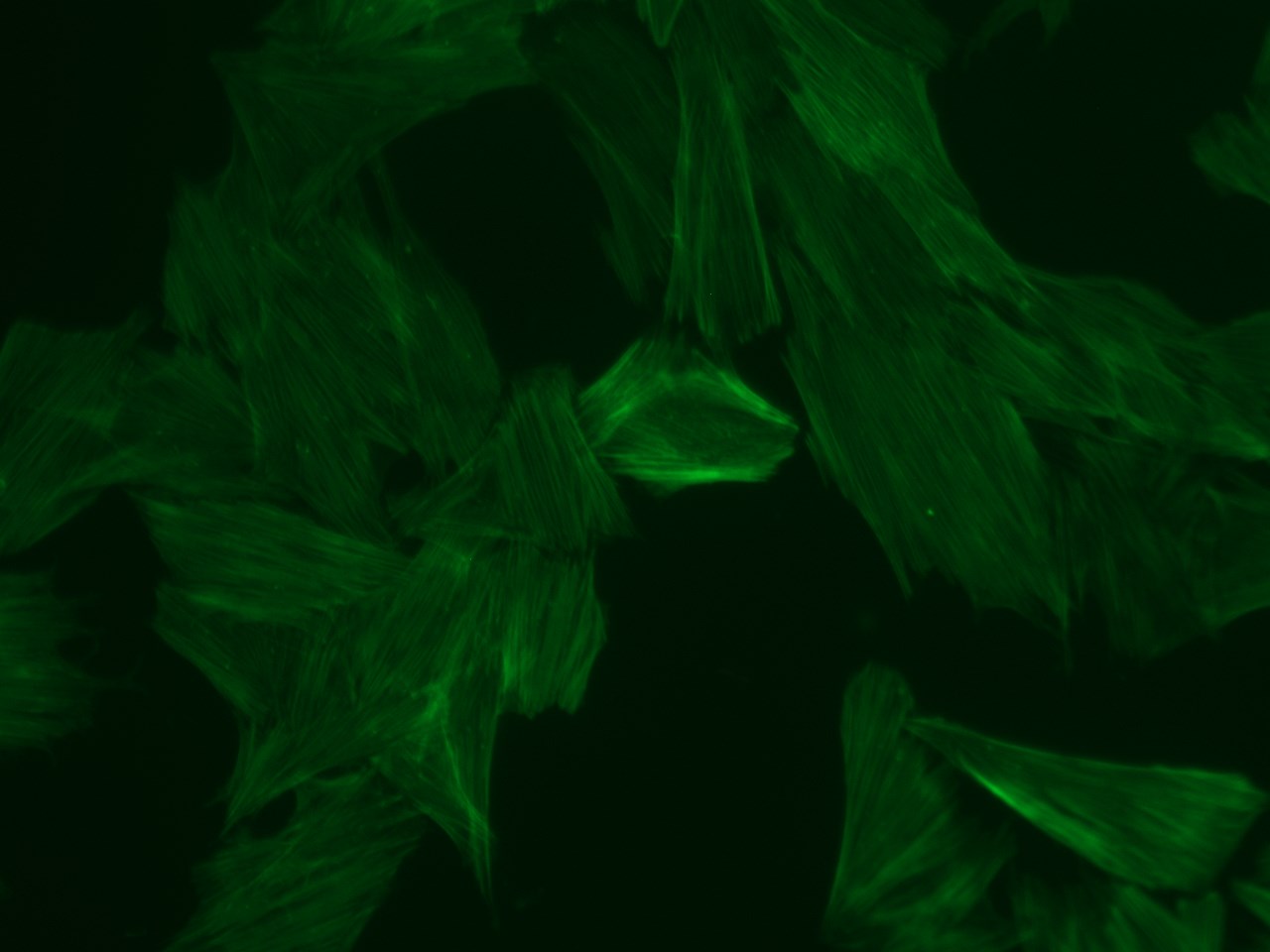

Supplement: Supplementary file 1 [file DataSheet1.zip › Pictures of Phalloidine/1 μM Ang II/1.826/60.270.jpg]

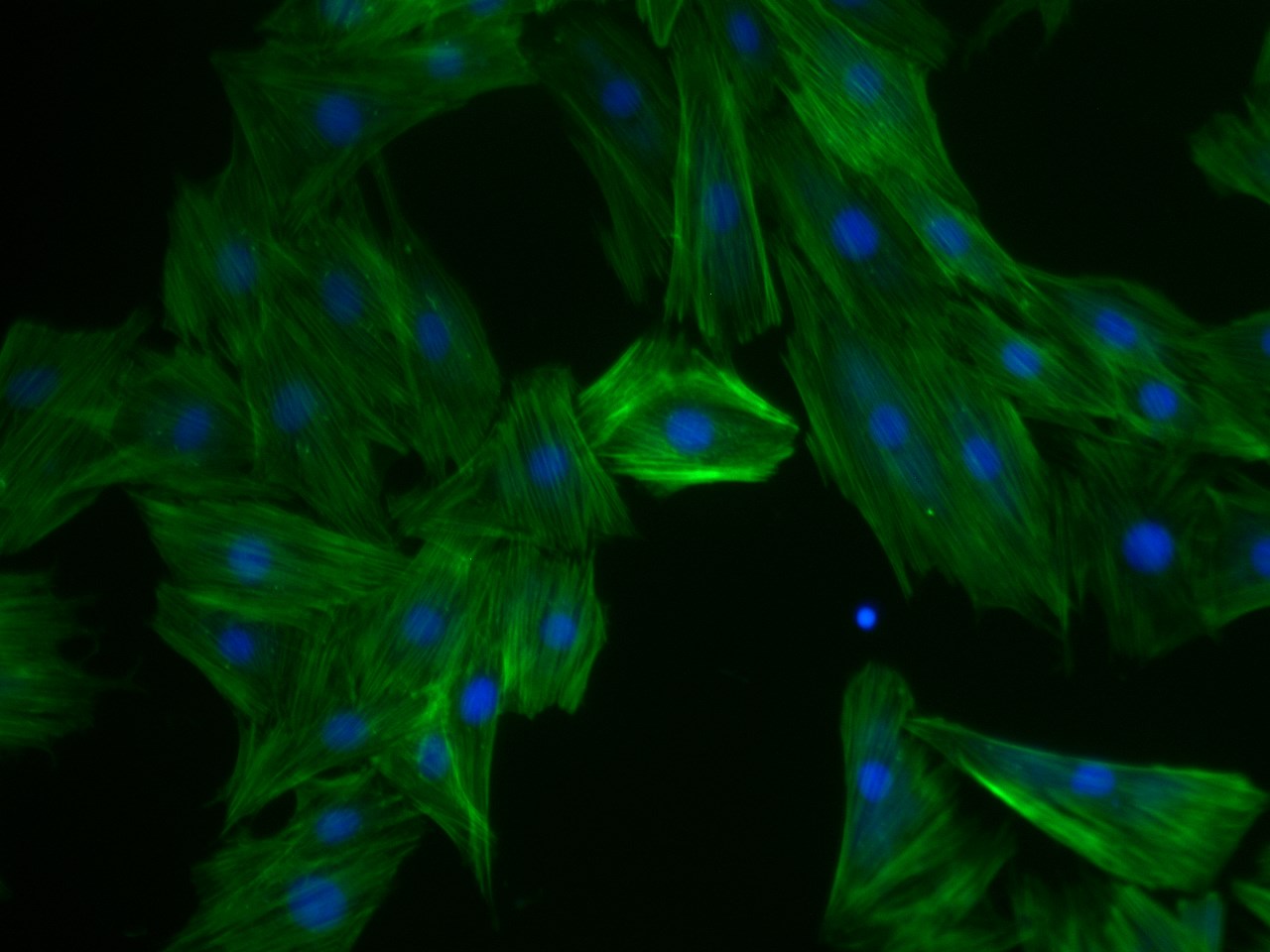

Supplement: Supplementary file 1 [file DataSheet1.zip › Pictures of Phalloidine/1 μM Ang II/1.826/Merge.jpg]

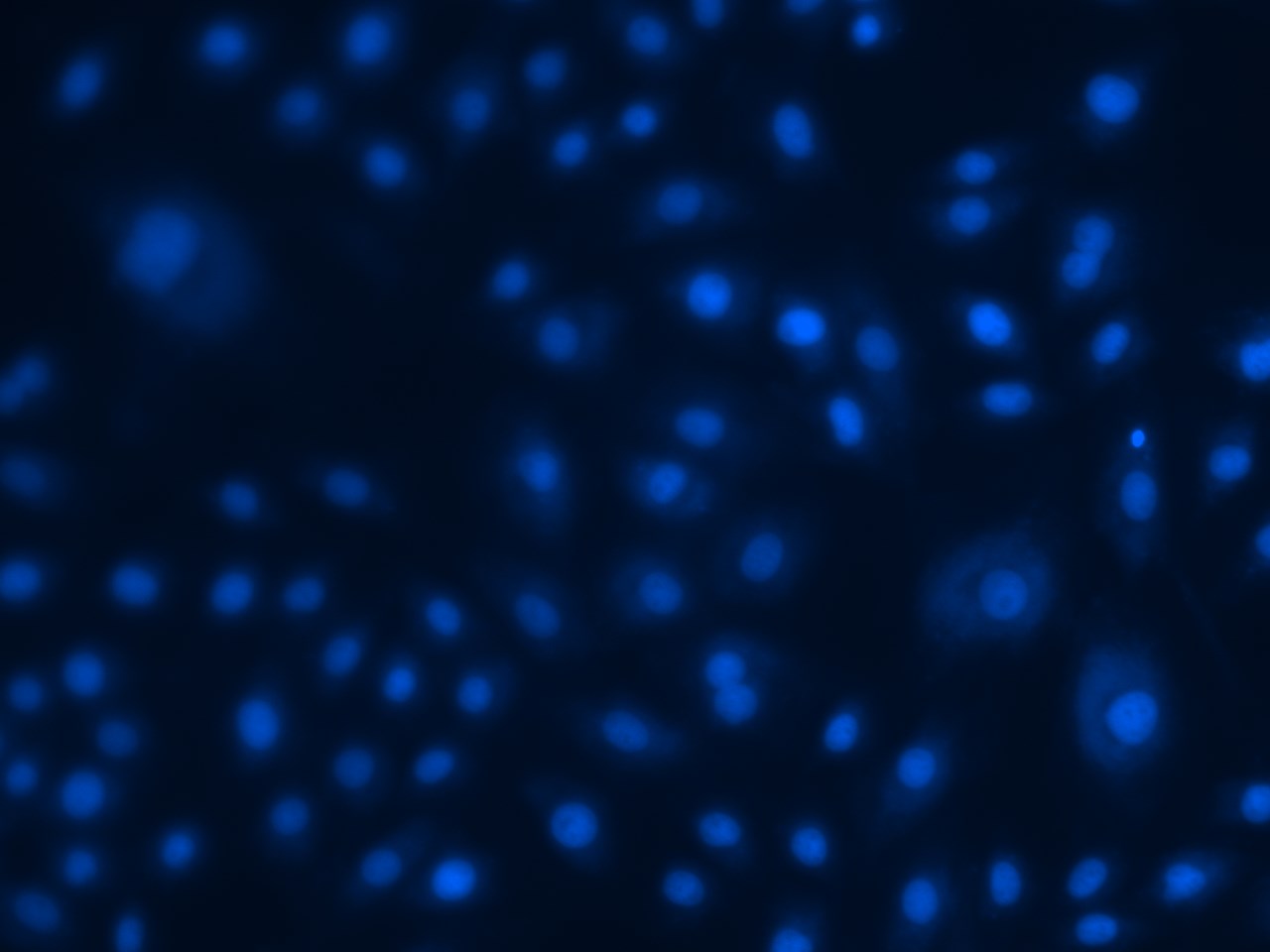

Supplement: Supplementary file 1 [file DataSheet1.zip › Pictures of Phalloidine/1μM Ang II + 10μM Losartan/1.059/83.jpg]

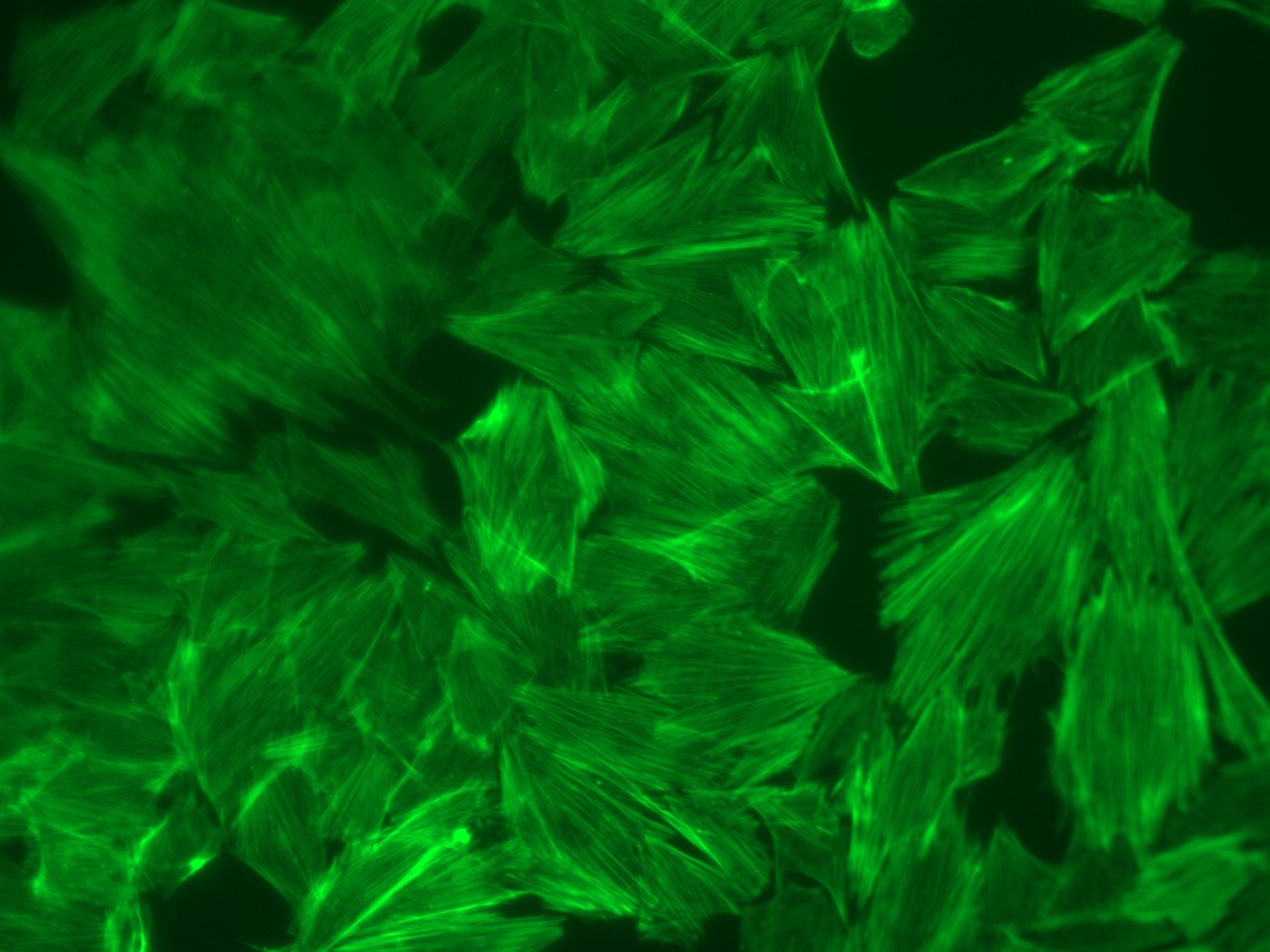

Supplement: Supplementary file 1 [file DataSheet1.zip › Pictures of Phalloidine/1μM Ang II + 10μM Losartan/1.059/87.894.jpg]

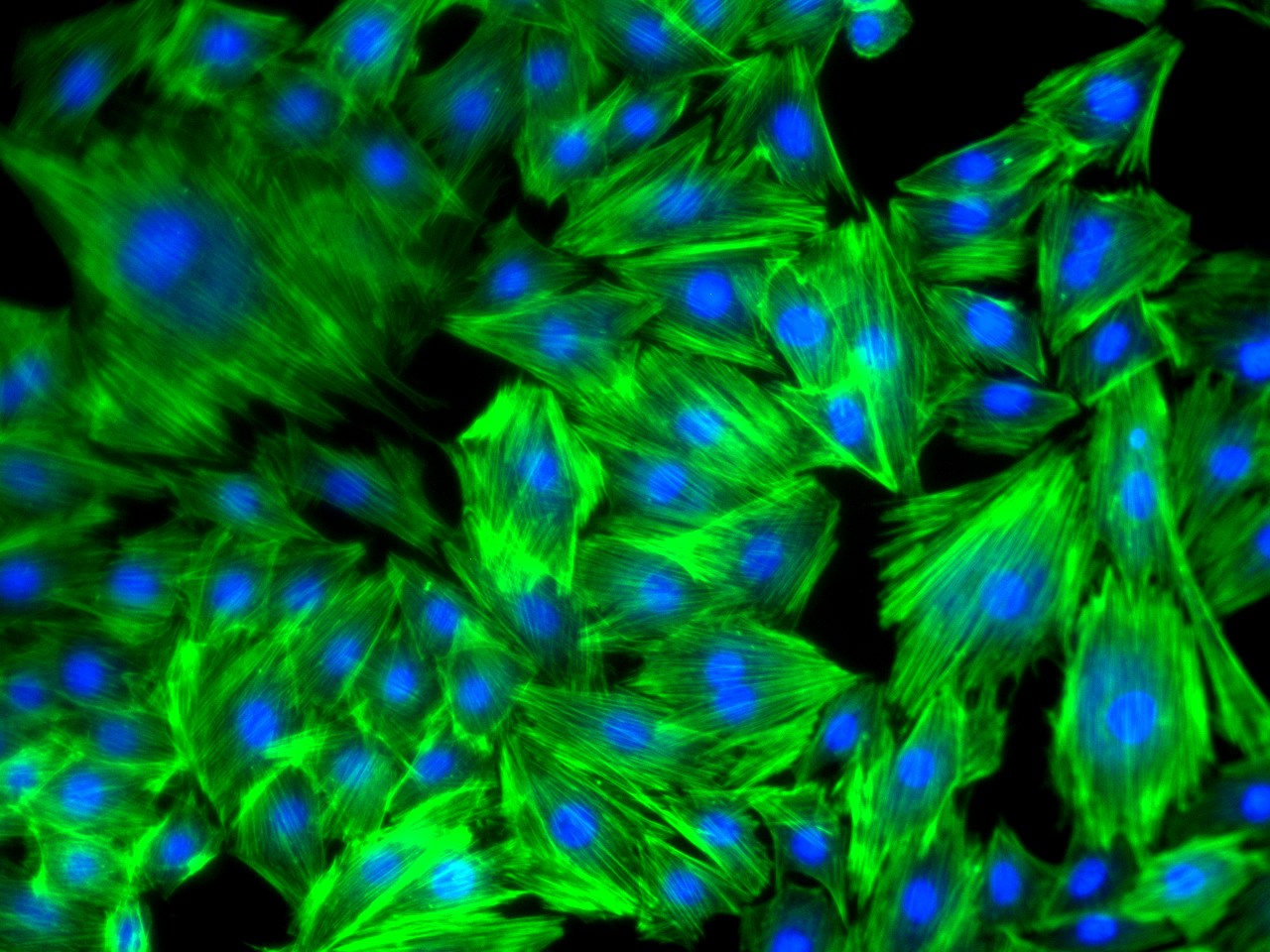

Supplement: Supplementary file 1 [file DataSheet1.zip › Pictures of Phalloidine/1μM Ang II + 10μM Losartan/1.059/Merge.jpg]

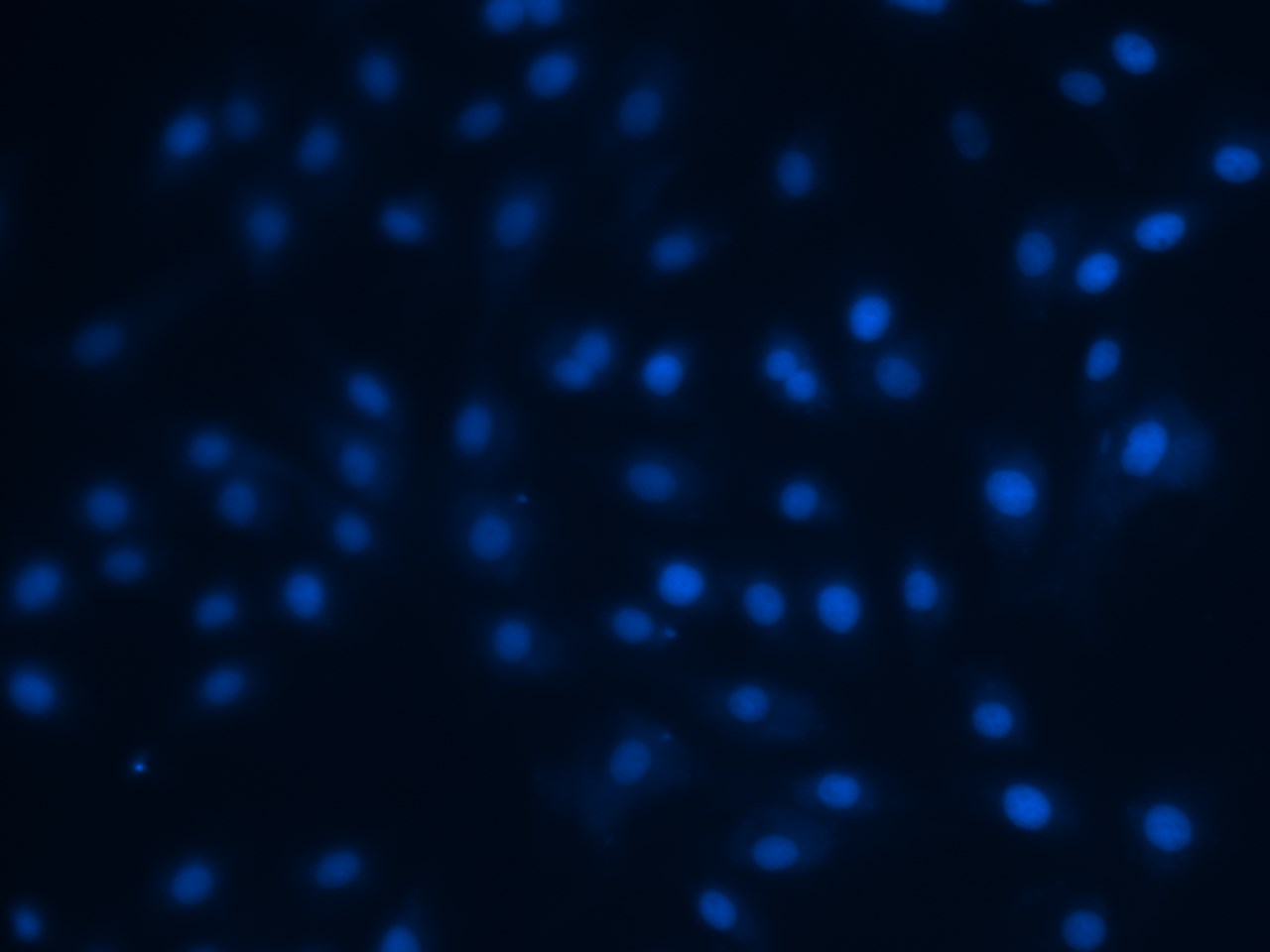

Supplement: Supplementary file 1 [file DataSheet1.zip › Pictures of Phalloidine/1μM Ang II + 10μM Losartan/1.112/67.jpg]

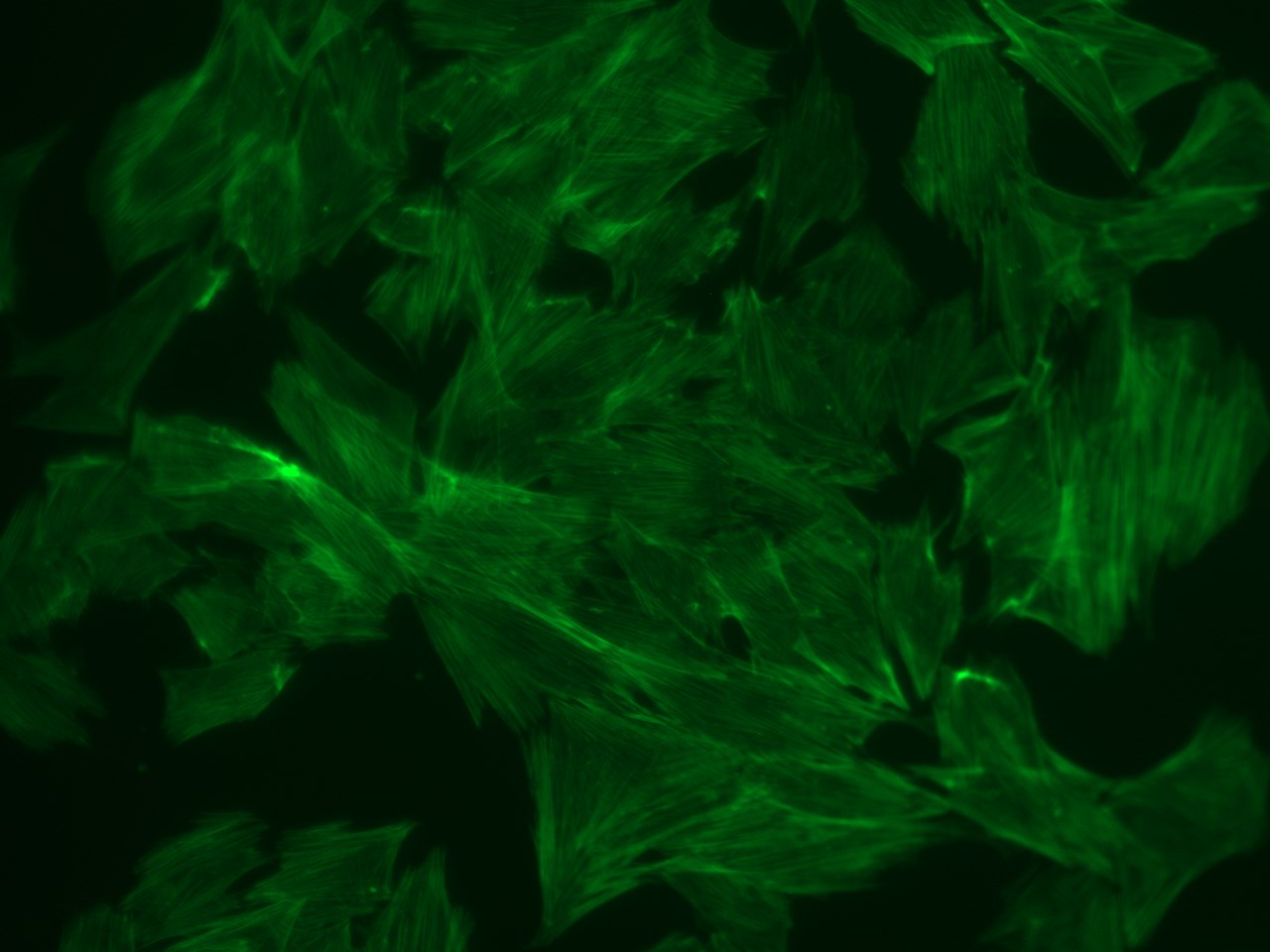

Supplement: Supplementary file 1 [file DataSheet1.zip › Pictures of Phalloidine/1μM Ang II + 10μM Losartan/1.112/74.521.jpg]

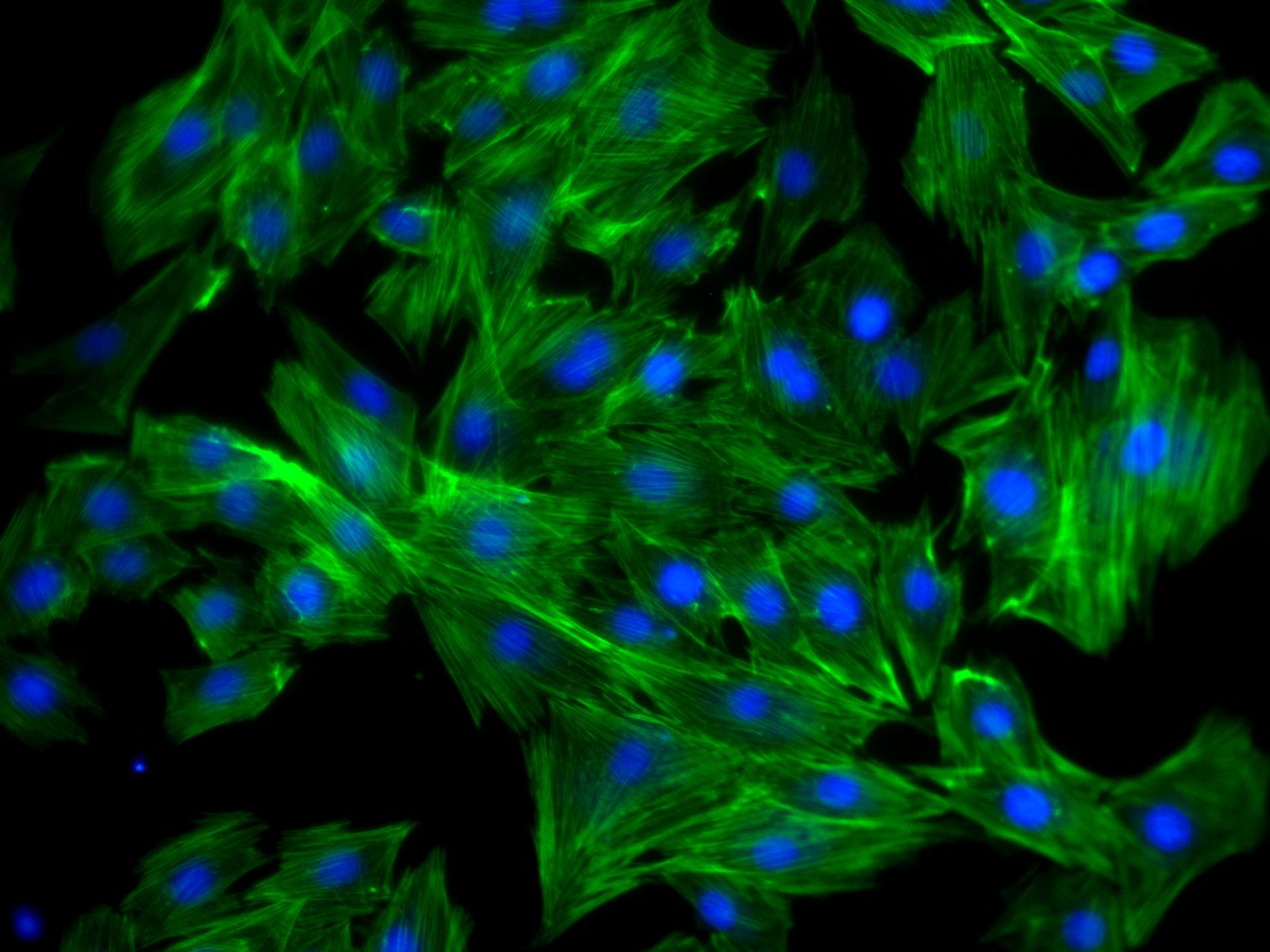

Supplement: Supplementary file 1 [file DataSheet1.zip › Pictures of Phalloidine/1μM Ang II + 10μM Losartan/1.112/Merge.jpg]

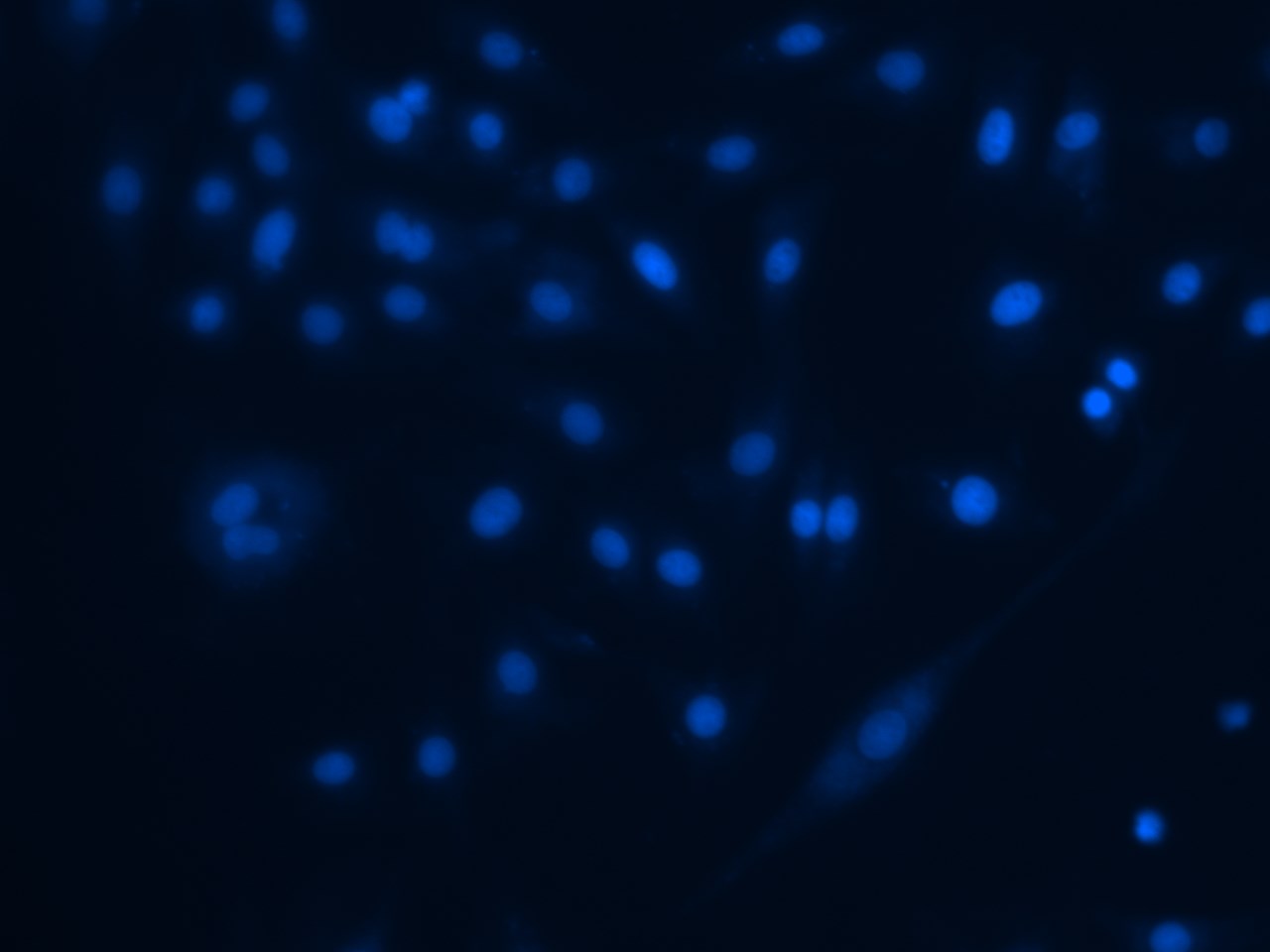

Supplement: Supplementary file 1 [file DataSheet1.zip › Pictures of Phalloidine/1μM Ang II + 10μM Losartan/1.171/45.jpg]

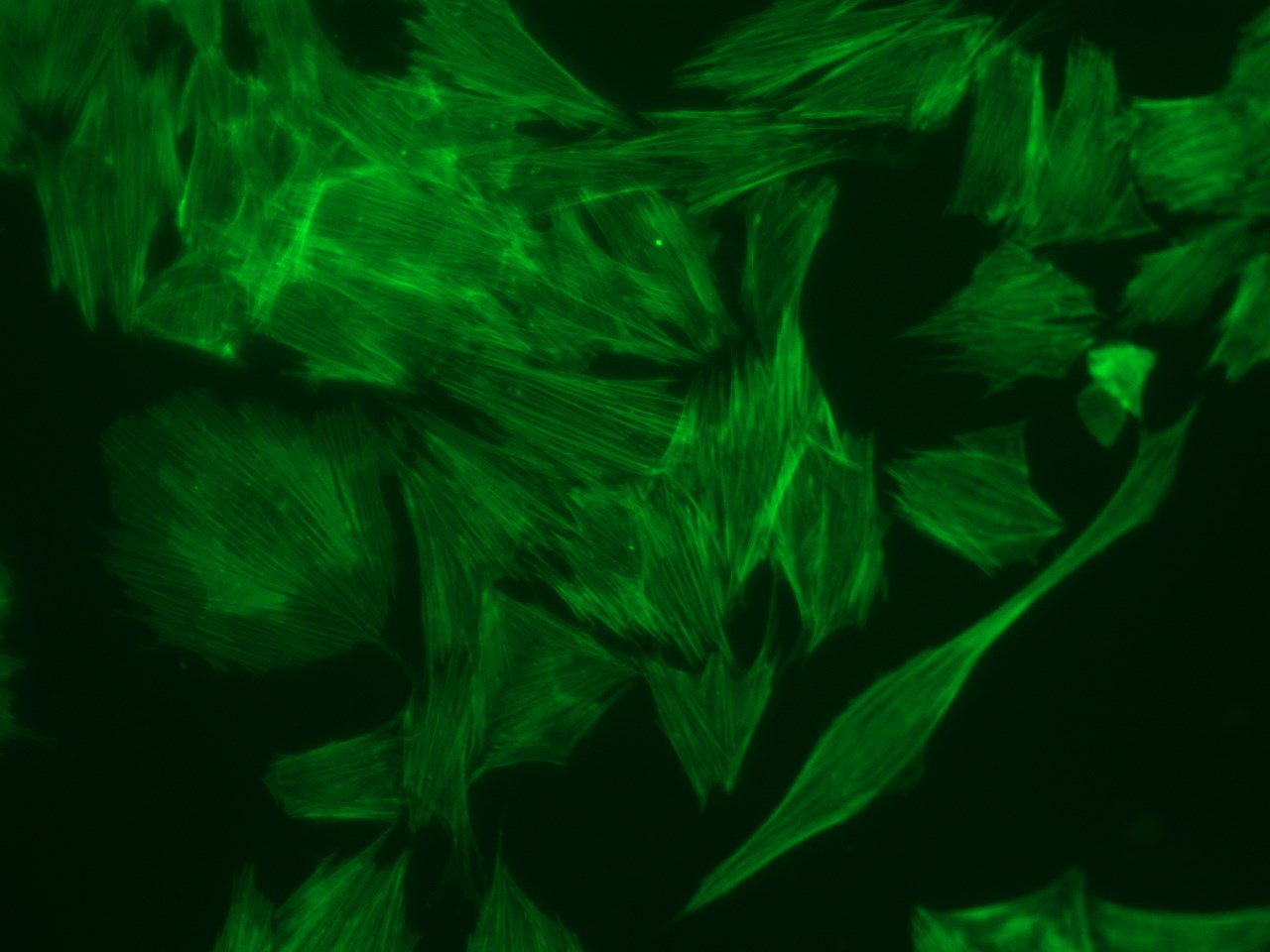

Supplement: Supplementary file 1 [file DataSheet1.zip › Pictures of Phalloidine/1μM Ang II + 10μM Losartan/1.171/52.697.jpg]

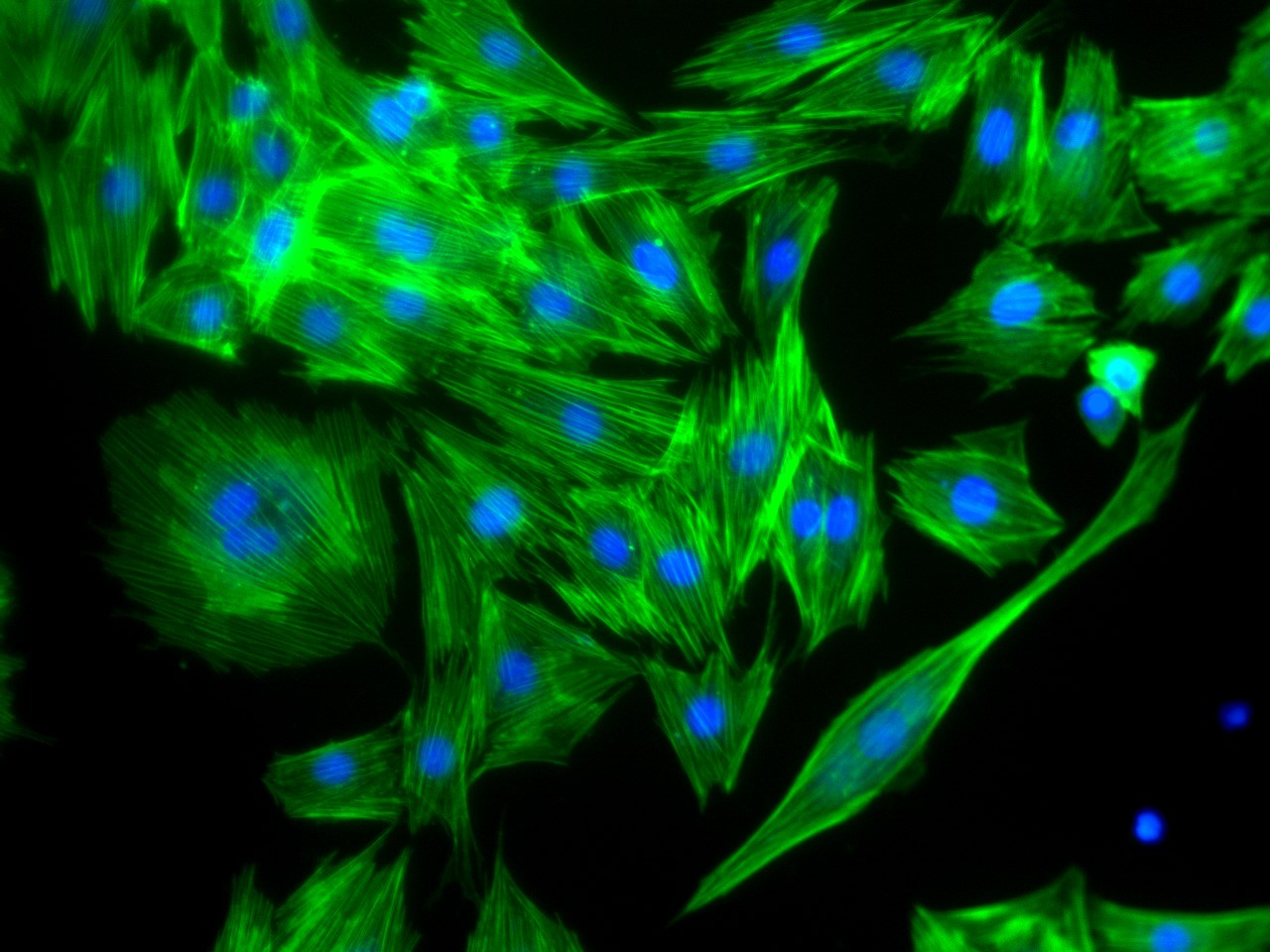

Supplement: Supplementary file 1 [file DataSheet1.zip › Pictures of Phalloidine/1μM Ang II + 10μM Losartan/1.171/Merge.jpg]

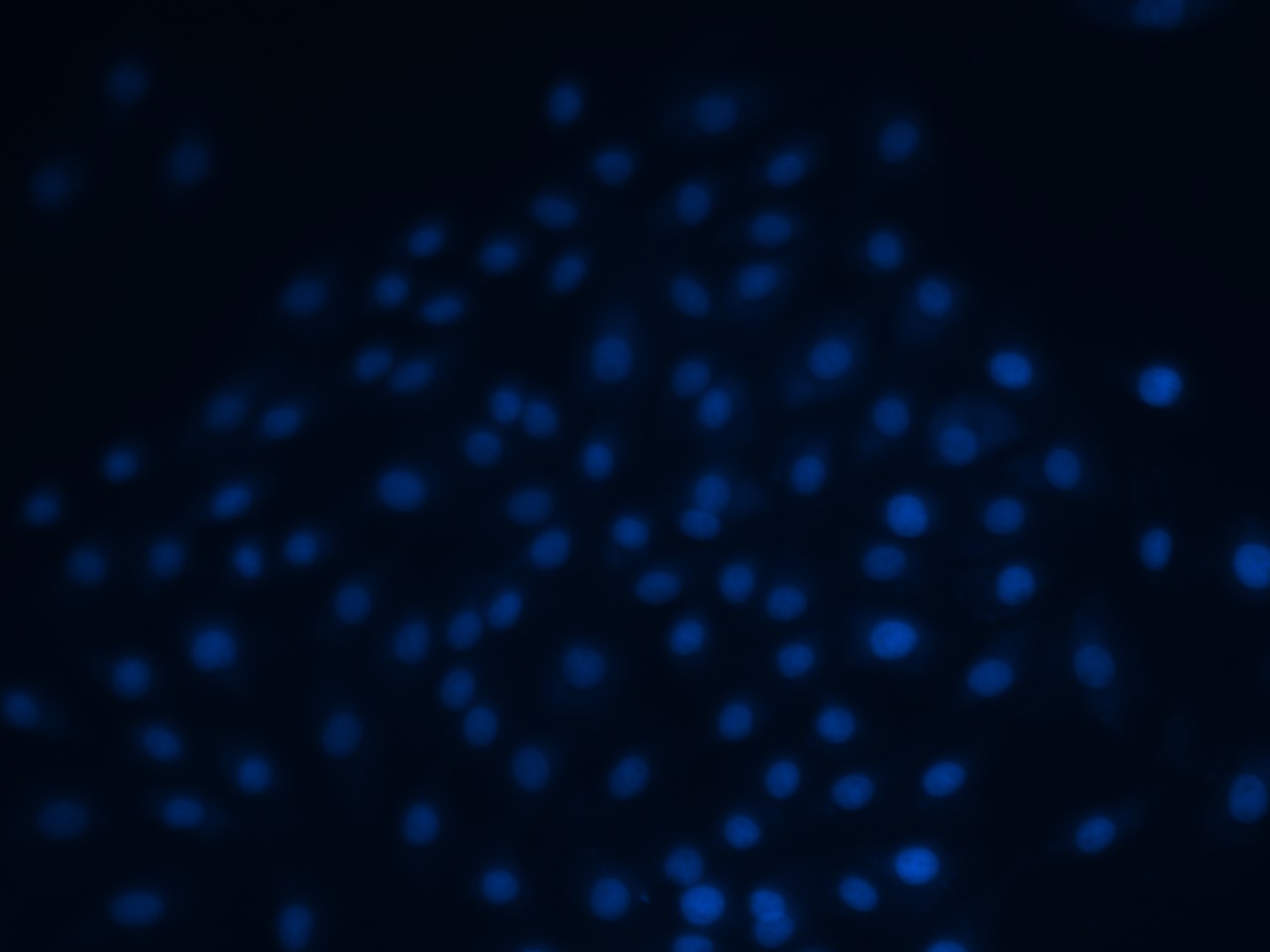

Supplement: Supplementary file 1 [file DataSheet1.zip › Pictures of Phalloidine/Control/0.792/101.jpg]

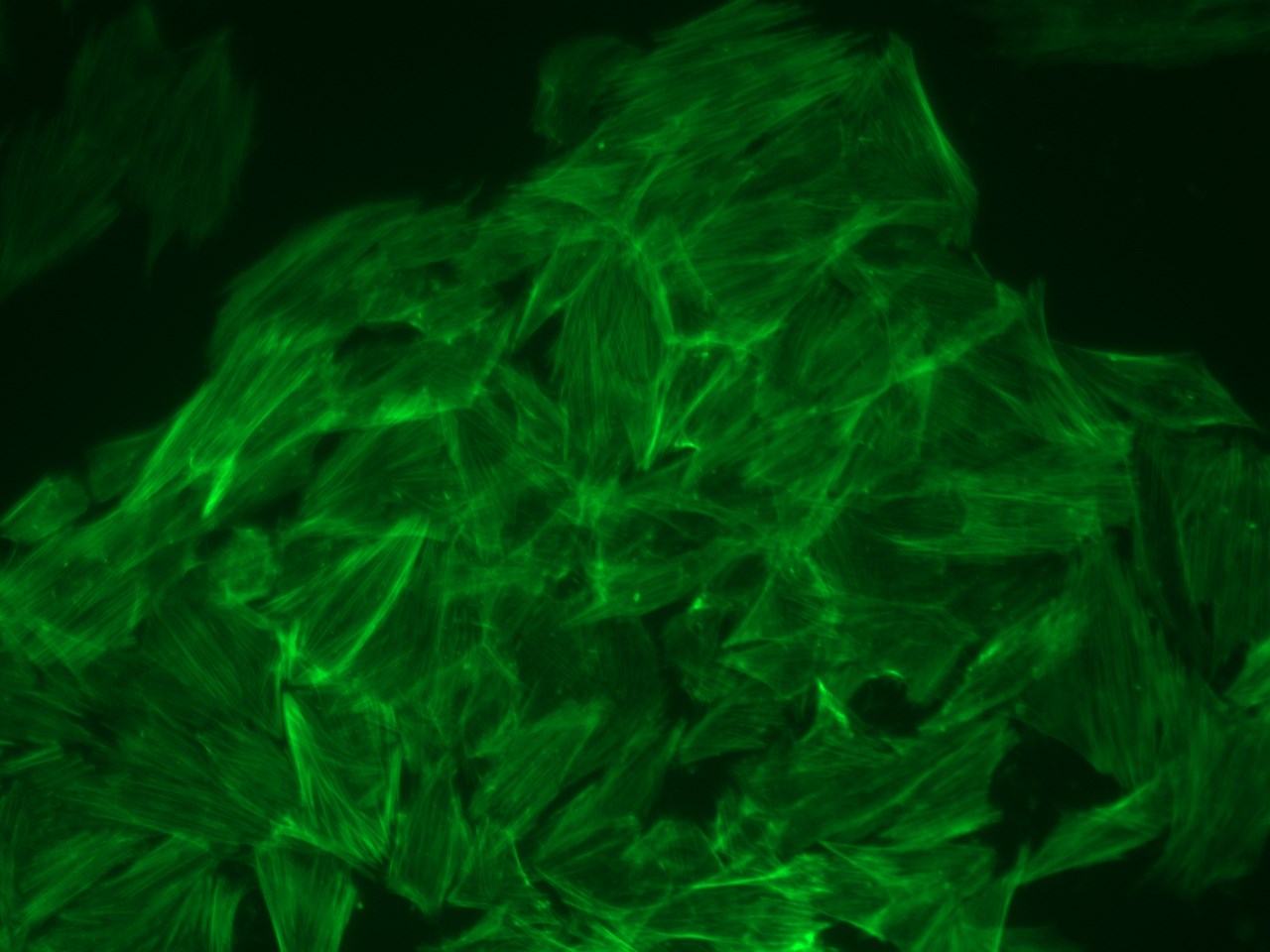

Supplement: Supplementary file 1 [file DataSheet1.zip › Pictures of Phalloidine/Control/0.792/79.976.jpg]

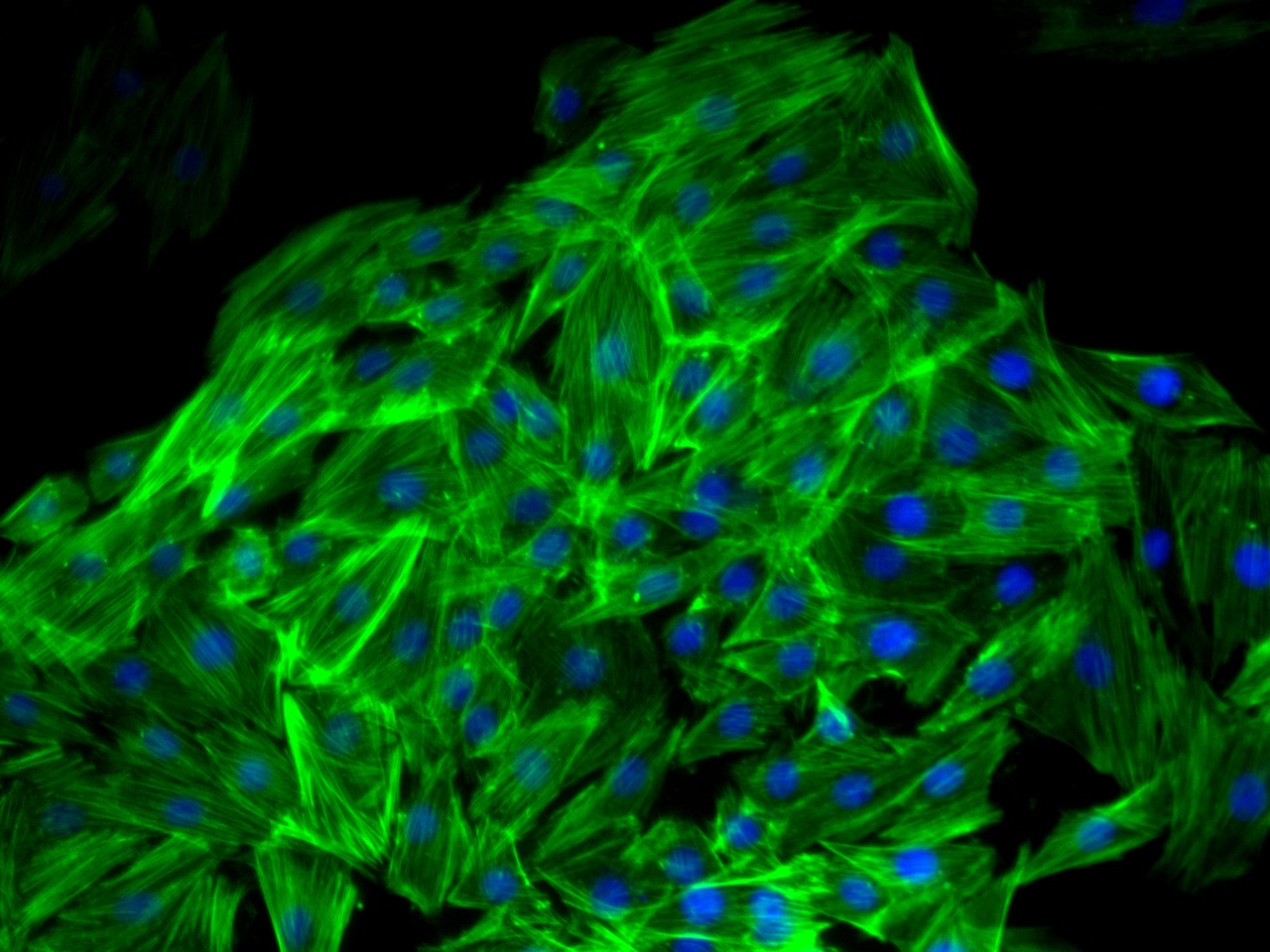

Supplement: Supplementary file 1 [file DataSheet1.zip › Pictures of Phalloidine/Control/0.792/Merge.jpg]

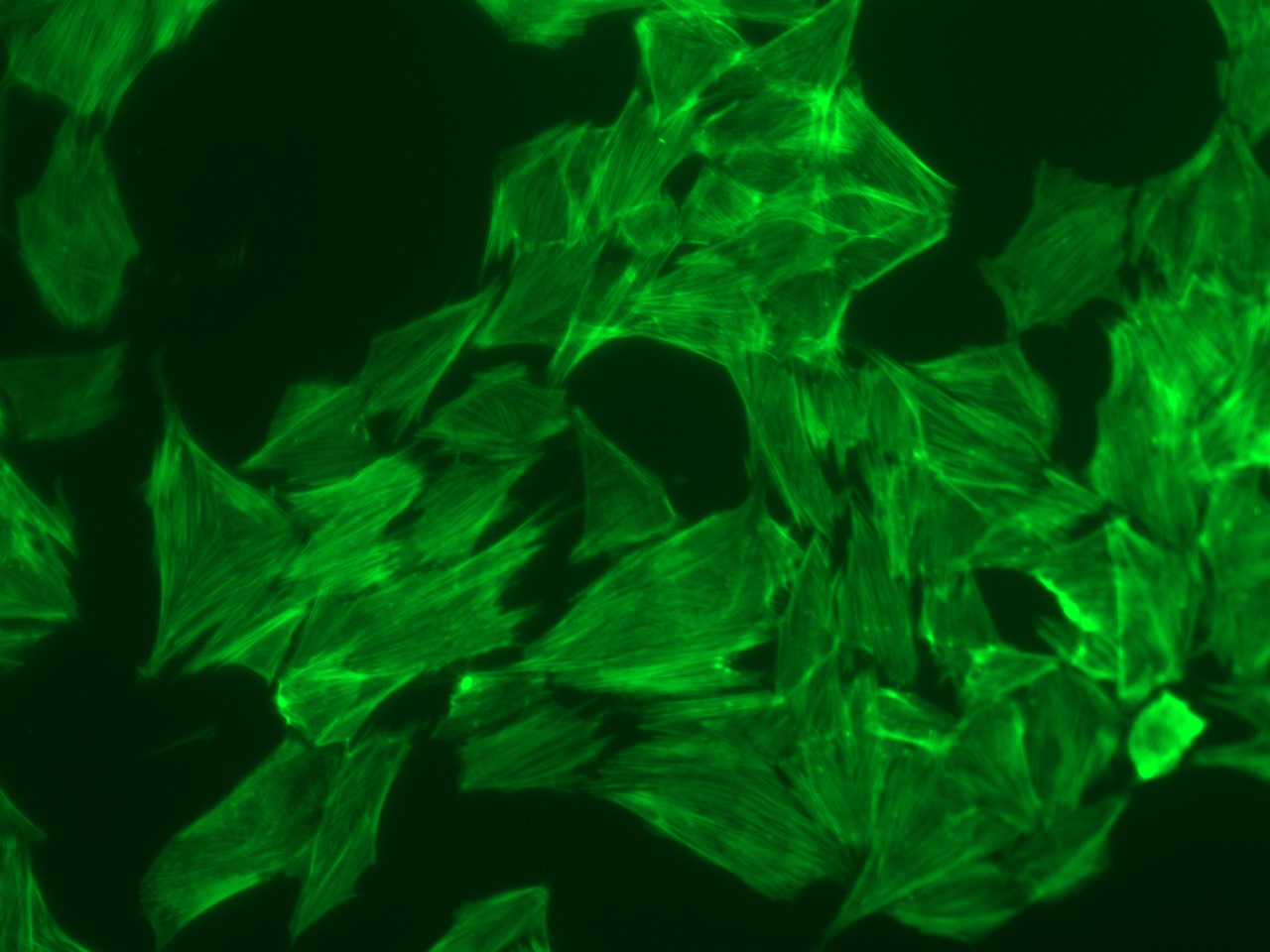

Supplement: Supplementary file 1 [file DataSheet1.zip › Pictures of Phalloidine/Control/0.887/58.545.jpg]

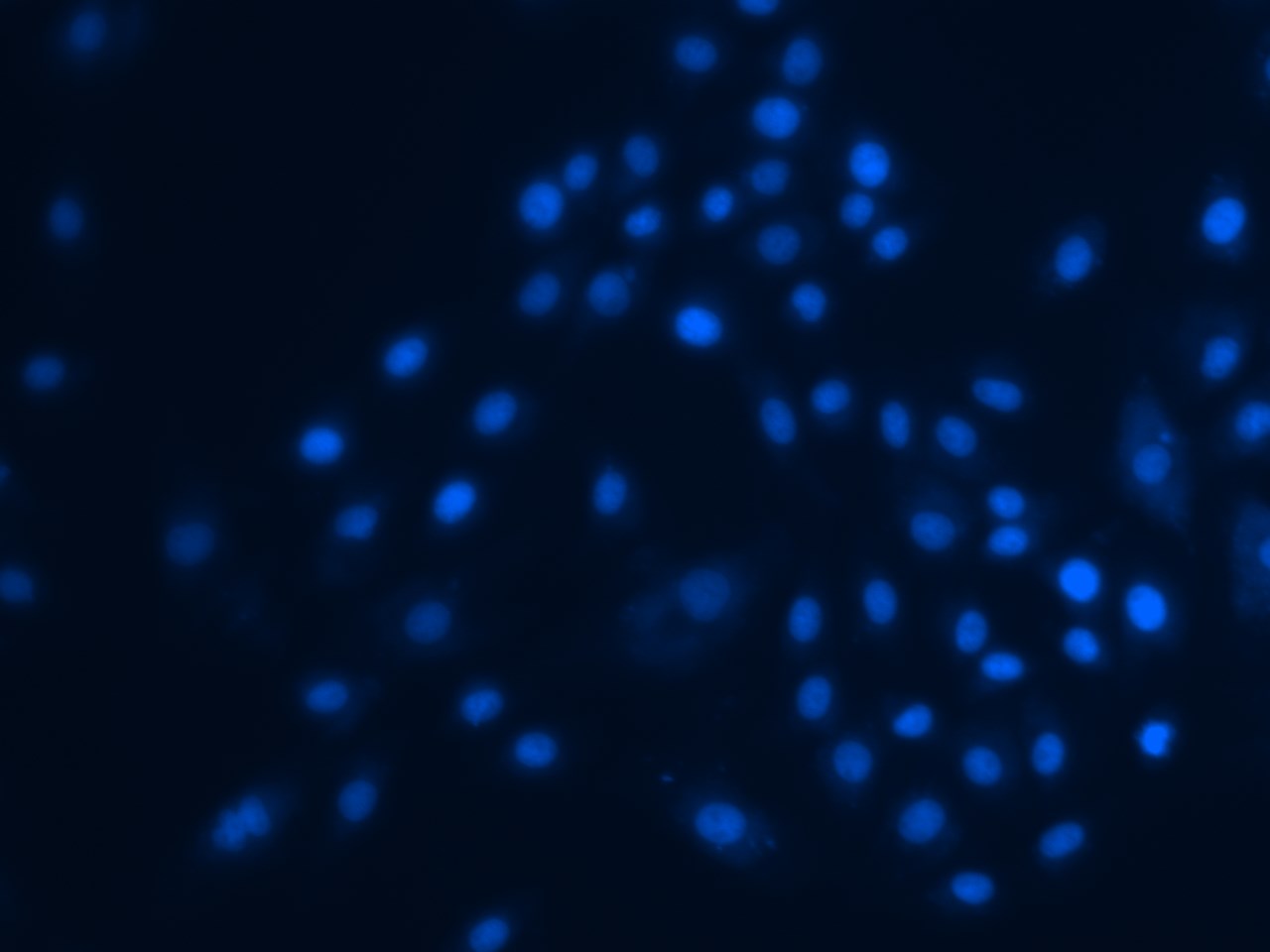

Supplement: Supplementary file 1 [file DataSheet1.zip › Pictures of Phalloidine/Control/0.887/66.jpg]

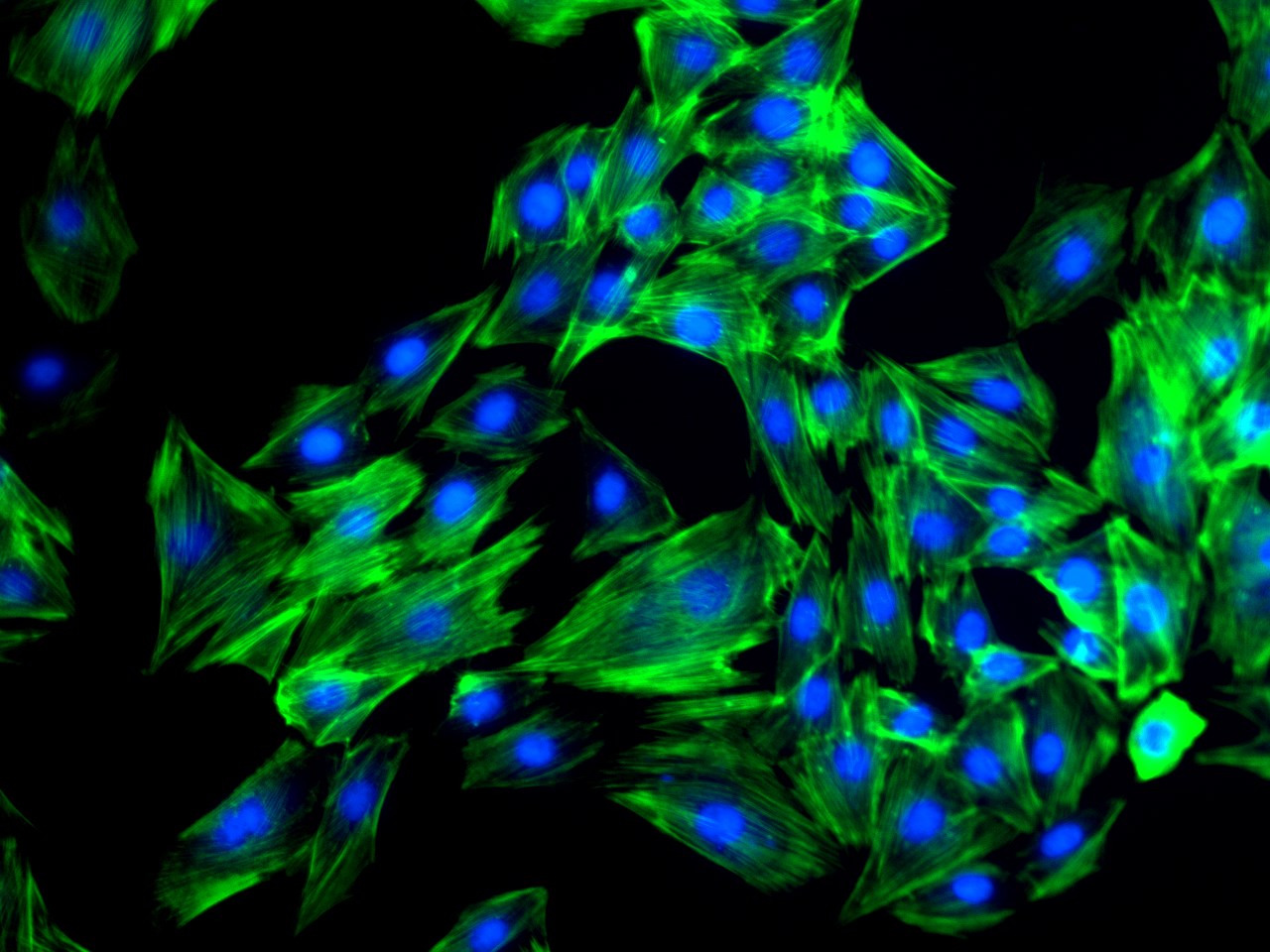

Supplement: Supplementary file 1 [file DataSheet1.zip › Pictures of Phalloidine/Control/0.887/Merge.jpg]

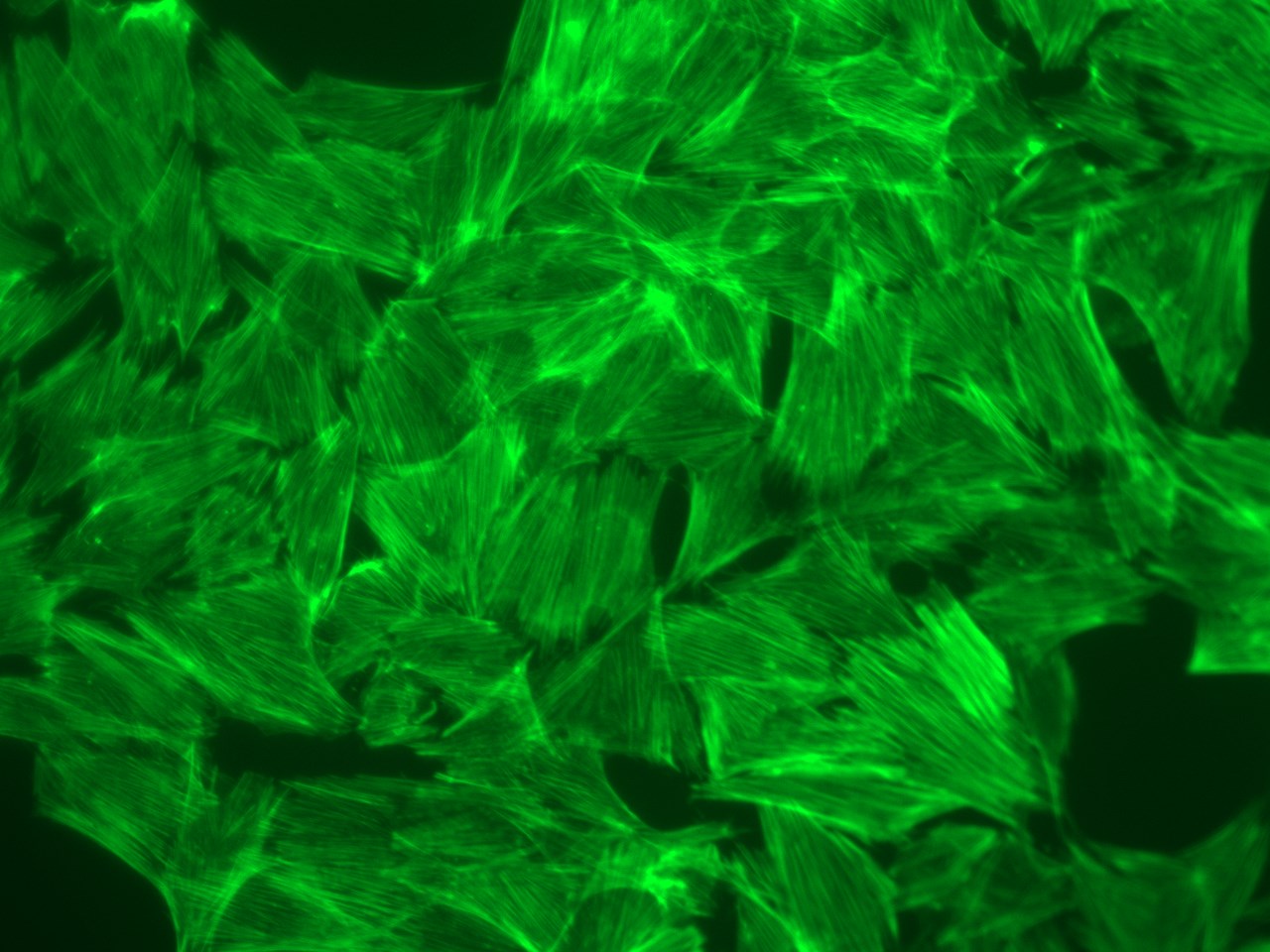

Supplement: Supplementary file 1 [file DataSheet1.zip › Pictures of Phalloidine/Control/0.890/87.223.jpg]

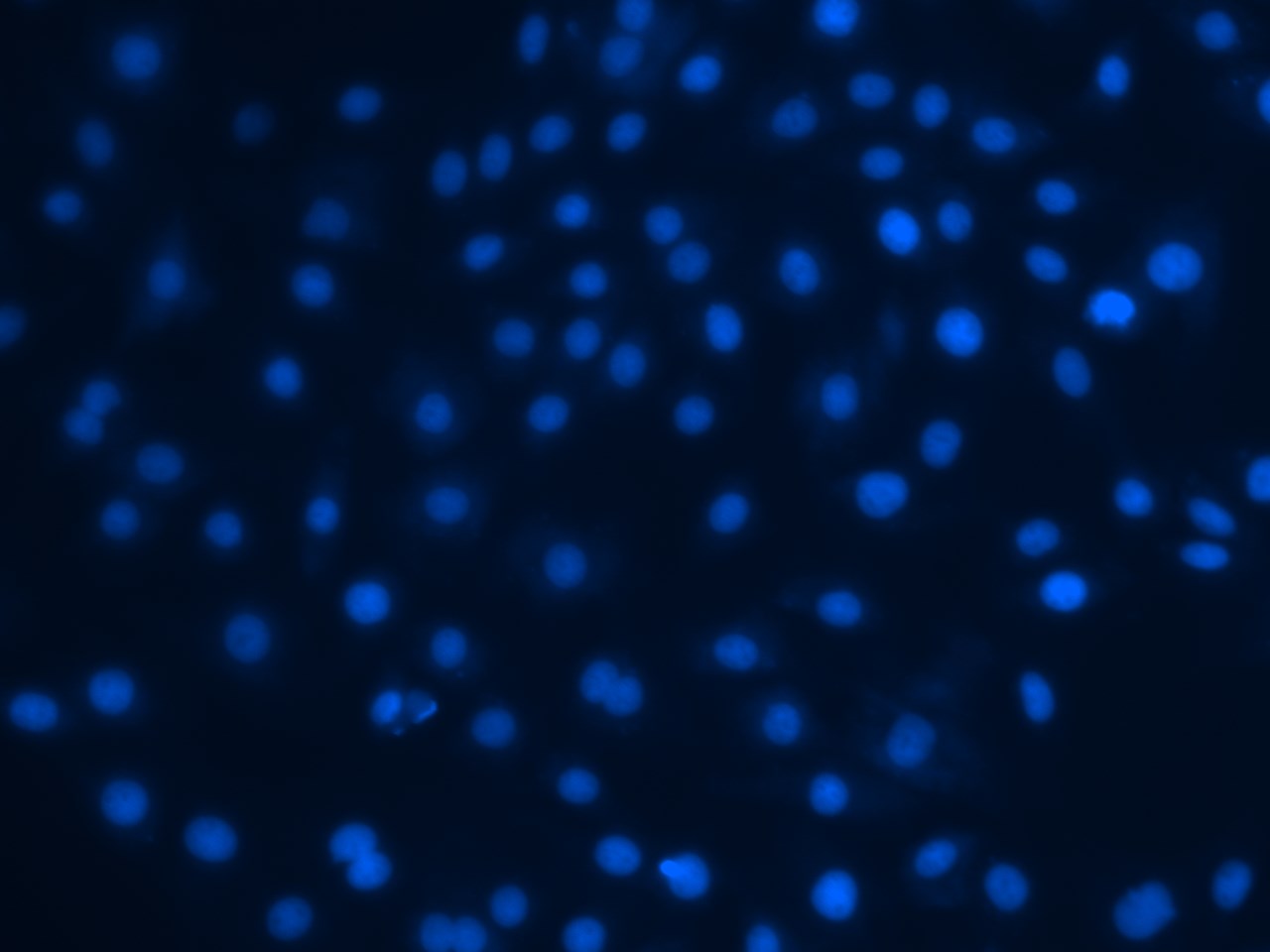

Supplement: Supplementary file 1 [file DataSheet1.zip › Pictures of Phalloidine/Control/0.890/98.jpg]

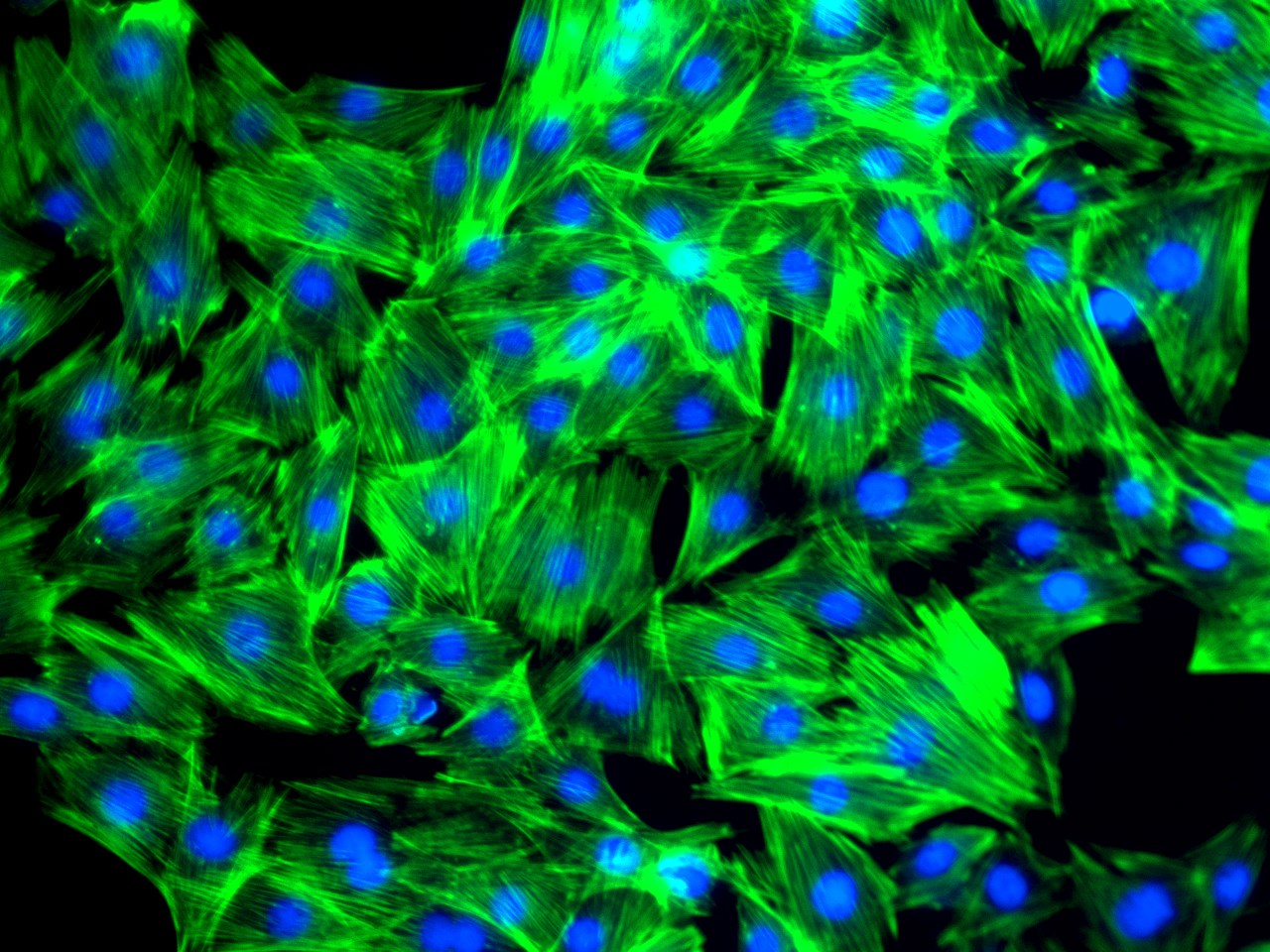

Supplement: Supplementary file 1 [file DataSheet1.zip › Pictures of Phalloidine/Control/0.890/Merge.jpg]

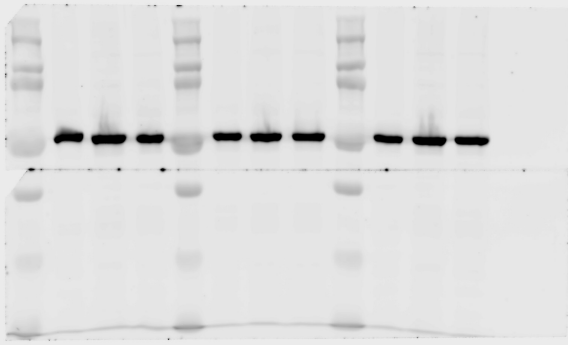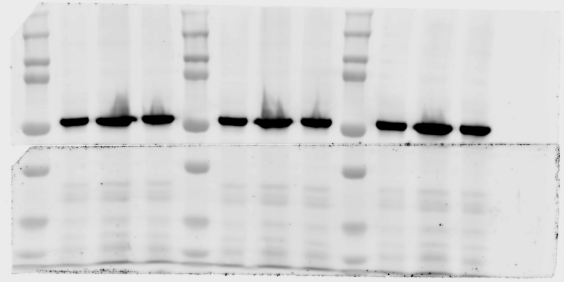

Supplement: Supplementary file 1 [file DataSheet1.zip › wb/0531-1 CMs IL6.pdf]

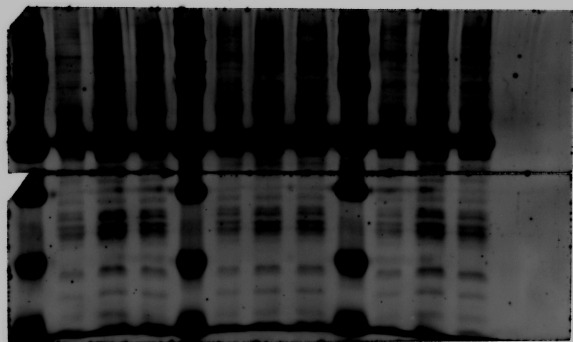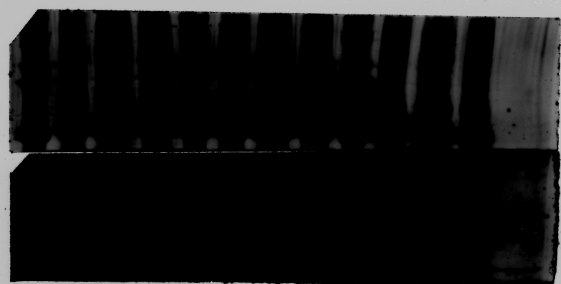

Supplement: Supplementary file 1 [file DataSheet1.zip › wb/0531-2 CMs IL-6.pdf]

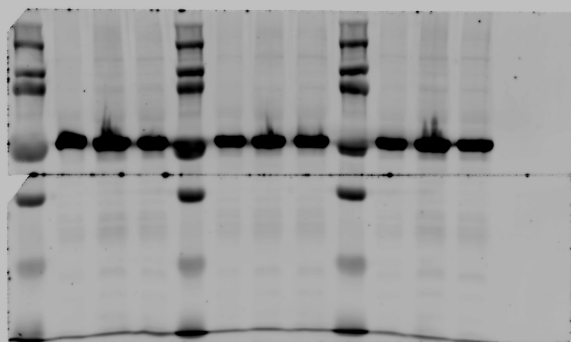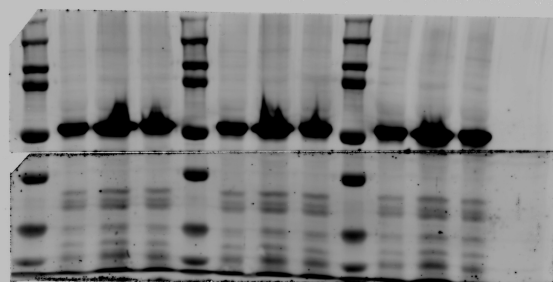

Supplement: Supplementary file 1 [file DataSheet1.zip › wb/0531-3 CMs IL-6.pdf]

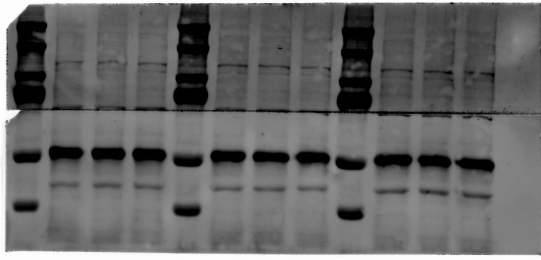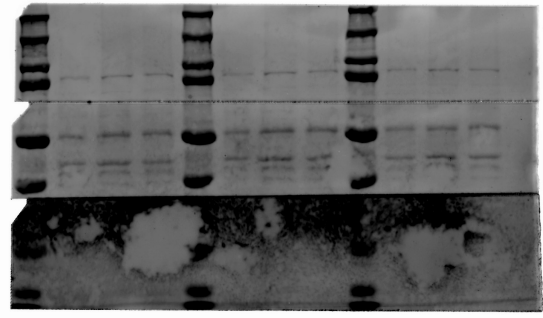

Supplement: Supplementary file 1 [file DataSheet1.zip › wb/WB CMs stat3 mapk1.pdf]

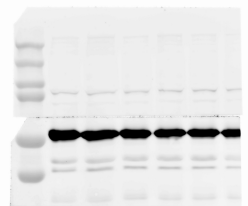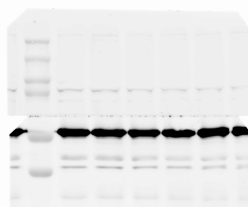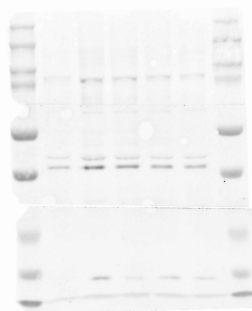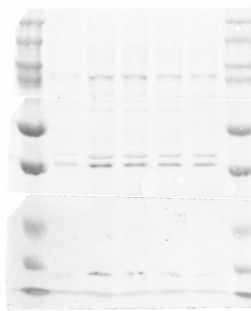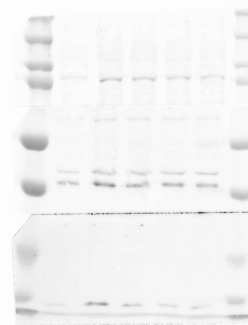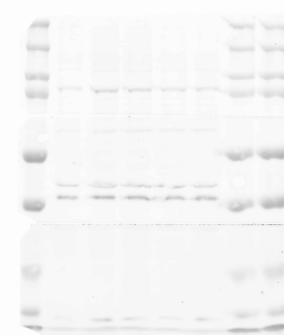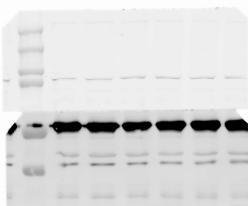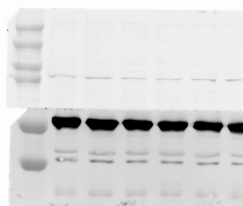

Supplement: Supplementary file 1 [file DataSheet1.zip › wb/WB H9C2 .pdf]
